# A Fine-Grained Image Dataset of Gold and Silver Artifacts from the Tomb of Prince Liangzhuang and Preliminary LoRA-Based Generation Experiments

**DOI:** 10.3390/s26196322

**Published:** 2026-10-07

**Authors:** Yelei Chen, Yifan Xu, Lei Shu, Ru Han

**Affiliations:** 1 Digital Art Industry Institute, Hubei University of Technology, Wuhan 430068, China; yifan.xu@hbut.edu.cn; 2 School of Smart Agriculture (School of Artificial Intelligence), Nanjing Agricultural University, Nanjing 210031, China; ruhan@stu.njau.edu.cn; 3 School of Engineering, University of Lincoln, Lincoln LN6 7TS, UK

**Keywords:** gold and silver artifacts, Tomb of Prince Liangzhuang, image dataset, LoRA technology, diffusion models

## Abstract

The digital preservation of cultural heritage is evolving from static archiving towards intelligent creation. As representative examples of early Ming Dynasty gold and silver craftsmanship, the gold and silver artifacts from the Tomb of Prince Liangzhuang, with their complex craftsmanship and rich cultural significance embodied in their decorative motifs, pose substantial challenges for digital preservation and innovative design. Generative artificial intelligence currently faces difficulties in reproducing fine-grained forms and decorative features, while the lack of high-quality, multi-dimensionally annotated image data specifically designed for such artifacts further constrains their digital preservation and application. To address these issues, this study constructed a fine-grained, multi-dimensional annotated image dataset of gold and silver artifacts from the Tomb of Prince Liangzhuang. The dataset covers five major categories, namely headwear, headgear and sashes, accessories, daily necessities, and ritual objects, comprising 82 artifacts and 4449 images, including 4127 panoramic images and 322 detail images. All images were annotated according to four visual feature dimensions: form and structure, decorative patterns, material appearance, and color composition. Based on this dataset, LoRA fine-tuning was performed on three mainstream diffusion models, namely SD1.5, SDXL, and FLUX. In the experimental evaluation, a total of 1968 images were generated for the 82 artifacts using the three models and eight random seeds (82 artifacts × 3 models × 8 random seeds). The generation results were systematically evaluated using quantitative metrics, including Structural Similarity Index Measure (SSIM), Learned Perceptual Image Patch Similarity (LPIPS), CLIP Score, and Kernel Inception Distance (KID), together with five types of subjective evaluation questionnaires, yielding 101 valid questionnaire responses in total. The results showed that all three LoRA-fine-tuned models were capable of generating images exhibiting typical characteristics of the gold and silver artifacts from the Tomb of Prince Liangzhuang. SD1.5_LoRA achieved the highest SSIM score (0.627) and the lowest seed-to-seed standard deviation (0.050), with statistically significant differences observed in most pairwise comparisons across categories after Bonferroni correction. SDXL_LoRA achieved the highest composite score in the subjective evaluation. CLIP Score showed category-dependent differences across the five artifact categories, and no consistent overall ranking of the three models was observed, suggesting comparable semantic alignment performance across the models. The resolution comparison experiment showed that, for the headwear category using SDXL_LoRA, no significant differences were observed across the evaluated metrics between the 768 × 768 and 1024 × 1024 resolutions. The results demonstrate that the proposed dataset can effectively support LoRA fine-tuning for the generation of gold and silver artifacts from the Tomb of Prince Liangzhuang. The three models showed different strengths in structural fidelity, semantic alignment, and material appearance, indicating that model selection can be tailored to specific application requirements. This study provides a high-quality data resource and a reusable technical approach for the digital preservation of Ming Dynasty gold and silver artifacts.

**Dataset:** The dataset has been released on IEEE DataPort, available at https://ieee-dataport.org/documents/dataset-gold-and-silver-artifacts-tomb-prince-liangzhuang (accessed on 3 October 2026).

**Dataset License:** Creative Commons Attribution (CC-BY) License

## 1. Introduction

**Figure 1:**
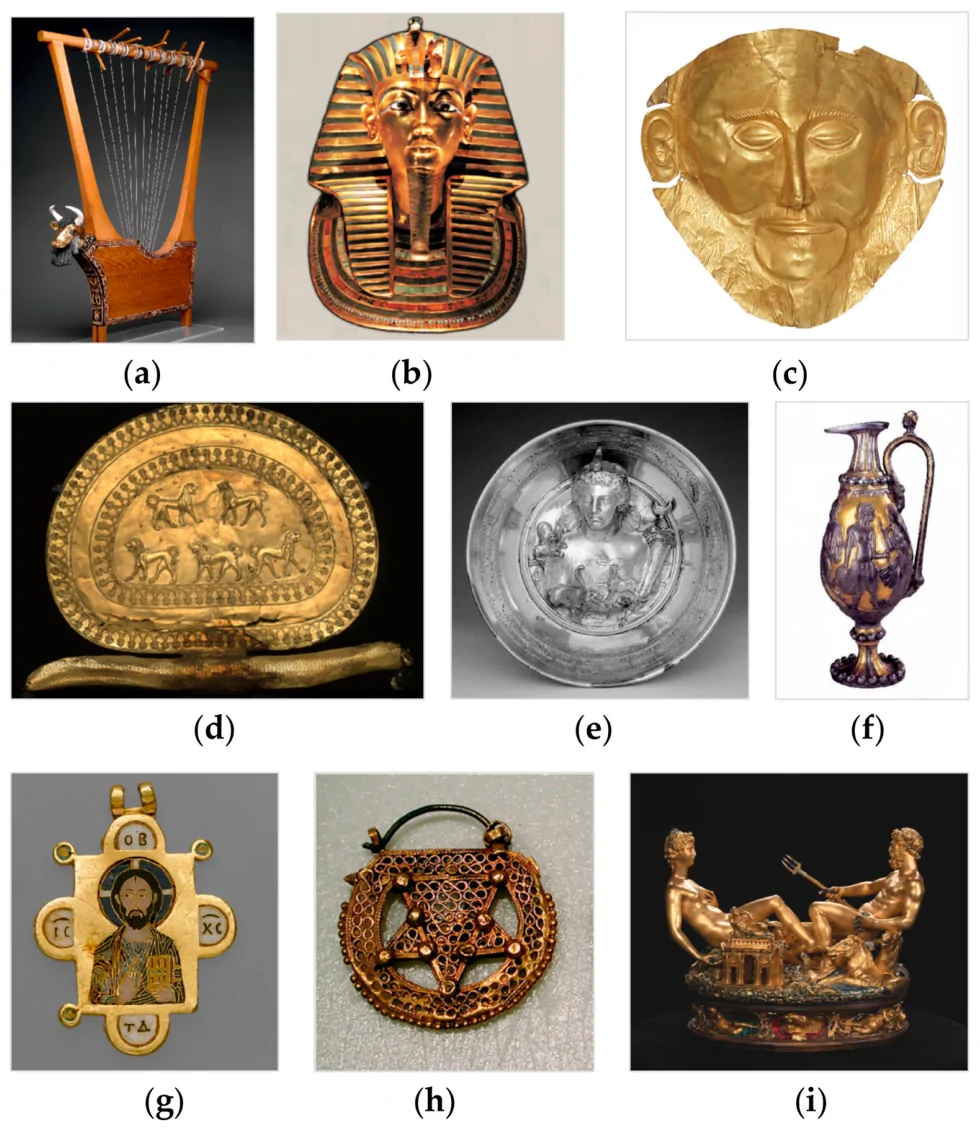
Examples of gold and silverware from various civilizations and regions around the world. (**a**) Golden bull-headed lyre from the Royal Tombs of Ur; (**b**) The golden mask of Tutankhamun; (**c**) Mask of Agamemnon; (**d**) Gold brooch from the Regolini–Galassi tomb; (**e**) Boscoreale silverware; (**f**) Gilded silver jug from the tomb of Li Xian at Guyuan; (**g**) Double-sided pendant icon; (**h**) Fatima gold earrings; (**i**) Cellini gold salt cellar.

Gold and silver artifacts are important manifestations of human material civilization. Beginning with Ancient Egypt around 5000 BCE and Mesopotamia around 4000 BCE, the tradition of gold and silver craftsmanship emerged and subsequently developed across regions such as Greece, Rome, Persia, South Asia, the Eurasian steppes, and China [1]. Throughout their development, gold and silver artifacts from these various regions not only developed distinctive craftsmanship and stylistic characteristics but also influenced one another through the exchange of technologies and trade. Ancient Egyptian gold and silver artifacts were closely associated with religious and funerary uses, making extensive use of hammering (a technique in which sheets of gold or silver are hammered to stretch and shape them), chasing (a technique in which patterns are carved into the metal surface using chisels), filigree (a technique in which gold or silver is drawn into fine wires, then woven and layered to form shapes), and gold beadwork [2]. The Sumerian civilization of the Fertile Crescent was already capable of producing gold helmets, gold and silver vessels, and jewelry inlaid with lapis lazuli during the Early Dynastic Period. Gold artifacts unearthed from the Royal Tombs of Ur demonstrate the high level of sophistication achieved in early filigree and granulation techniques [3]. Ancient Greek gold and silverware, spanning the Mycenaean to the Hellenistic periods, developed a technical tradition that evolved from hammering and chasing toward increasingly sophisticated applications of gold granulation and filigree [2]. Gold objects from the Classical and Hellenistic periods are particularly characterized by refined granulation and filigree techniques. Etruscan goldsmiths, meanwhile, further developed granulation (an ancient fine-gold technique involving the melting of gold into minute beads and their soldering onto the surface of metal objects to form decoration) and filigree techniques [2]. Gold and silver artifacts from different civilizations exhibit both shared characteristics and distinctive features in terms of form, decoration, and craftsmanship. Consequently, systematic digital research on gold and silver artifacts from a specific region can also provide useful methodological references for the digital study of similar artifacts from other regions. The characteristics and representative examples of gold and silver artifacts from different civilizations and regions are presented in Table 1 and Figure 1.

In Figure 1, (a) shows the golden bull-headed lyre from the Royal Tombs of Ur; source: British Museum https://www.britishmuseum.org/collection/object/W_1928-1010-1-a?selectedImageId=952425001 (accessed on 3 October 2026).

In Figure 1, (b) shows the golden mask of Tutankhamun; source: https://egyptianmuseumcairo.eg/historical-timeline/ (accessed on 3 October 2026).

In Figure 1, (c) shows the mask of Agamemnon; source: https://www.namuseum.gr/en/collection/syllogi-mykinaikon-archaiotiton/ (accessed on 3 October 2026).

In Figure 1, (d) shows a gold brooch from the Regolini–Galassi tomb; source: https://www.museivaticani.va/content/museivaticani/en/collezioni/musei/museo-gregoriano-etrusco.html (accessed on 3 October 2026).

In Figure 1, (e) shows the silverware from Boscoreale; source: https://www.louvre.fr/louvreplus/video-la-coupe-a-l-afrique-du-tresor-de-boscoreale (accessed on 3 October 2026).

In Figure 1, (f) shows a gilded silver jug from the tomb of Li Xian in Guyuan; source: https://www.nxgybwg.com/list/cul/1663088288218148866/344 (accessed on 3 October 2026).

In Figure 1, (g) shows a double-sided pendant icon; source: https://www.metmuseum.org/art/collection/search/466272 (accessed on 3 October 2026).

In Figure 1, (h) shows a pair of Fatima-themed gold earrings; source: https://www.metmuseum.org/art/collection/search/449683 (accessed on 3 October 2026).

In Figure 1, (i) shows a Cellini gold salt cellar (Saliera); source: https://www.khm.at/suche?q=Cellini+Salt+Cellar (accessed on 3 October 2026).

**Figure 2:**
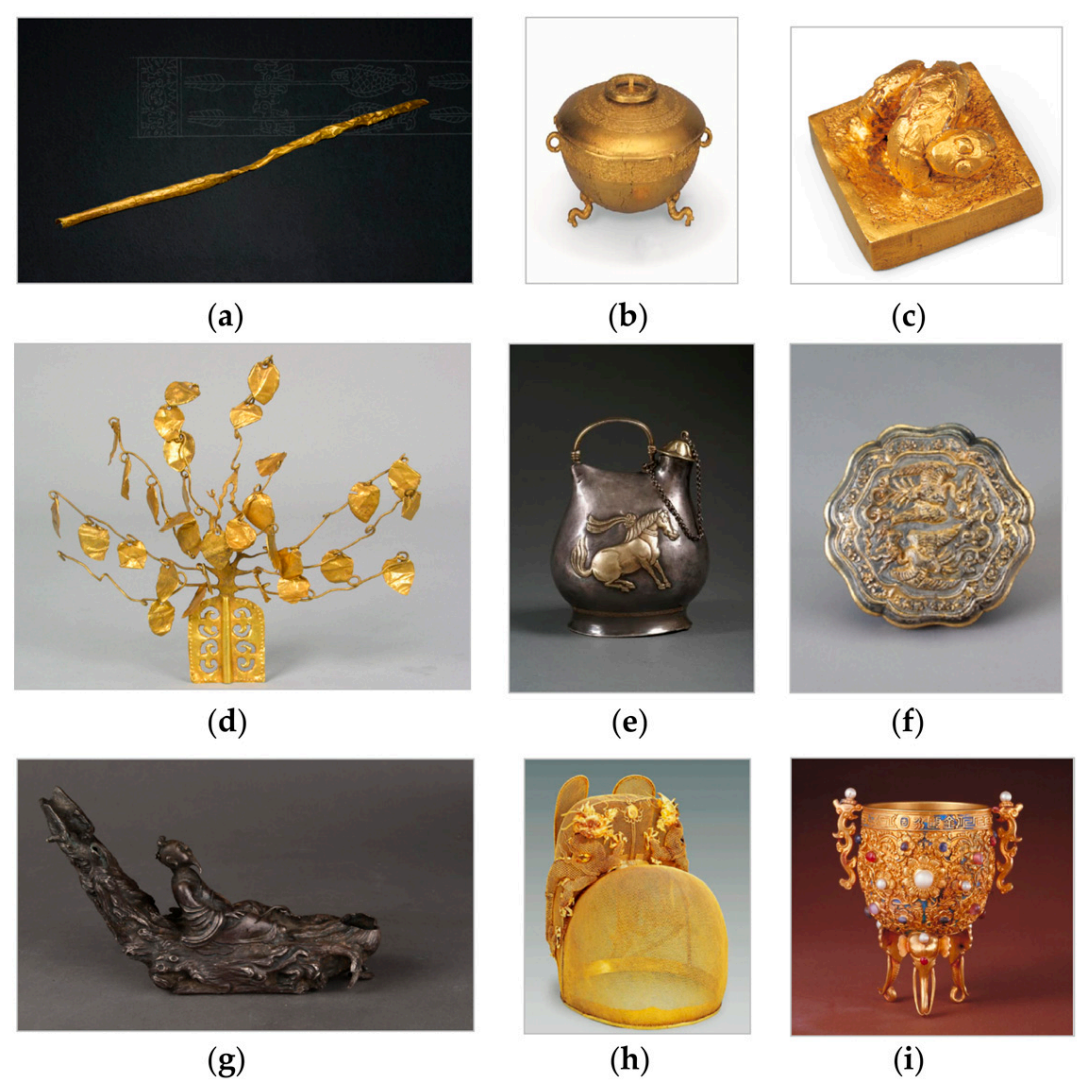
Examples of gold and silverware from various Chinese dynasties. (**a**) Sanxingdui gold staff; (**b**) Gold cup from the tomb of Zeng Hou Yi; (**c**) Golden seal of the King of Dian; (**d**) Gold hair ornament in the shape of a flowering tree; (**e**) Dancing horse holding a cup vase; (**f**) Southern Song Dynasty gilded silver box with double-phoenix motif in sunflower-petal shape; (**g**) Zhu Bishan’s silver ‘Driftwood’ cup; (**h**) The golden-threaded Yishan crown from Dingling; (**i**) ‘Eternal Stability’ gold cup.

In China, the use of gold and silver artifacts dates back to the Shang Dynasty, over three thousand years ago. Early gold and silver artifacts consisted mainly of small decorative items such as gold foil, gold leaves and gold plates, which were mostly used as appliqués on objects or as personal adornments. During the Spring and Autumn and Warring States periods, gold and silver craftsmanship underwent significant development, with the application of gold and silver inlay and filigree techniques flourishing. During this period, distinct stylistic differences emerged between northern and southern regions: the north favored animal motifs, while the south produced a greater number of functional objects such as vessels and belt hooks. During the Qin and Han dynasties, the opening of the Silk Road facilitated the introduction of foreign craftsmanship, and gold and silverwork gradually developed into a distinct craft tradition, with products ranging from ornaments to everyday utensils and medical instruments. During the Wei, Jin, and Southern and Northern Dynasties, the social functions of gold and silverware expanded further, with continuous innovation in forms and patterns, and the emergence of numerous artifacts incorporating foreign stylistic influences. The Tang Dynasty marked a particularly prosperous period in the development of gold and silverware, with highly sophisticated form, decoration, and craftsmanship. Building upon tradition, it extensively incorporated foreign cultural elements from sources such as Sasanian Persia and Sogdian culture, thereby forming a vibrant and distinctive style of its own. Gold and silverware from the Song Dynasty generally displayed a fresh and restrained esthetic. With the development of the commodity economy, the manufacture of gold and silverware became increasingly commercialized, and its user base gradually expanded to include broader segments of society, including the middle and lower social strata. The development of Chinese gold and silverware is summarized in Table 2 and Figure 2.

In Figure 2, (a) shows a gold scepter from Sanxingdui; source: https://www.sxd.cn/relics/detail?id=16880327704691380 (accessed on 3 October 2026).

In Figure 2, (b) shows a gold cup from the tomb of Duke Yi of Zeng; source: https://www.szbwg.net/show-18-40-1.html (accessed on 3 October 2026).

In Figure 2, (c) shows the gold seal of the King of Dian; source: https://www.chnmuseum.cn/zp/zpml/kgfjp/202110/t20211028_251978.shtml (accessed on 3 October 2026).

In Figure 2, (d) shows a gold hair ornament in the shape of a flowering tree; source: https://www.lnmuseum.com.cn/#/collect/detail?id=21010302862107B000096&pageType=3 (accessed on 3 October 2026).

In Figure 2, (e) shows a ‘Dancing Horse Holding a Cup’ ewer; source: https://www.sxhm.com/collections/detail/10117.html (accessed on 3 October 2026).

In Figure 2, (f) shows a Southern Song dynasty gilded silver box with a double-phoenix design in a sunflower-petal shape; source: https://www.fjbwy.com/collect/detail?id=8398 (accessed on 3 October 2026).

In Figure 2, (g) shows a silver ‘Zhu Bishan’ cup, source: https://wuzhongmuseum.com/portal/collection/content?id=fc8e646564202b0006171fe13d7ec378 (accessed on 3 October 2026).

In Figure 2, (h) shows the gold-threaded Yishan crown from Dingling, source: https://www.mingshisanling.com/relic/31.html?type=4 (accessed on 3 October 2026).

In Figure 2, (i) shows the ‘Golden Vessel of Eternal Stability’ cup; source: https://www.dpm.org.cn/collection/tinware/234538.html (accessed on 3 October 2026).

**Figure 3:**
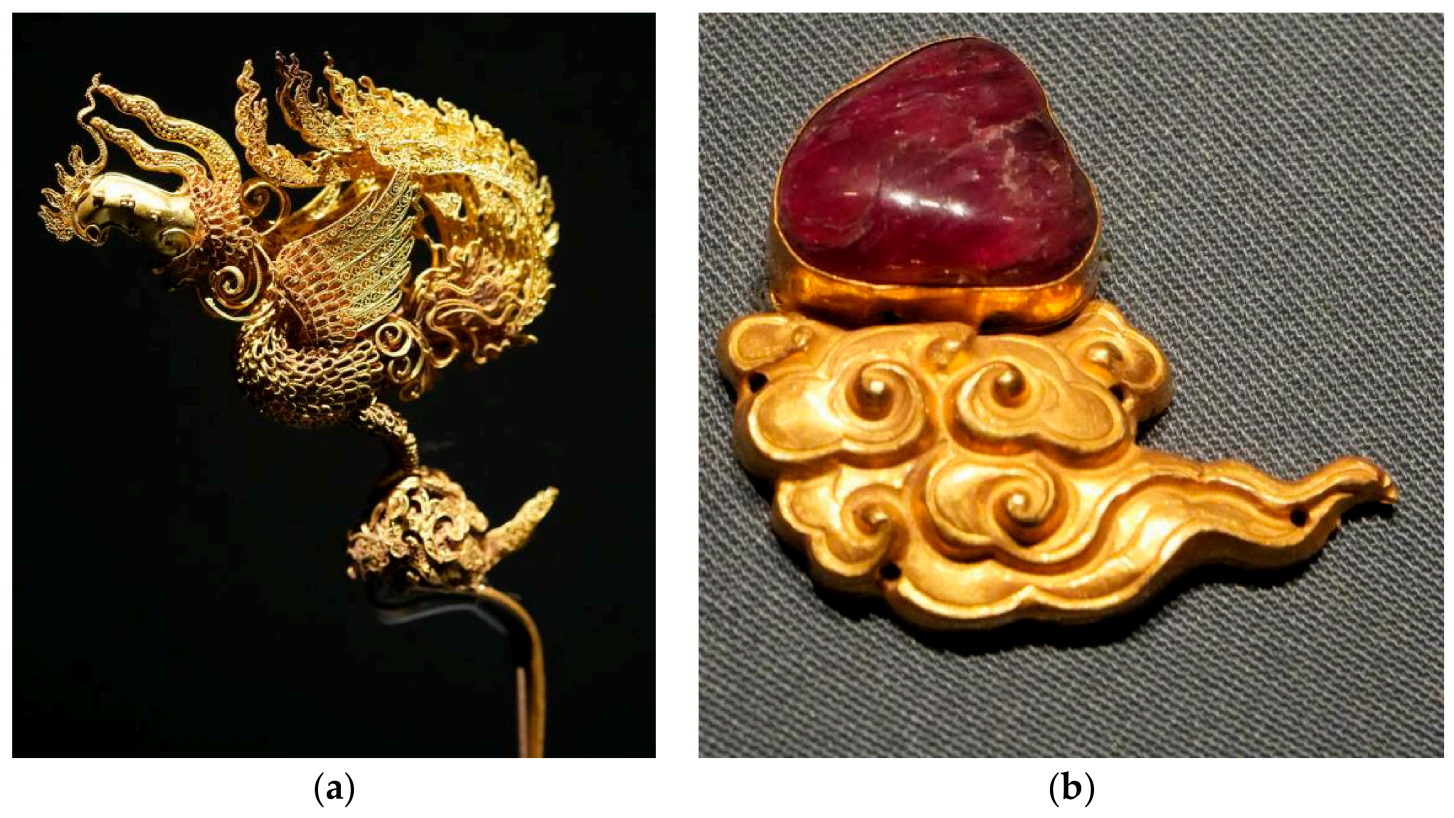
(**a**) Example of the gold-wire filigree technique: gold phoenix hairpin excavated from the tomb of the Prince of Liangzhuang in the Ming Dynasty; (**b**) example of gemstone inlay: cloud-shaped gold ornament set with gemstones.

By the Ming Dynasty, the development of Chinese gold and silverware had reached a stage where fine-gold and silver craftsmanship had incorporated and further developed techniques inherited from previous dynasties. Technically, inlay, filigree, hammering, and chasing techniques had become highly sophisticated and were often combined within individual artifacts, giving Ming Dynasty gold and silverware an overall appearance characterized by well-defined forms, intricate decoration, and rich material combinations [4]. Institutionally, the imperial household of the Ming Dynasty established organizations for the production of gold and silverware, including the Imperial Workshop, the Bureau of Palace Officials and the Silverworks Bureau. The products manufactured by these bodies adhered to established production standards and served the purposes of court ritual and the maintenance of hierarchical order [5]. Among these institutions, the Imperial Workshop was responsible for reviewing and approving the designs and styles of court gold and silver artifacts and determining their artistic schemes. The Bureau of Palace Affairs was responsible for overall casting and the production of large-scale objects, including the forming of complex gold and silver artifacts. The Silverworks Bureau was responsible for fine finishing and decorative work. From a cultural perspective, Ming Dynasty gold and silver artifacts served not only as material symbols of status and rank, but also as important material evidence for understanding the social structure and esthetic preferences of the period. Techniques such as gold-wire filigree and gemstone inlay exemplify the sophisticated level of Ming Dynasty gold and silver craftsmanship, and examples of these techniques can be seen in Figure 3.

Among Ming Dynasty gold and silver artifacts, those unearthed from the Tomb of Prince Liangzhuang hold significant research value and represent important examples of early Ming gold and silver craftsmanship. Prince Liangzhuang, Zhu Zhanji (1411–1441), was the ninth son of Emperor Renzong of the Ming Dynasty. His tomb is situated in present-day Zhongxiang City, Hubei Province, China. During archeological excavations in 2001, over 5300 artifacts, including gold, silver, jade and gemstones, were unearthed.The large quantity and high quality of the gold and silver artifacts make them an important group among the excavated tombs of Ming Dynasty princes. The artifacts unearthed from the tomb of the Prince of Liangzhuang provide important material evidence for understanding the political and economic conditions of in the early Ming period, maritime trade and cultural exchange, and the material culture of Ming princes. The gold and silverwares from this tomb encompass five major categories: headwear, headgear and sashes, accessories, daily necessities, and ritual objects. They exhibit a high degree of representativeness across four visual dimensions, namely form and structure, decorative patterns, material appearance, and color composition, making them a representative corpus for examining the craftsmanship and visual characteristics of Ming Dynasty gold and silver artifacts. Furthermore, the gold and silver artifacts unearthed from this tomb have long been on permanent display at the Hubei Provincial Museum. As the research team and the Hubei Provincial Museum are both based in Wuhan, the museum’s collection and location provide convenient conditions for repeated field observations and data collection. The image data for this study were obtained from the Hubei Provincial Museum’s publicly available digital resources and photographs taken on-site in the exhibition halls with the museum’s permission, covering a total of 82 artifacts.

However, while the gold and silver artifacts from the tomb of the Prince of Liangzhuang represent significant achievements in Ming Dynasty gold and silver craftsmanship, they currently face challenges in terms of digital preservation and intelligent application. Based on our review of publicly available resources,

- Regarding digital preservation, publicly available image resources of these gold and silver artifacts remain fragmented and lack standardized organization and fine-grained annotation. To the best of our knowledge, no publicly available image dataset specifically dedicated to the gold and silver artifacts from the tomb of the Prince of Liangzhuang has been identified, making it difficult to directly utilize these scattered images for data-driven research and model training;
- In terms of application, generative artificial intelligence, exemplified by diffusion models, exhibits issues such as deviations in vessel shapes, distorted patterns, and textural inaccuracies when reconstructing the intricate forms and decorations of gold and silver artifacts.

These two issues are not isolated; shortcomings in the digital preservation layer are one of the root causes of insufficient generation quality at the application layer. Therefore, improving the quality of generated images requires, first, the construction of a high-quality specialized image dataset to address the existing data gap, followed by an evaluation of its effectiveness based on the actual generation performance of the models.

To investigate the current state of research in this area, this study conducted a structured literature search covering the period from January 2010 to September 2026. The search used a combination of Chinese and English keywords related to the tomb of Prince Liangzhuang, gold and silver artifacts, hair ornaments, image datasets, annotation, diffusion models, LoRA, and generative AI. Chinese keywords were searched in China National Knowledge Infrastructure (CNKI) and Wanfang Data, while English keywords were searched in Web of Science, Scopus, and Google Scholar. After manual screening, and to the best of the research team’s knowledge based on the retrieved literature, no fine-grained multi-dimensional annotated image dataset specifically covering the gold and silver artifacts from the Tomb of Prince Liangzhuang was identified. In addition, no published generative AI experiments specifically targeting this type of gold and silver artifacts were identified.

Low-rank adaptation (LoRA) achieves domain-specific style transfer by injecting a low-rank decomposition matrix alongside the weights of a pre-trained model, requiring the training of only a very small number of parameters. It has been proven effective in small-sample cultural heritage image generation tasks, such as Miao batik [6] and Chu-style lacquerware [7]. Consequently, this study constructs a fine-grained annotated dataset for the gold and silver artifacts from the Tomb of the Prince of Liangzhuang. Based on this dataset, diffusion models are fine-tuned using low-rank adaptation techniques to ensure that the generated images better capture the typical characteristics of these artifacts.

Based on the above analysis, this study poses the following research questions: how can a specialized image dataset be constructed for the gold and silver artifacts from the Tomb of the Prince of Liangzhuang, and, building upon this, how can LoRA technology be utilized to effectively fine-tune diffusion models so that the generated images can accurately represent the visual characteristics of such gold and silver artifacts?

In response to this research question, this study undertakes the following work at the levels of digital conservation and application:

- At the digital conservation level, a dataset of images of the gold and silver artifacts from the Tomb of the Prince of Liangzhuang was constructed. Images were collected through a combination of publicly available resources from the Hubei Provincial Museum’s official website and on-site photography in the exhibition halls. Following image processing, a systematic annotation framework was established to complete fine-grained multi-dimensional annotation of the images.
- At the application level, LoRA fine-tuning and generation quality evaluation were conducted based on the proposed dataset. Three mainstream diffusion models, SD1.5, SDXL, and FLUX, were used as the base models, and LoRA fine-tuning was performed independently for each of the five artifact categories. The generated results were systematically evaluated from three perspectives: qualitative visual comparison, quantitative evaluation using SSIM, LPIPS, and CLIP Score as the primary metrics, with FID and KID treated as exploratory references given the limited sample size, and subjective evaluation through questionnaire surveys. In addition, a resolution validation experiment comparing 768 × 768 and 1024 × 1024 resolutions was conducted for the Headwear category. Finally, the potential application of the dataset in AIGC animation was further explored.

The main challenges encountered during the implementation of this study included the following:

- The visual features of gold and silver artifacts are complex. As there is currently no established international classification system for the visual features of such artifacts, the definition of feature dimensions requires expertise in archeology, design and computer vision. Following image processing, a systematic annotation framework was established, and fine-grained annotation was performed on the collected images.
- At the data collection stage, gold and silver artifacts are complex three-dimensional objects; the complete representation of their three-dimensional forms and details relies on multi-angle recording. However, the photography of cultural relics is constrained by museum exhibition conditions, making it difficult to standardize shooting angles, distances and lighting conditions; consequently, image information from different perspectives was collected as comprehensively as possible through multi-angle circular photography.
- In terms of model training, existing research on LoRA parameters has primarily focused on two-dimensional patterns, and there is a lack of clear reference points for the configuration of LoRA parameters for Ming Dynasty gold and silver artifacts. This study referred to existing training practices from other cultural heritage datasets to determine appropriate parameter settings. The three base models were then fine-tuned using LoRA, and their generation performance was evaluated experimentally to examine the effectiveness of the constructed dataset.

The objective of this study is to construct an image dataset of gold and silver artifacts from the Tomb of the Prince of Liangzhuang and to establish a fine-grained annotation system; on this basis, the LoRA method is utilized to fine-tune diffusion models, enabling them to generate images capture the typical visual characteristics of the gold and silver artifacts from the Tomb of the Prince of Liangzhuang based on textual descriptions. Building on this, this study also preliminarily explores potential applications of this method in the field of AIGC animation.

## 2. Related Work

### 2.1. Digitization of Cultural Heritage and Dataset Construction

The approach to the digital preservation of cultural heritage is gradually evolving from static archiving toward intelligent generation. In the existing research, digital recording and presentation remain important directions, including infrared thermography-based defect identification for cultural artifacts [8], which primarily focuses on the digital acquisition and archiving of cultural artifacts. At the same time, research attention has gradually expanded toward the intelligent restoration and innovative design of cultural heritage. Studies on the virtual restoration of Dunhuang murals [9], interactive experiences of indigo-dyed resist-dyeing techniques [10], and mobile crowdsourcing for urban cultural heritage [11] have explored different ways in which digital technologies can contribute to cultural heritage preservation, particularly in restoration and conservation, experiential dissemination, and documentation and archiving, respectively. These studies have primarily focused on preservation, restoration, documentation, and dissemination, with relatively limited attention to the controllable generation of cultural heritage features using generative artificial intelligence.

High-quality, standardized, specialized datasets provide an important foundation for cultural heritage digitization research and the cross-disciplinary application of artificial intelligence. Based on their primary research objectives, existing cultural heritage datasets can be broadly categorized into three types: identification and classification datasets, segmentation and 3D reconstruction datasets, and generation-oriented datasets. A comparison of these three types of datasets is presented in Table 3.

Identification and classification datasets include Heluo Musical Relics [12] and DeepJiandu [13]. Heluo Musical Relics contains musical artifacts, with annotated fields including artifact type, period, and cultural terminology. Its annotations primarily function as archival metadata for information retrieval and classification. DeepJiandu annotates the locations of characters rather than the visual features of the cultural artifacts themselves. Neither dataset provides detailed descriptions of artifact appearance, such as form, ornamentation, or material.

Datasets for segmentation and 3D reconstruction include YungangGrottoes3D [14] and the Ming-style furniture dataset MF2K [15]. YungangGrottoes3D reconstructs a millimeter-scale 3D model of Cave 13 of the Yungang Grottoes from images, with annotations describing the spatial morphology of the cave surface. MF2K contains 2073 images of Ming-style furniture, with annotations covering eight types of materials and environmental conditions. Its pixel-level masks delineate the boundaries of furniture within the images but do not describe attributes such as furniture form, decorative motifs, or specific material types.

Generation-oriented datasets include the CSL Dataset [7], CMBP-9 [6], and the Bamboo Weaving Pattern Dataset [16]. These datasets are particularly relevant to the present study because they provide annotations associated with the visual characteristics or generative representation of cultural heritage. The CSL Dataset contains 582 images of Chu-style lacquerware, with annotations covering vessel forms and patterns. CMBP-9 comprises 649 images of Miao batik, with an annotation framework spanning eight dimensions: category, compositional elements, descriptive semantics, hue, composition, visual symmetry, complexity, and traditional symbolic meaning. These dimensions encompass both visual characteristics, such as symmetry and complexity, and cultural semantics, such as symbolic meaning, providing relatively rich annotation information for culturally specific image generation. The Bamboo Weaving Pattern Dataset focuses on the distribution rules of patterns.

**Table 3:** Existing cultural heritage datasets.

Task Type	Dataset Name	Object of Preservation	Author and Year	Sample Size	Annotation Method
Recognition and Classification Dataset	Heluo Musical Relics	Musical artifacts from the Xia and Shang Dynasties to the Ming and Qing Dynasties	Zhao, J., 2021 [12]	216 images	Artifact type, period, and cultural terminology
	DeepJiandu	Qin and Han Dynasty bamboo and wooden slips	Liu, Y. et al., 2025 [13]	7416	Character bounding boxes, text categories
Segmentation and 3D Reconstruction Dataset	YungangGrottoes3D	Yungang Grottoes sculptures	Xiang et al., 2024 [14]	Over 280,000 images	Macroscopic 3D structure and form
	Image Segmentation of Ming-style Furniture	Ming-style furniture	Wan, Y et al., 2025 [15]	2073 images	Image segmentation, structural optimization
Style Generation Datasets	CSL Dataset	Chu-style lacquerware	Bi, H et al., 2025 [7]	582 images	Vessel shapes, patterns, textual descriptions
	Dong Ethnic Group Paper-cutting Dataset	Dong ethnic group paper-cutting	Xiao et al., 2025 [17]	130 items	Shape factors, semantic factors
	Lantern Art Dataset	Carved hollow lanterns	Zhou, C et al., 2025 [18]	18 types of patterns	Pattern distribution, color rules
	Peking Opera Face Paintings Dataset	Peking opera face paintings	Shen et al., 2024 [19]	1780 images	Face-painting images

In summary, the existing generation-oriented datasets reviewed above mainly focus on planar patterns or quasi-planar artifact surfaces and have not yet addressed gold and silver artifacts characterized by three-dimensional forms, complex craftsmanship, and fine decorative features. The image dataset of gold and silver artifacts from the Tomb of Prince Liangzhuang constructed in this study was developed to address this gap, with preliminary generation experiments conducted to examine its applicability to generative AI.

### 2.2. Applications of Generative Artificial Intelligence in Design

Applications of generative artificial intelligence in the cultural heritage field include style transfer and controllable generation, but evaluation approaches vary across different technical frameworks. As shown in Table 4, in a study using StyleGAN, bamboo weaving research combined StyleGAN with Grasshopper parametric modeling to generate new bamboo weaving patterns and subsequently applied them to product design. For diffusion models, a study on Miao batik used Stable Diffusion v1.5 with LoRA to transfer the structural, symbolic, and textural characteristics of batik patterns [6], while introducing a Latent Consistency Model (LCM) to improve inference efficiency. In another study on traditional lantern design, the AIlantern tool was developed based on diffusion models to generate renderings and unfolded patterns of openwork lanterns, and its effectiveness in conveying perceived cultural value was evaluated through expert and user assessments [18]. In addition, GuoFengAI developed an LoRA-based generative model for Chinese esthetic jewelry design and systematically presented a workflow covering the collection of traditional cultural elements, LoRA model training, and design generation [20]. In the area of 3D reconstruction, a study [21] reconstructed high-fidelity 3D Buddhist statues from single 2D images by modifying the Stable Diffusion architecture and incorporating ControlNet and the ISOMER algorithm. This study demonstrates the potential of diffusion models for 3D cultural heritage reconstruction. However, its subjects were Buddhist statues with relatively standardized structural forms and did not address finely crafted artifacts such as gold and silver artifacts.

Overall, the existing studies reveal two limitations in their evaluation and experimental design. First, cross-architecture comparisons remain limited. Some studies focus on a single generative model without conducting systematic comparisons across different diffusion architectures. Second, the influence of annotation schemes on generation quality has received limited attention. Most studies employ relatively simple annotation schemes, while few systematically investigate how different annotation dimensions affect generation performance. Even when detailed annotation schemes are adopted, their contribution to generation quality is not always experimentally examined. To address these gaps, this study establishes a multi-dimensional annotation framework for the gold and silver artifacts from the Tomb of Prince Liangzhuang and conducts LoRA fine-tuning experiments using three diffusion models, SD1.5, SDXL, and FLUX. Their generation results are then systematically compared to examine differences in generation performance and to evaluate the effectiveness of the proposed dataset and annotation framework.

### 2.3. Research on Gold and Silverware Worldwide

Research into gold and silver artifacts in the international academic community is primarily concentrated in the fields of archeology, archaeometallurgy and cultural heritage conservation. In terms of manufacturing techniques and craftsmanship, scholars have employed experimental archeology and materials analysis methods (XRF, SEM-EDS, micro-XRF, etc.) [22] to conduct systematic research into techniques such as metal forming, soldering, gold bead production and gilding. With regard to the provenance and circulation of materials, isotopic analysis has been employed to trace the origins of metals and trade networks [23]. In terms of regional case studies, a wealth of findings has been accumulated from analyses of gold and silver artifacts from Ancient Greece [24], Ancient Egypt [25], Mycenae [26], and the Eurasian Steppe. However, international research relies heavily on laboratory analytical equipment for the direct testing of physical artifacts and has paid comparatively limited attention to the application of computer vision approaches for the recognition and generation of visual features in gold and silver artifacts. In the fields of computational archeology and digital humanities, computer vision technology has begun to be applied to the classification, retrieval, and 3D reconstruction of cultural artifact images; however, existing work has largely focused on artifact types such as pottery, stone tools and coins, while systematic research on the visual feature recognition and image generation of gold and silver artifacts remains insufficient. A summary of global research trends in gold and silver artifacts is presented in Table 5.

Domestic research on gold and silver artifacts has primarily focused on archeology and the history of arts and crafts, as summarized in Table 6. In the field of archeology, scholars have established typological frameworks covering gold and silver artifacts from the Shang and Zhou dynasties through the Ming and Qing dynasties. In the history of arts and crafts, Yang Zhishui’s Shehua zhi Se: A Study of Gold and Silver Artifacts from the Song, Yuan, and Ming Dynasties [31] systematically examined the types and styles, decorative motifs, and manufacturing techniques of gold and silver jewelry from the Song, Yuan, and Ming dynasties. In terms of materials analysis, analytical techniques such as X-ray fluorescence (XRF) and scanning electron microscopy with energy-dispersive X-ray spectroscopy (SEM-EDS) have gradually been introduced in recent years. Yang Mingxing’s research team conducted scientific testing and spectroscopic analysis of jewelry and jade artifacts unearthed from the Tomb of Prince Liangzhuang [32].

Most research on gold and silver artifacts, both domestically and internationally, has primarily focused on archeology and the history of arts and crafts. A gap remains between the existing knowledge framework, which encompasses typology, descriptions of craftsmanship, and material composition, as well as the visual annotations required for computer vision tasks, such as form contours, decorative pattern distribution, material appearance, and color composition. The effective translation of existing archeological and art-historical knowledge into structured visual annotations is therefore needed.

### 2.4. Current Research on Gold and Silverware from the Tomb of the Prince of Liangzhuang

As a typical example of a feudal prince’s tomb from the early Ming Dynasty, the Tomb of the Prince of Liangzhuang yielded a large number of exquisitely crafted gold and silver artifacts, constituting vital physical evidence for the study of the Ming Dynasty’s gold and silver craftsmanship system and artistic styles. Existing research has primarily centered on three main areas: archeological excavation and documentation, analysis of the history of decorative arts, and scientific archeological analysis; however, systematic research on digital preservation and computational applications remains relatively underdeveloped, as detailed in Table 7.

In terms of archeological excavation and basic historical research, the excavation report on the Tomb of Prince Liangzhuang systematically documents the tomb structure, the composition of the burial goods, and information on the excavated artifacts [35]. In the field of the history of arts and crafts, Yang Zhao conducted a detailed interpretation and systematic analysis of the gold and silver artifacts unearthed from the Tomb of Prince Liangzhuang in his monograph ‘A Study of Gold and Silver Artifacts Unearthed from the Tomb of Prince Liangzhuang of the Ming Dynasty’ [36]. Taking the gold and silver artifacts from the tomb as the primary research subject, the monograph integrates perspectives from art archeology and scientific archeology and systematically examines the cultural context of gold and silver artifacts used by Ming princely households during the early and middle Ming Dynasty from the perspectives of materials, forms, craftsmanship, ornamentation, and inscriptions.

In terms of scientific archeology and materials analysis, Yang Mingxing’s research team conducted material identification and spectroscopic analysis of jewelry and jade artifacts unearthed from the Tomb of Prince Liangzhuang. In terms of digital preservation and practical applications, the Hubei Provincial Museum has carried out digital archiving of gold and silver artifacts from the Tomb of Prince Liangzhuang in its collection. Its online digital exhibition hall also features a dedicated section on the Tomb of Prince Liangzhuang, providing visual displays of basic information and images of selected core artifacts.

The work described above provides a solid foundation for understanding the craftsmanship and cultural significance of the gold and silver artifacts from the Tomb of Prince Liangzhuang. However, from the perspective of generative AI applications, specific gaps remain between the existing digitization resources and the requirements of model training. Based on the literature and resource survey conducted in this study, no publicly available image dataset specifically dedicated to the gold and silver artifacts from the Tomb of Prince Liangzhuang was identified. In addition, no published study was identified that had conducted LoRA fine-tuning experiments specifically using images of these artifacts.

This study addresses this specific gap by constructing a fine-grained annotated image dataset of gold and silver artifacts from the Tomb of Prince Liangzhuang. A four-dimensional annotation framework was established covering form and structure, decorative patterns, material appearance, and color composition. Based on this dataset, LoRA fine-tuning and generation experiments were conducted using three diffusion architectures: SD1.5, SDXL, and FLUX.

## 3. Methodology for Constructing the Gold and Silver Artifacts Dataset from the Tomb of the Prince of Liangzhuang

The construction of a dataset forms the foundation for deep learning and the intelligent generation of cultural relic features. In light of the complex visual characteristics of the gold and silver artifacts from the Tomb of the Prince of Liangzhuang, this chapter systematically outlines the methods for image data acquisition, processing and annotation, as well as the classification structure of the dataset.

### 3.1. Introduction to the Dataset

The image dataset of gold and silver artifacts from the Tomb of Prince Liangzhuang constructed in this study comprises two types of images. The first type consists of multi-angle panoramic images, which focus on the overall forms of the artifacts. A total of 4127 panoramic images were included to document the overall forms and visual characteristics of the artifacts. The second type consists of detail images cropped from the panoramic images, with a total of 322 images. The panoramic images provide an overall representation of artifact forms and categories, while the detail images supplement them with fine-grained information, including the composition of decorative motifs, visual characteristics of material surfaces, and color composition. The panoramic images constitute the core training data, whereas the detail images serve as an auxiliary detail reference library and were not used for model training.

The detail images were obtained through the following procedure. Researchers manually reviewed the panoramic images and selected and cropped representative detail regions, including filigree grids, inlay structures, and chased patterns. For each artifact, three to five representative visual features were selected. After deduplication and quality screening, 322 detail images were retained.

**Figure 4:**
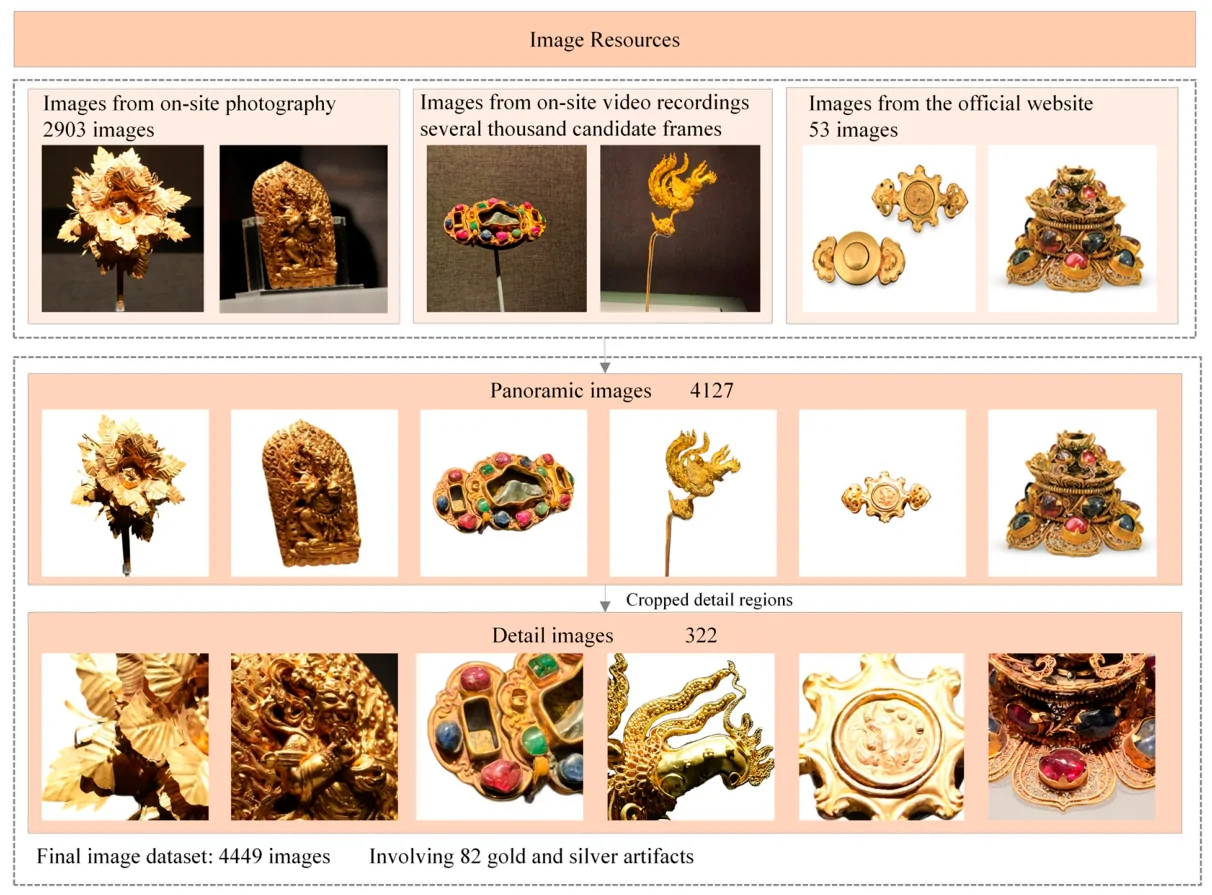
Composition of images of gold and silver artifacts from the Tomb of the Prince of Liangzhuang.

The image resources were acquired through three channels: publicly available digital resources from the official website of the Hubei Provincial Museum (53 images in the final dataset), photographic materials acquired by the research team during on-site photography in the museum exhibition halls (2903 images in the final dataset), and images extracted from on-site video recordings and subsequently screened and processed. The video recordings produced several thousand candidate frames, from which suitable images were selected for inclusion in the dataset. After image screening, processing, and deduplication, the final dataset comprised 4449 images, including 4127 panoramic images and 322 detail images. The composition of the dataset is shown in Figure 4.

Based on the functional classification framework for cultural artifacts documented in the excavation report The Tomb of Prince Liangzhuang and the use-oriented classification principle in archeological typology, the dataset was divided into five subsets:

- Headwear, comprising 800 panoramic images and 22 detail images from 11 artifacts, including hairpins, hair clips, hair ornaments worn at the temples, floral hairpins, phoenix-shaped hairpins, gold floral ornaments, and hair dividers;
- Headgear and Sashes, comprising 927 panoramic images and 143 detail images from 19 gold and silver artifacts, including cap tops, sash rings, gold belts, gold buckles, gold hooks, leather caps, ceremonial crowns, and jade belts;
- Accessories, comprising 800 panoramic images and 45 detail images from 13 gold and silver artifacts, including earrings, rings, bracelets, bangles, pendant beads, gold pendants, ritual rings, jade pendants, and gold ear spoons;
- Daily Necessities, comprising 800 panoramic images and 64 detail images from 28 gold and silver artifacts, including pots, basins, bowls, ritual cups, spoons, chopsticks, and boxes;
- Ritual Objects, comprising 800 panoramic images and 48 detail images from 11 gold and silver artifacts, including Buddha statues, vajra scepters, mantra plaques, deity statues, gold ingots, silver ingots, and gold coins.

**Figure 5:**
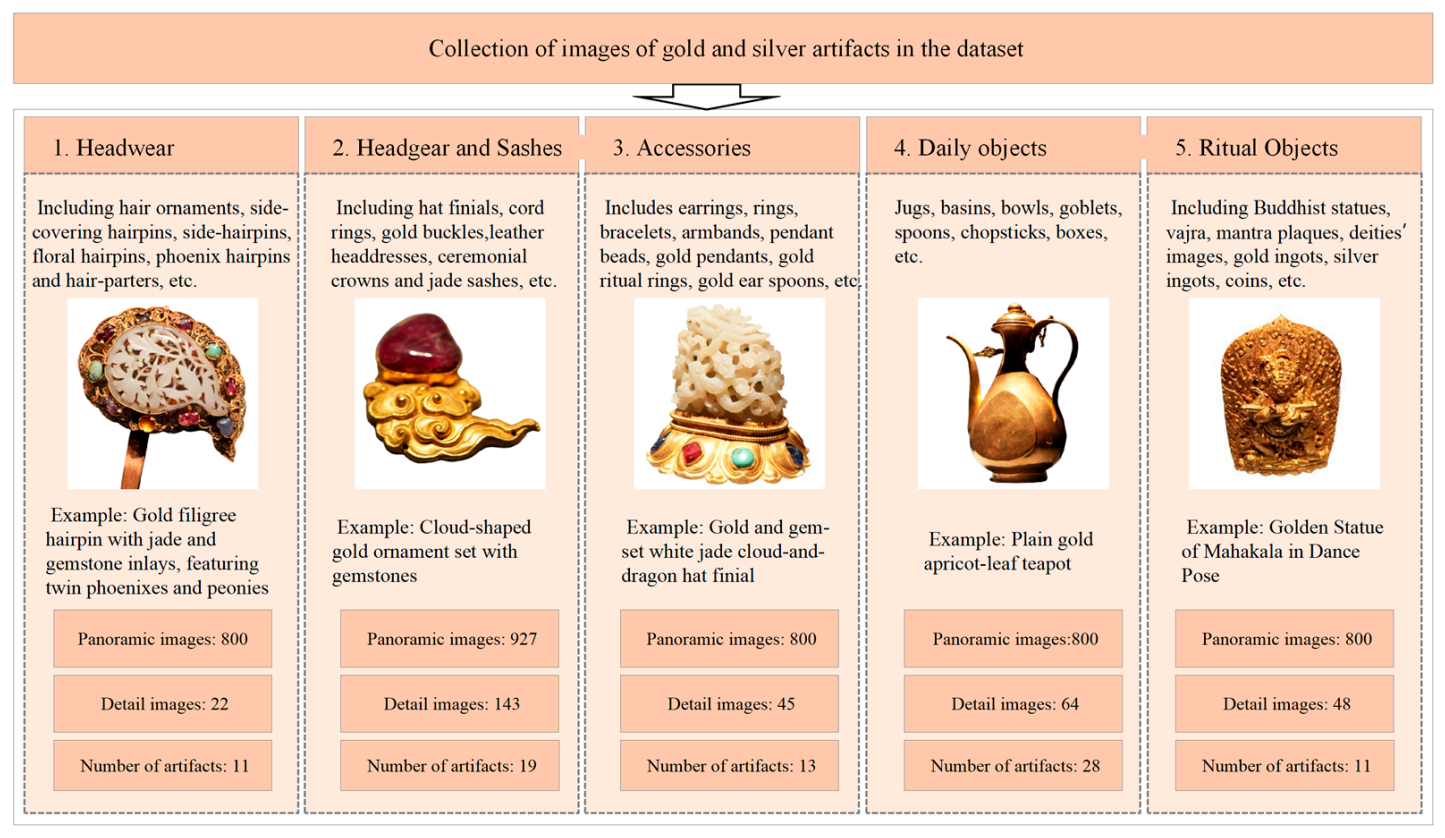
Classification of the Tomb of the Prince of Liangzhuang gold and silver artifacts image dataset.

Each subset maintains a complete correspondence between the images and their associated annotation files and is used independently for subsequent LoRA fine-tuning experiments. The classification system was developed by integrating the gold and silver artifact classification framework documented in the excavation report on the Tomb of Prince Liangzhuang, the classification approach established in Volume 2 of Yang Zhishui’s ‘Shehua zhi Se: A Study of Gold and Silver Artifacts from the Song, Yuan, and Ming Dynasties’, and the functional classification system presented in the Hubei Provincial Museum’s exhibition catalog. This integrated classification framework provides a systematic basis for organizing the dataset according to artifact type and function. The specific classification scheme and representative examples of the gold and silver artifacts are shown in Figure 5.

### 3.2. Data Collection

The image sources for the dataset comprise both online resources and on-site photographs, which complement one another.

Online resources primarily consist of image data of gold and silver artifacts excavated from the Tomb of the Prince of Liangzhuang, as collected on the official website of the Hubei Provincial Museum. The data from the official website is systematic and comprehensive, providing a relatively complete range of artifact types and basic information, totaling 53 images.

In terms of data quality, some images were professionally photographed for cultural heritage purposes, featuring high resolution and accurate color reproduction; however, others suffered from issues such as insufficient resolution, blurred edges and color distortion, making them unsuitable for direct use in fine-grained feature learning.

Our research team conducted systematic on-site photography in the Prince of Liangzhuang’s Tomb exhibition hall at the Hubei Provincial Museum. Using a Sony ILCE-7CM2 (α7C II) full-frame mirrorless camera (Sony Corporation, Tokyo, Japan), equipped with a Sony FE 90mm F2.8 Macro G OSS macro lens (Sony Corporation, Tokyo, Japan) and a Honor Play 4T Pro smartphone (Honor Device Co., Ltd., Shenzhen, China) as supplementary equipment, we captured the exhibited gold and silver artifacts from multiple angles—including the front, side, top view and oblique side—to ensure that each artifact was documented from different perspectives. Shooting followed the sequence of ‘panorama first, followed by detail capture’, beginning with images of the overall form of the artifacts before capturing close-up details of craftsmanship features such as filigree, chiseling and inlay. Care was taken during shooting to minimize issues such as reflections from the display case glass and uneven lighting in the exhibition hall. A total of 2903 images of gold and silver artifacts were acquired through on-site photography.

### 3.3. Data Processing and Standardization

**Figure 6:**
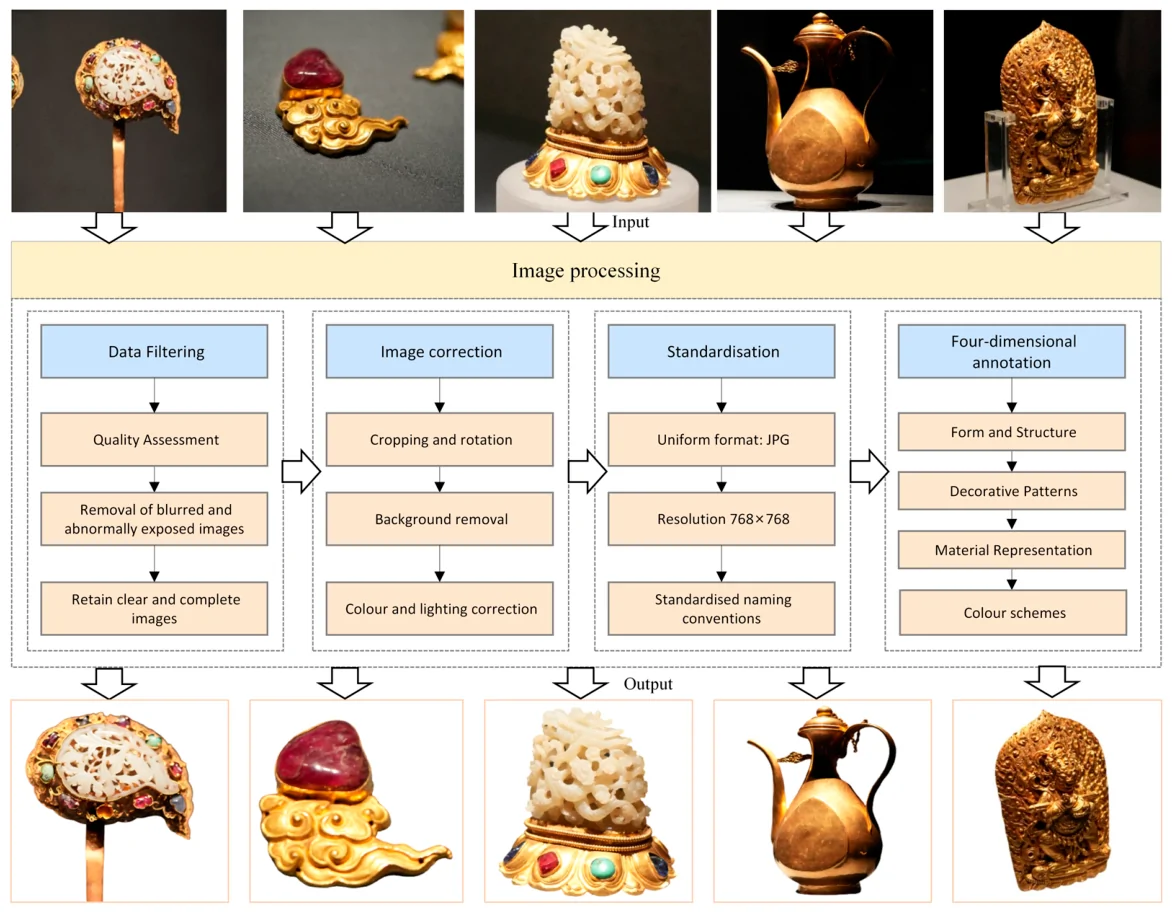
Image processing workflow for the image dataset of gold and silver artifacts from the tomb of Prince Liangzhuang.

The raw images obtained during the data collection phase presented issues such as diverse file formats, varying resolutions and inconsistent quality, and lacked standardized annotations, making them unsuitable for direct use in training deep learning models. Consequently, the systematic processing and annotation of the raw data were required. Data processing and annotation involved four stages: data cleaning, image standardization, annotation scheme development, and image annotation; the detailed workflow is shown in Figure 6.

#### 3.3.1. Data Cleaning and Image Correction

Firstly, the original images were manually assessed through visual inspection to exclude those with obvious quality defects. Exclusion criteria include images that are severely blurred, making the contours of artifacts and decorative details unrecognizable; key feature areas being obscured (e.g., artifacts obscured by display case frames or reflections); severe underexposure or overexposure resulting in loss of detail; and severe color casts or reflective interference that impedes the identification of decorative patterns. Retained images must meet basic requirements for clarity and integrity.

To address issues such as image blurriness, angular distortion and glare interference caused by the exhibition hall environment during on-site photography, the selected images are imported into Adobe Photoshop for editing and correction. The main operations include the following:

Cropping and rotation: Removing distracting elements such as display case frames and exhibit labels to ensure the gold and silver artifacts are the sole focus of the image;

Image masking: Removing distracting elements such as complex backgrounds to highlight the artifact itself;

Color and lighting adjustment: Adjusting parameters such as contrast, brightness, and exposure to correct color and restore the artifact’s true color;

Local sharpening: Sharpening specific details of the ornamentation to enhance the visual representation of craftsmanship features such as filigree and chiseling.

#### 3.3.2. Image Standardization

The original images were in a variety of file formats, such as JPG, PNG and HEIC. The JPG format is compatible with mainstream deep learning frameworks, offers high compression efficiency and retains sufficient detail at a reasonable compression ratio. To ensure data consistency, the batch conversion tool XnConvert was used to convert all images to the JPG format.

The standardization process utilized the Lanczos interpolation algorithm to uniformly adjust the image resolution to 768 × 768 pixels. This size preserves the fine details of intricate craftsmanship in gold and silverware, such as filigree and chiseling, while meeting the input image size requirements of subsequent models and avoiding the storage and computational burdens associated with excessively high resolutions.

#### 3.3.3. Image Annotation Process

Once data processing and standardization were complete, the original images had a uniform format and resolution, allowing the annotation phase to commence. The image annotation work in this study was carried out under the annotation framework described in Section 3.4; this section focuses on explaining the specific implementation process of the annotation.

Prior to annotation, the annotation language needed to be determined. Text encoders for diffusion models (such as CLIP) are trained on English corpora, and their semantic space is based on English; consequently, diffusion models generally demonstrate stronger semantic alignment with English descriptive terms than with Chinese expressions. Therefore, annotations are primarily in English.

However, the names of gold and silver artifacts and the terminology of craftsmanship possess strong cultural specificity; directly adopting English translations may result in semantic ambiguity or loss of information—for example, yanbin and binchai are two culturally specific hair ornaments that are both commonly translated as “hairpin” in English. If all annotations were in English, the model would be unable to distinguish between these different types of hairpins and hair ornaments, leading to confusion during training. Consequently, this study employs a hybrid ‘Pinyin + English’ annotation strategy: Pinyin trigger words are used to activate the LoRA model’s recognition of specific artifact categories, generated according to the rule ‘pinyin of the Artifact’s characteristic character + pinyin of the Artifact category name’. For example, for the ‘gold filigree hairpin with jade and gemstone inlays, featuring twin phoenixes, peonies and a hair-concealing design’, the pinyin luan’’from the character ‘luan’ is combined with ‘yanbin’ to form ‘luanyanbin’; different artifacts within the same category are distinguished by the pinyin of their characteristic characters. All Pinyin trigger words are unique within the dataset. English descriptive terms are used to guide the model in learning generalizable visual features (e.g., ‘cloud-shaped outline’ is annotated as ‘cloud-shaped outline’). This method draws on the multimodal dataset of ancient Chinese architecture compiled by Li et al. [38], which similarly employs a Pinyin-based annotation system for culturally specific terms such as ‘dou-gong’ and ‘hip-and-gable roof’ to bridge the semantic gap between Chinese and Western architectural terminology.

Once the annotation language has been determined, annotators proceed as follows:

- The annotators first examined each image and, based on the artifact type and its visual characteristics, selected the corresponding annotation items from each dimension of the four-dimensional annotation system established in Section 3.4;
- Enter the annotation terms into the annotation file in the format ‘Pinyin name of the cultural artifact + English four-dimensional annotation term’, separating each annotation item with a comma;
- For details where features are unclear or not covered by the standard system, judgments were made by comparing the original image with information from the Hubei Provincial Museum’s official website and relevant catalogs; supplementary explanations were added to the annotation fields when necessary.

Once all images have been annotated, each image was associated with a separate text-format annotation file, which contains all the annotation information for that image across the four dimensions: form and structure, decorative patterns, material representation and color coordination. The specific four-dimensional annotation system and examples are provided in Section 3.4.

### 3.4. Establishment of the Four-Dimensional Annotation System

Prior to the construction of the image dataset for the gold and silver artifacts from the Tomb of the Prince of Liangzhuang, there were no publicly available image datasets specifically dedicated to gold and silver artifacts, nor were there any systematic, standardized definitions of their visual characteristics. Disciplines such as archeology and design studies each place different emphases on the description of gold and silver artifacts: archeology focuses on the functional classification and morphological characteristics of objects, while the history of decorative arts concentrates on decorative patterns and manufacturing techniques. However, these disparate disciplinary descriptions had not been systematically integrated into a unified annotation system suitable for computer vision tasks. Consequently, this study required the synthesis of expertise from archeology, design studies, and computer vision to transform fragmented descriptions of gold and silver artifacts from these disciplines into visual feature dimensions that could be directly recognized by computer vision models.

#### 3.4.1. Literature Review and Dimension Synthesis

The archeological classification of excavated artifacts is function-oriented and follows the basic principle of ‘priority of use’. The excavation report on the Tomb of the Prince of Liangzhuang [35] classifies the excavated artifacts by material into categories such as goldware, silverware, jade objects and gemstones. The report classifies the artifacts by function (hairpins, hairpins with clasps, hair clips, jugs, basins, etc.) and provides detailed descriptions of the morphological characteristics and component structures of each artifact; Yang Zhao’s ‘A Study of Gold and Silver Artifacts Unearthed from the Tomb of the Prince of Liangzhuang in the Ming Dynasty’ [36] classifies the gold and silver artifacts excavated from the Tomb of the Prince of Liangzhuang by function, providing a systematic interpretation of the artifacts’ forms, decorations, manufacturing techniques and the material culture underlying them. Archeological functional classification provides a basic framework for the categorization of artifact types and materials in the construction of datasets. However, archeological classification focuses on functional roles and social significance artifacts, without providing a systematic visual description of the thematic affiliation of decorative themes, specific material compositions, or color relationships. Therefore, it requires integration with classification perspectives from other disciplines. Research in design studies on gold and silverware focuses primarily on the form, ornamentation and craftsmanship of the objects. Yang Zhishui’s The Colors of Luxury: A Study of Song, Yuan and Ming Gold and Silverware, Volume II systematically examines the types and styles of Ming Dynasty gold and silver jewelry, their decorative themes, as well as their pattern design and manufacturing techniques [31]. In terms of types, the book categorizes the objects into headwear, hairpins, ‘tiaoxin’, ‘fenxin’, ‘dian’, ‘yanbin’, ‘binchai’, earrings, bracelets and rings. Decorative patterns are grouped into categories such as flowers, plants and insects; fruits and vegetables; narrative scenes; and Sanskrit script. The book also provides a detailed account of the manufacturing techniques for gold and silver artifacts. The Hubei Provincial Museum’s The Tomb of the Prince of Liangzhuang: Treasures from the Age of Zheng He [37] provides expert descriptions of the materials (gold, silver, rubies, sapphires, emeralds, turquoise, white jade, etc.) and techniques (filigree, inlay, openwork, etc.) used in the artifacts, and offers a thematic summary of the esoteric buddhist artifacts found in the Tomb of the Prince of Liangzhuang; Zhang Yanfen’s research on the production organizations and technical methods of gold and silverware in the Ming Dynasty [5] systematically analyzes craftsmanship techniques such as hammering, chiseling, filigree, inlay and polishing. In the field of cultural heritage, Yi et al. [39] established a classification system for visual elements in Korean ceramics, categorizing artifact characteristics into five types: function, form, material, color and pattern. Among these, the four dimensions directly related to visual appearance, namely form, material, color, and pattern, provide a reference for the four-dimensional visual feature annotation framework proposed in this study.

In the field of computer vision, a vast body of research has systematically classified visual features of images. In reviews of feature extraction, scholars have categorized image features into five types: geometric features, statistical features, texture features, color features and conceptual features. Among these, geometric features capture the structural relationships and shapes of objects; statistical features describe the quantitative properties of pixel intensity distributions; texture features characterize the regular variations and repetitive patterns on the image surface; and color features model the color distribution of images using methods such as histograms and moment statistics [40]. In research on feature extraction from painted images, scholars have categorized image features as color, texture, light and shadow, brushstroke patterns, line style, geometric form and perspective relationships [41]. In the field of content-based image retrieval, scholars have systematically categorized three types of low-level features: color, texture, and shape [42]. With regard to material perception, Sharan et al. [43] proposed that material recognition is an independent task within computer vision. They argued that material categories can be identified from a single image through visual features such as color, texture, microtexture, and surface reflectance, and constructed the Flickr Material Database (FMD) to demonstrate that material is a learnable visual attribute independent of object shape and color.

This study synthesizes the classifications of cultural artifact features from the three aforementioned disciplines, drawing primarily on the classification system for Korean ceramics developed by Yi, J. et al. and incorporating the characteristics of gold and silver artifacts, [39], to formulate a four-dimensional feature annotation systemcomprising form and structure, material representation, color coordination, and decorative patterns, suitable for the gold and silverware from the Tomb of the Prince of Liangzhuang.

#### 3.4.2. Definitions of the Four Dimensions

Form and Structure: Refers to the external form of the artifact, the compositional relationships between its components, spatial hierarchy, and the expression of craftsmanship; this includes the category of artifact type, overall contour shape, the manner in which components are assembled, and the positional relationships between components.

Decorative Patterns: Refers to the decorative themes and pattern organization on the surface of the artifact, including subject categories (animal motifs, plant motifs, religious motifs, etc.), the form of pattern units, and layout methods (symmetry, repetition, scattered arrangement, etc.).

Material representation: Refers to the categories and properties of the materials used in the artifact, including metals, jade, and gemstones.

Color scheme: Refers to the compositional relationships among the color tones of the artifact, including the dominant color, accent colors, and the interplay between warm and cool colors.

#### 3.4.3. Establishment of the Annotation System

Based on the definitions of the above four dimensions, this study has established an annotation system as shown in Table 8. This system defines secondary and tertiary classifications for each dimension.

Taking the gold filigree jade-inlaid and gem-set ‘Double Phoenixes and Peonies Hairpin’ as an example, the full name of this artifact is too long in Pinyin; therefore, the abbreviated Pinyin ‘luanyanbin’ is used as the sole trigger term. Its specific annotations across the four dimensions are shown in Table 9.

The full annotation is as follows: luanyanbin, gold temple hairpin, cloud-shaped overall form, hollow-carved white jade center surrounded by gemstones, filigree wire mesh texture, hollow openwork texture, gem and jade bezel inlay texture, paired phoenix pattern, peony pattern, gold, white jade, turquoise, pink gemstone, lavender-blue gemstone, warm golden with white, turquoise, blue, green, pink, and lavender-blue accents

The image generation prompts for headwear-type cultural relics are provided in Appendix A. The complete image generation prompts for cultural relics across the five categories have been uploaded to the IEEE DataPort platform together with the dataset.

### 3.5. Preliminary Experiments on Annotation and Training Strategies

Preliminary experiments are required to verify whether the four-dimensional annotation system can effectively guide diffusion models in learning the typical features of gold and silver artifacts, and to assess how annotation strategies at different granularity levels affect the generation results.To this end, this study utilized Stable Diffusion v1.5 as the base model for the preliminary generation experiments, conducting comparative experiments on annotation strategies using a small dataset of headwear. Whilst keeping the model architecture and training parameters constant, only the level of detail in the annotation text and the composition of the training data were varied to observe changes in generation quality, thereby determining the optimal annotation strategy and training method.

#### 3.5.1. Experimental Design of the Preliminary Experiments

The experiments were divided into three groups—A, B, and C—each differing in annotation strategy and the composition of the training data. All experiments utilized the base Stable Diffusion v1.5 model with identical training parameters. The training data consisted of 162 panoramic images from the headwear subset, while a reference set of 11 headwear images was used for the qualitative evaluation of the generated results. The specific settings for the three experimental groups are shown in Table 10.

- Group A: Generalized Annotation

A generalized annotation strategy was adopted, whereby only core annotations for each dimension of the artifacts were provided, with no specific distinctions made between similar artifacts. The aim was to verify whether generalized annotation alone was sufficient for the model to learn the basic characteristics of gold and silver artifacts, while also comparing whether the inclusion of detailed images could yield additional benefits.

A1 (Training with panoramic images only): Training was conducted using only 162 panoramic images of headwear; these images were annotated with artifact type and outline.

A2 (Mixed training with panoramic and detail images): Training was conducted using a combination of 162 panoramic images and 22 detail images; the panoramic images were annotated with artifact type, while the detail images were annotated with detailed information across the four dimensions.

- Group B: Detailed Annotation

A detailed annotation strategy was adopted, adding image information such as symmetry, light direction, shooting angle, and color temperature to the four-dimensional annotation system, to examine whether annotating as thoroughly as possible could improve generation quality, while also re-evaluating the impact of detailed images.

B1 (Panoramic images only): Training was conducted using only 162 panoramic images of headwear; the panoramic images were annotated with the four-dimensional annotations and the aforementioned environmental variables.

B2 (Mixed training with panoramic and detail images): Training was conducted using a combination of 162 panoramic images and 22 detail images; both panoramic and detail images were annotated with four-dimensional information and environmental variables.

- Group C: Core four-dimensional annotations

Based on the experimental results from Groups A and B, Group C adopted a core four-dimensional annotation strategy, annotating only the essential visual features across four dimensions, form and structure, decorative patterns, material representation, and color coordination, while excluding all environmental variables. The training data comprised only 162 panoramic images, with no detailed images included. The purpose of Group C was to validate the effectiveness of a moderately focused four-dimensional annotation strategy.

#### 3.5.2. Preliminary Experiment Results

**Figure 7:**
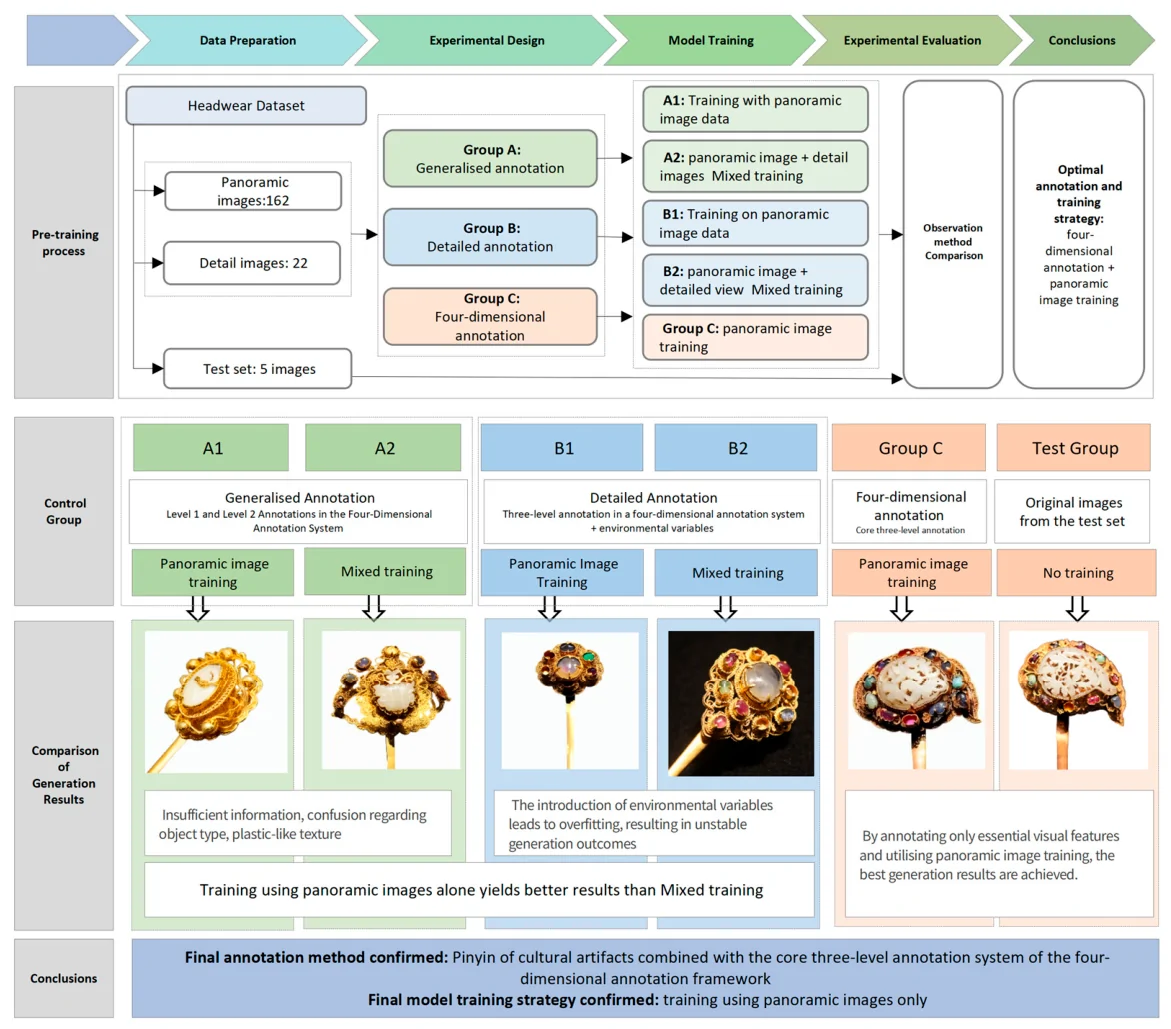
Pre-experiment workflow and results.

The preliminary experiment results are as follows; please refer to Figure 7 for the complete workflow and a selection of results.

A1 (Generalized annotations + panoramic images only): The model was able to generate objects that matched the outline and shape of the artifact, but there were instances of confusion between different features; the rendering of the material lacked texture, and the decorative lines were chaotic with unclear structure. The information provided by the generalized annotations was insufficient to guide the model in learning the complex visual features of gold and silver artifacts.

A2 (Generalized annotation + mixed panoramic and detail images): Following the inclusion of detail images, misalignments occurred between the decorative patterns and the structural form. This indicates that while detail images carry rich information regarding decorative patterns, the absence of overall structural constraints actually misled the model’s learning of the correspondence between decorative patterns and structural form.

B1 (Detailed annotation + panoramic images only): Excessive annotation information caused the model to overfit to non-essential photographic environmental features, such as color temperature and light direction, mistakenly learning these as object attributes. This resulted in significant deviations in form and color schemes, as well as incorrect rendering of materials.

B2 (Detailed annotation + mixed panoramic and detail images): The addition of detail images to the B1 setup exacerbated the issue of feature confusion.

C (Core four-dimensional annotation + panoramic images only): Models trained using four-dimensional annotation and panoramic images alone showed significant improvements across all dimensions: the contours of the structural forms were clear and the relationships between components were well-defined; the lines of the decorative patterns were fluid and the structures complete; the rendering of materials effectively reproduced the luster of metal and the texture of jade; and the color coordination was natural and harmonious. Core four-dimensional annotation covers the essential attributes that determine the visual identity of gold and silver artifacts, eliminating non-essential environmental interference and achieving optimal information density under the data conditions of this study.

Under the current annotation strategy and data ratio employed in this study, mixed training with detail images did not improve generation quality; rather, it interfered with the model’s learning of the correspondence between decorative patterns and structural forms. This result may be related to the lack of overall structural constraints in detail images and significant differences in scale; it does not imply that detail images themselves have no training value. Strategies such as phased training will be explored in future research.

#### 3.5.3. Preliminary Experiment Conclusions and Final Scheme Determination

Experiments in Group A showed that the information contained in generalized annotations was insufficient to guide the model in learning the complex visual features of gold and silver artifacts. Experiments in Group B demonstrated that overly complex, detailed annotations increased the model’s learning burden and exacerbated overfitting. Both sets of experiments confirmed that, regardless of whether general or detailed annotations were used, the quality of generated images declined rather than improved following the incorporation of detailed images into the training data. Meanwhile, Group C, which focused on annotations of the four key features and training using a panoramic image dataset, yielded the best image generation results. Based on these findings, the preliminary experiments established the final annotation strategy and training method.

With regard to annotation, the content should focus on the essential visual characteristics of the artifacts, adopting a method combining simplified Pinyin names of the artifacts with four-dimensional feature annotations in English. In terms of training, under the current annotation strategy and data ratio employed in this study, training with a mixture of detailed images did not yield any performance improvements; therefore, only panoramic images were used for this training session. Detailed images will be retained as a design resource library and for future research, but will not be used for model training; phased training schemes may be explored in the future to uncover the value of these detailed images.

### 3.6. Composition of the Final Dataset

Based on the final configuration established through the preliminary experiments, the image dataset of gold and silver artifacts from the Tomb of Prince Liangzhuang comprises 4127 panoramic images for model training and 322 detail images that are retained but not used for model training. The detail images primarily supplement the panoramic images by documenting local visual features of the gold and silver artifacts, such as filigree craftsmanship and gemstone-setting structures, and are not included in the model training process.

The detail images provide three main types of value:

Supplementary visual reference: They complement the panoramic images and enable researchers and readers to examine local characteristics of the gold and silver artifacts in greater detail.

Digital preservation and research resource: They provide high-resolution visual materials for subsequent research and digital preservation of cultural heritage.

Resource for future generation tasks: They provide a potential training data foundation for future fine-grained local generation tasks, such as decorative pattern generation and local form or structural generation.

Accordingly, the dataset can be characterized as an image dataset of gold and silver artifacts from the Tomb of Prince Liangzhuang centered on panoramic images and supplemented by an auxiliary detail reference library.

## 4. LoRA-Based Fine-Tuning Method for Diffusion Models

In this chapter, based on the Tomb of the Prince of Liangzhuang gold and silver artifact image dataset and with text-to-image generation as the primary task, LoRA technology is employed to fine-tune three diffusion models, with training and image generation carried out on the LiblibAI platform. The following sections detail model selection, data preparation, training parameters and the generation process.

### 4.1. Platform and Model Selection

This study selected three diffusion models—Stable Diffusion v1.5 (SD1.5), Stable Diffusion XL (SDXL) and FLUX—as base models. These represent three mainstream technical approaches for text-to-image models: SD1.5 serves as the baseline model for the classic UNet latent diffusion architecture; SDXL represents the dual-text-encoder-enhanced UNet architecture; and FLUX represents the rectified Transformer architecture. By training LoRA on each model using the same dataset, we can systematically evaluate the suitability of each model for the task of generating images of gold and silver artifacts.

LiblibAI (https://www.liblib.art) (accessed on 3 October 2026) is an AI image creation platform launched in 2023 by Beijing Qidian Xingyu Technology. Built upon the Stable Diffusion open-source technology stack, it provides online image generation and model training services for Chinese-speaking users. The platform integrates generative capabilities such as text-to-image, image-to-image and ComfyUI node-based workflows, and enables users to train LoRA models online, covering the entire process from dataset upload and prompt configuration to training parameter settings and model export. The official weights for all three models have been released on Hugging Face and can be accessed directly on the LiblibAI platform for online training. At the time of the experiments, the LiblibAI platform imposed a maximum of 600 images per LoRA training session and did not support continued training. These platform constraints directly determined the dataset partitioning and training strategy adopted in this study.

SD1.5 (available at https://huggingface.co/stable-diffusion-v1-5/stable-diffusion-v1-5/tree/main) (accessed on 3 October 2026) was proposed by Rombach et al. [44] at CVPR 2022. Based on a latent diffusion model architecture, it reduces computational costs by performing the diffusion process in a low-dimensional latent space rather than in pixel space. Its text encoder utilizes CLIP ViT-L/14, with a default output resolution of 512 × 512 pixels.

SDXL (available at https://huggingface.co/stabilityai/stable-diffusion-xl-base-1.0/tree/main) (accessed on 3 October 2026) was proposed by Podell et al. [45] in 2023 and is an upgraded version of the Stable Diffusion series. Compared to SD1.5, SDXL’s UNet has approximately three times the number of parameters and employs a dual-text encoder (CLIP ViT-L and OpenCLIP ViT-bigG), enhancing its ability to understand complex semantic descriptors; the default output resolution has been increased to 1024 × 1024 pixels.

FLUX (available at https://huggingface.co/black-forest-labs/FLUX.1-dev) (accessed on 3 October 2026) was publicly released by Black Forest Labs in August 2024. It utilizes a Rectified Flow Transformer architecture with approximately 12 billion parameters, replacing the UNet structure found in traditional diffusion models. By utilizing guided distillation techniques to achieve efficient mapping from noise to data, it demonstrates strong capabilities in rendering lighting, texture, and detail in generated images.

Regarding the selection of base models, Haoming Bi et al., in their Chu-style lacquerware dataset [7], systematically compared the performance of the aforementioned three models in cultural heritage image generation tasks, thereby validating the effectiveness of this comparative framework for cultural artifact style learning tasks. This study adopts this framework to evaluate the adaptability of different architectures to the complex features of gold and silver artifacts.

### 4.2. Preparation of Training Data

The dataset of gold and silver artifacts from the Tomb of the Prince of Liangzhuang, constructed in this study, encompasses five major categories, namely headwear, headgear and sashes, accessories, daily necessities, and ritual objects, comprising a total of 82 gold and silver artifacts, as presented in Table 11. Due to the LiblibAI platform’s limit of 600 images per LoRA training session, this study adopted a category-specific LoRA training strategy. For each category, SD1.5, SDXL, and FLUX were trained independently using exactly the same training images and training parameters, thereby ensuring that differences in generation performance among the three models could be attributed primarily to their architectural differences.

LoRA models were trained independently for each of the five artifact categories. For each category, the training set was constructed by stratified random sampling from the panoramic images of the corresponding category. First, images were selected to ensure that every artifact was represented in the training set. Additional images were then randomly sampled from the available images of each artifact until the training set reached the prescribed size for the category. Because the LiblibAI platform limits a single LoRA training session to 600 images, the training sets for four categories—headwear, headgear and sashes, daily necessities, and ritual objects—each contained 600 images. The accessories category contained 589 images after image screening and availability filtering. The five categories were trained independently and were not combined into a single training set. The resulting category-specific LoRA models were subsequently evaluated using their corresponding reference sets.

The reference set was constructed independently of the training data and was used to evaluate the generation quality of the trained models. For each artifact, multiple images from different viewpoints were selected as reference images. The reference set was not used during any stage of model training. The five category-specific reference sets contained approximately 200 images each, with a total of 1004 images covering all 82 artifacts.

The reference set and training set were derived from the same collection of artifacts but consisted of different images. Specifically, the reference images were selected from viewpoints different from those represented in the corresponding training images. A category-by-category comparison was conducted to verify that no reference image was included in the training set. Although the training and reference images followed the same naming convention, their image files were distinct, and no overlap was identified. Therefore, the evaluation conducted in this study measures the ability of the trained models to generate images corresponding to artifacts represented in the training data but from reference viewpoints not included in training. Accordingly, the evaluation should be interpreted as an assessment of generation performance for known artifact instances under different viewpoints, rather than as an assessment of generalization to previously unseen artifacts.

All training images were annotated according to the four-dimensional annotation framework established in Section 3.4, using the hybrid “Pinyin + English” annotation scheme. Each training image was associated with an individual structured annotation text file containing the corresponding artifact identifier and visual feature annotations. All training images were standardized to a resolution of 768 × 768 pixels before model training.

### 4.3. Training Parameter Configuration

All model training in this study was conducted on the LiblibAI platform; the main parameter configurations for each training scheme are shown in Table 12.

Rank (r) determines the intrinsic dimension of the LoRA low-rank decomposition matrix, i.e., the number of learnable parameters in the adapters. A lower rank results in more conservative model behavior; a higher rank enables the model to capture more complex features, but increases the risk of overfitting. Studies on two-dimensional patterns (such as Miao embroidery) typically set the rank to 32 [46]. Considering the relatively complex forms, decorative patterns, and material characteristics of the gold and silver artifacts in this study, the rank was set to 64, which was the highest rank that could be consistently supported across all three base models, SD1.5, SDXL, and FLUX, under the experimental conditions of the LiblibAI platform. Using the same rank for all three models also ensured consistency in the LoRA configuration for cross-model comparison.

The scaling factor Alpha controls the strength of the LoRA adapter’s influence on the base model. The higher the ratio of Alpha to rank (alpha/rank), the stronger the LoRA’s intervention in the generated results; the lower the ratio, the closer the output is to that of the original base model. In this study, Alpha is set to 32, resulting in alpha/rank = 0.5, thereby preserving the base model’s generative capabilities while learning features specific to the field of gold and silver artifacts.

The learning rate controls the magnitude of each parameter update. An excessively high learning rate may lead to unstable training, while an excessively low one results in slow convergence. In this study, the learning rate for UNet was set to 1 × 10^−4^, and that for the text encoder to 5 × 10^−5^ (half that of UNet), to prevent the text encoder from deviating from its original semantic understanding capabilities during fine-tuning.

Learning rate scheduling refers to a strategy of dynamically adjusting the learning rate during training, rather than using a fixed learning rate throughout. Appropriate scheduling enables the model to converge rapidly with a higher learning rate in the early stages of training, and to fine-tune with a lower learning rate in the later stages. This study adopts the Cosine Annealing strategy, whereby the learning rate gradually decreases from its initial value along a cosine curve until it approaches zero, which aids the model’s stable convergence.

Batch size refers to the number of images trained simultaneously in each iteration; this is set to two. Batch size is constrained by video memory; larger batches generally contribute to training stability but increase video memory consumption.

An optimizer is an algorithm that updates model parameters based on gradient information and minimizes the loss function during training; it determines the direction and magnitude of each parameter adjustment. This study employs AdamW8bit, which reduces GPU memory usage while maintaining optimization performance, thereby enabling the training of large models under limited computational resources.

Regarding the number of training steps, Xiao et al. [17] determined 40,000 training steps through preliminary experiments in their study of Dong ethnic paper-cutting. This value provides a reference for training-step selection in cultural heritage image generation tasks; however, the required number of training steps depends on factors such as dataset size, image repetition, batch size, and number of epochs. In the present study, each category contained approximately 600 training images, with each image repeated five times and the model trained for eight epochs. With a batch size of two, the approximate number of optimization steps for a category containing 600 images was therefore calculated as

600 × 5 × 8/2 = 12,000 steps.

For the accessories category, which contained 589 training images, the corresponding number of optimization steps was approximately

589 × 5 × 8/2 = 11,780 steps.

The same training configuration was applied consistently across the three base models within each artifact category to facilitate cross-model comparison. All training images were standardized to a resolution of 768 × 768 pixels.

### 4.4. Implementation of the Training Scheme

Based on the conclusions of the preliminary experiments presented in Section 3, only panoramic images were used during the formal training stage, while detail images were excluded from model training. The training datasets for the five artifact categories were uploaded separately to the LiblibAI platform. For each category, three pre-trained base models—SD1.5, SDXL, and FLUX—were independently loaded, and LoRA fine-tuning was performed using the parameter configuration described in Section 4.3.

**Figure 8:**
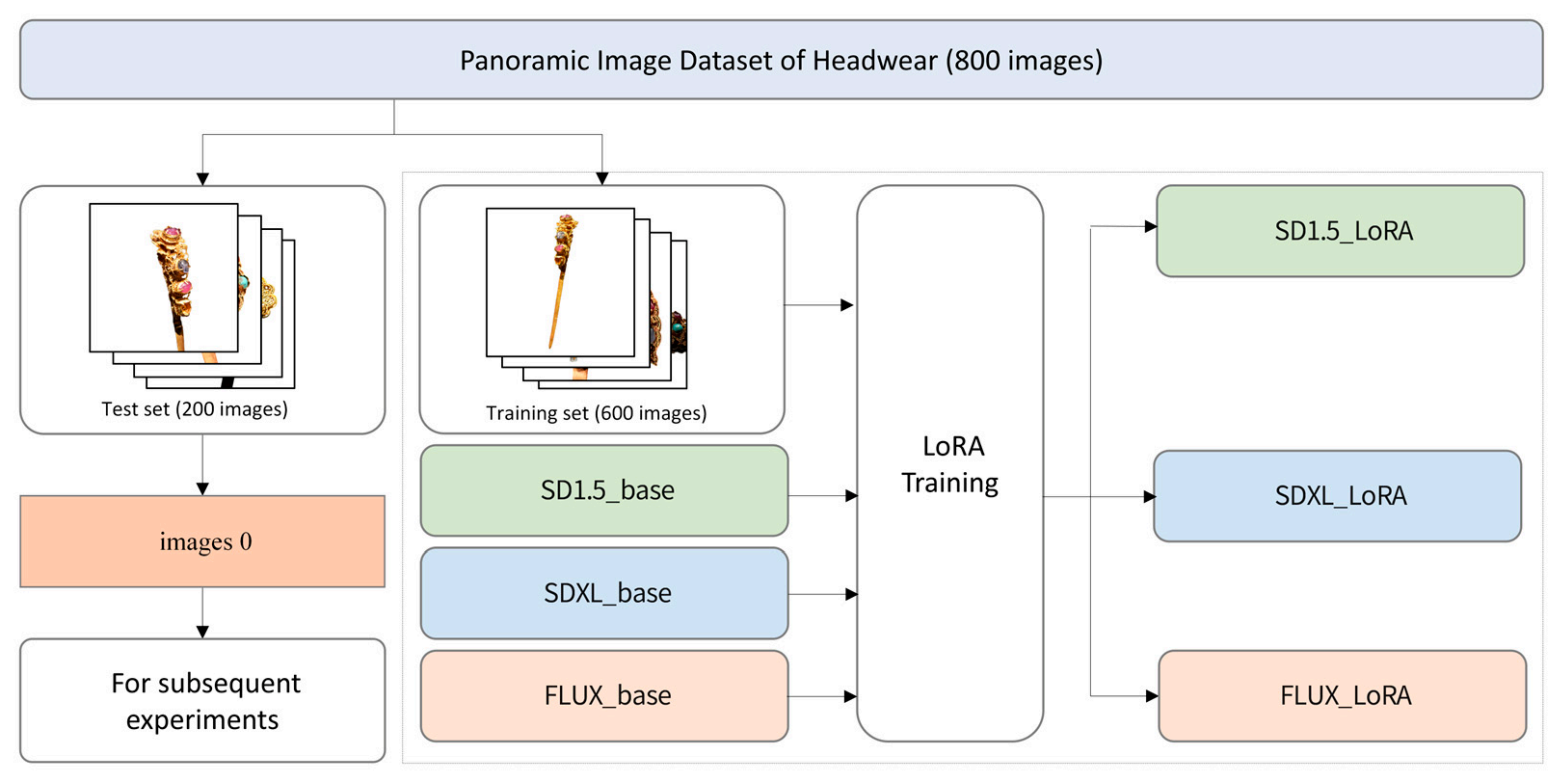
Training workflow for the headwear panoramic image dataset.

This training protocol produced a total of 15 LoRA weight files, corresponding to the five artifact categories and three base models (five categories × three models). The files were named according to the format “Category_BaseModelName_LoRA”, with examples including “Headwear_SD1.5_LoRA”, “Headwear_SDXL_LoRA”, and “Headwear_FLUX_LoRA.” The division of the training and reference sets is illustrated in Figure 8. Subsequent figures and result analyses use the headwear category as an example unless otherwise specified.

The reference set images are labeled as ‘images 0’ and comprise 11 images of cultural artifacts. These are used for subsequent experiments and are distinguished from the images generated by different models, labeled as ‘images 1–6’.

There is one architectural difference between the three models during training: SD1.5 and SDXL are based on the U-Net diffusion architecture, which supports hierarchical fine-tuning of the U-Net backbone and text encoder; consequently, they adopt the hierarchical learning rate strategy described in Section 4.3. FLUX employs a unified Transformer architecture, with no separate UNet and text encoder branches; consequently, it utilizes a global, uniform learning rate. This difference stems from inherent constraints in the model’s underlying architecture and is not an artificially introduced control variable. Whilst maintaining other parameters consistent with those of SD1.5 and SDXL, the global learning rate for FLUX was set to 5 × 10^−5^.

After training, the LoRA models were used to generate images corresponding to the artifacts represented in the reference set. For each artifact, the LoRA model trained for its corresponding artifact category was applied to the generation process. The image generation parameters were kept consistent across the three models: the DPM++ 2M sampler, 30 sampling steps, CFG scale of 7.0, 768 × 768 output resolution, and an LoRA weight of 0.7. To examine the stability and variation in the generated results across different random initializations, each test prompt was used to generate images with eight fixed random seeds (42–49). The same seed sequence was used for all three models to ensure a controlled comparison and reproducibility.

For each of the 82 artifacts, one corresponding test prompt was used for each of the three category-specific LoRA models, and eight images were generated using the fixed seed sequence. Thus, the complete generation set comprised 82 artifacts × three models × eight seeds = 1968 generated images.

## 5. Experiments

### 5.1. Experimental Overview

Based on the training methods described in Section 4, this chapter systematically evaluates the performance of the LoRA-fine-tuned models in generating images of gold and silver artifacts from the Tomb of Prince Liangzhuang. The experiments are conducted from four perspectives.

Visual Comparison Analysis (Section 5.2). An observational approach is used to compare generated images within the same artifact category. The analysis first compares the results generated by the base models and their corresponding LoRA-fine-tuned models to examine the effect of LoRA fine-tuning. It then compares the three LoRA-fine-tuned models to examine their differences in representing the four visual feature dimensions.

Quantitative Evaluation (Section 5.3). Structural Similarity Index Measure (SSIM), Learned Perceptual Image Patch Similarity (LPIPS), CLIP Score, and Kernel Inception Distance (KID) are used to quantitatively evaluate the generated images. Bootstrap confidence intervals are calculated to characterize the uncertainty of the metric estimates, and the Wilcoxon signed-rank test is used to assess pairwise differences between the models.

Subjective Evaluation Using the Reference Set (Section 5.4). A questionnaire survey is conducted among relevant practitioners to evaluate the generated images from the perspective of human perception. The evaluation focuses on the four visual feature dimensions. The Friedman test is used to assess whether the rankings of the three models differ significantly.

Resolution Validation Experiment (Section 5.5). Based on the results of the preceding experiments, a selected LoRA-fine-tuned model is used to compare generation results at different resolutions. This experiment examines whether the evaluated metrics differ between the tested resolutions and provides a basis for determining the final generation configuration.

The experiments are conducted in a progressive manner. Visual comparison provides an initial qualitative assessment of the generated results. Quantitative evaluation provides metric-based evidence. Subjective evaluation incorporates human perceptual assessment. The resolution validation experiment further examines the effect of output resolution on generation performance.

### 5.2. Visual Comparative Analysis of Generation Results

This section employs an observational approach, using visual comparisons of images generated from the same prompt by different models to form a preliminary assessment of the performance differences between them.

#### 5.2.1. Design of Comparative Experiments

The visual comparison was conducted using the artifacts represented in the reference set and was organized by artifact category. The analysis consisted of two stages.

The first stage was a vertical comparison between each base model and its corresponding LoRA-fine-tuned model. For the same artifact and the same prompt, the generated results before and after fine-tuning were compared within each model family. The comparisons were conducted between SD1.5_base and SD1.5_LoRA, SDXL_base and SDXL_LoRA, and FLUX_base and FLUX_LoRA. This comparison was used to examine whether LoRA fine-tuning improved the representation of the visual characteristics of the target gold and silver artifacts.

The second stage was a horizontal comparison among the three LoRA-fine-tuned models. The generated results of SD1.5_LoRA, SDXL_LoRA, and FLUX_LoRA were compared for artifacts within the same category. The comparison focused on differences in the representation of the four visual feature dimensions, namely form and structure, decorative patterns, material appearance, and color composition.

**Figure 9:**
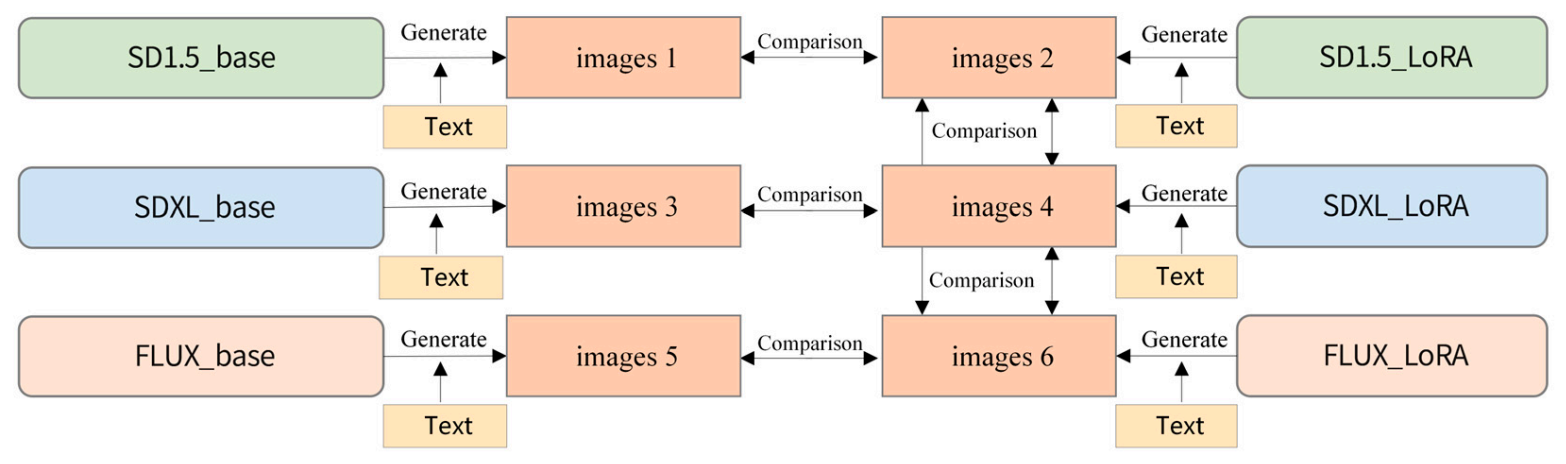
Experimental workflow for comparison of generation results.

**Figure 10:**
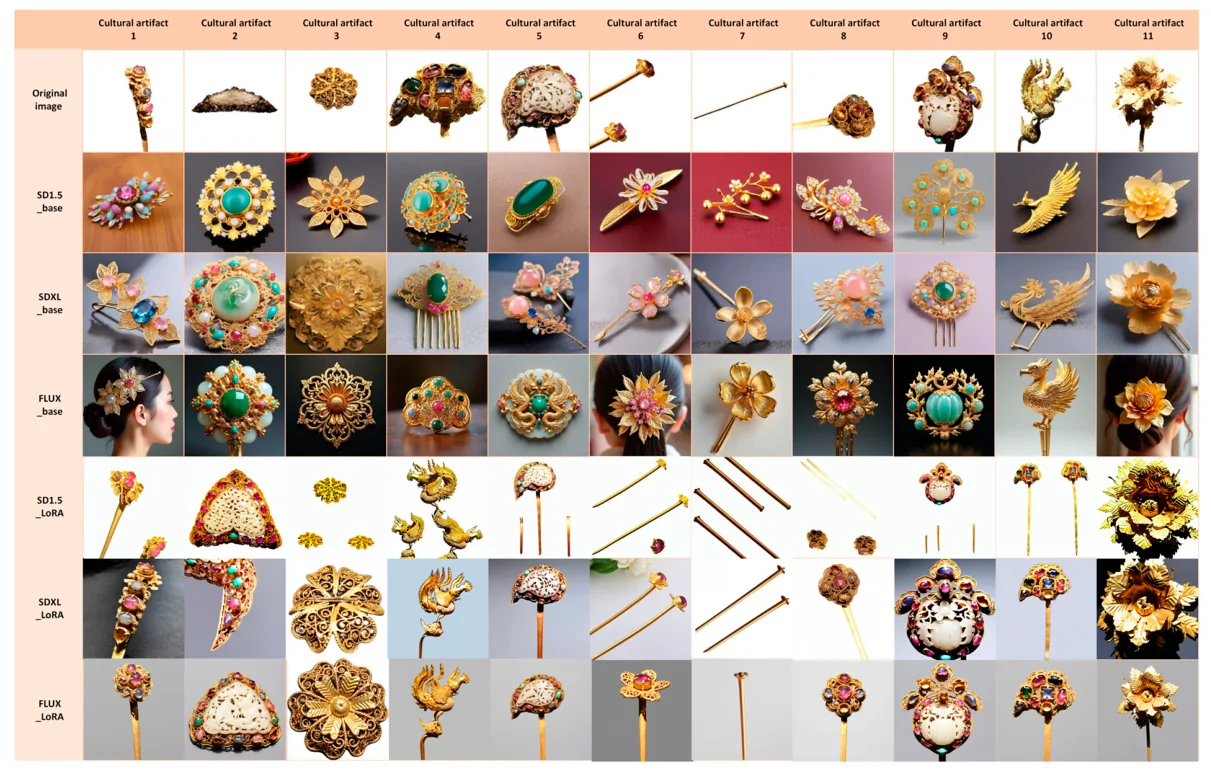
Comparison of generation results from the six models for the headwear category (seed 42).

**Figure 11:**
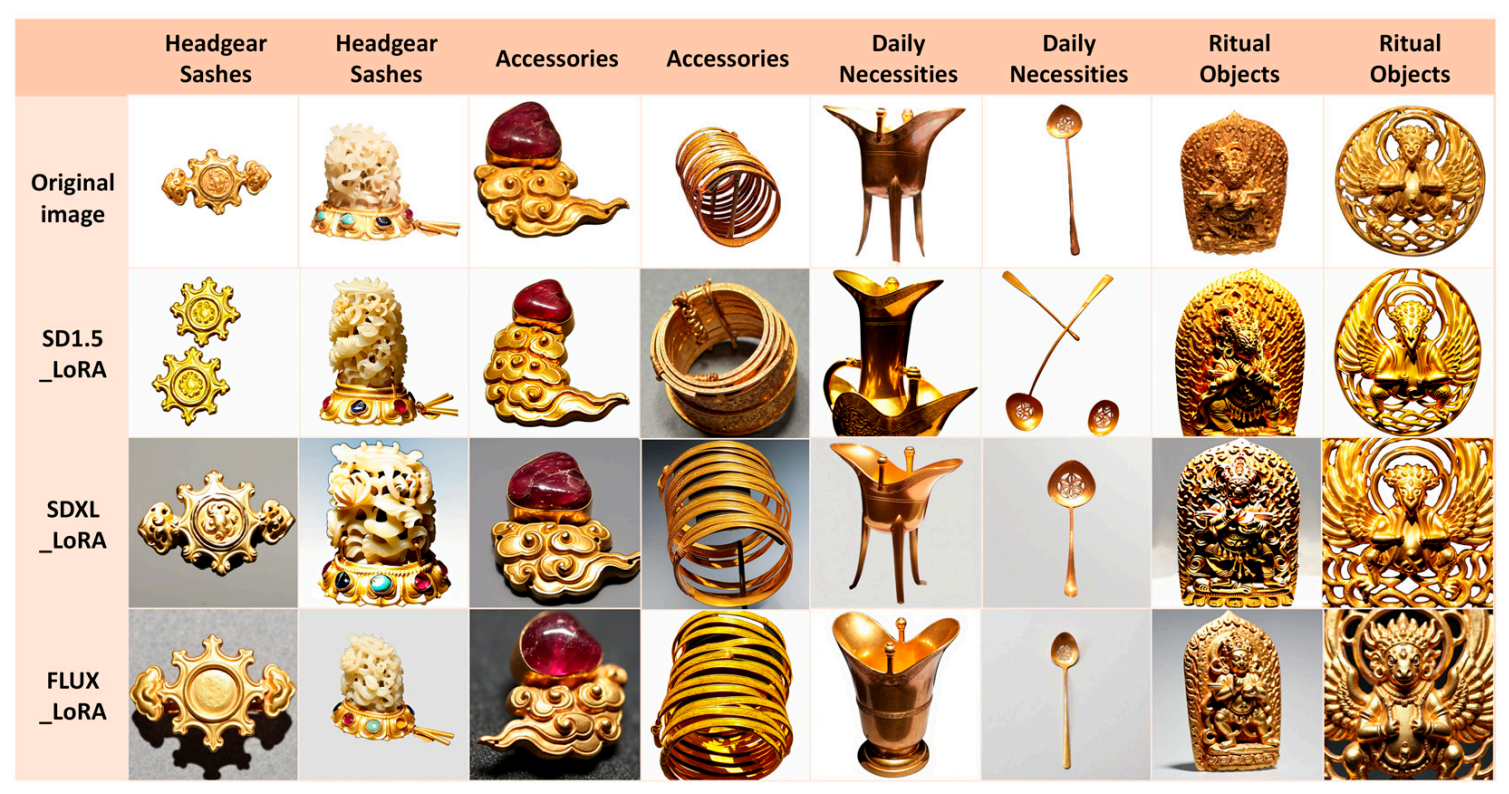
Examples of generated images for the other four categories of gold and silver artifacts (seed 42).

**Figure 12:**
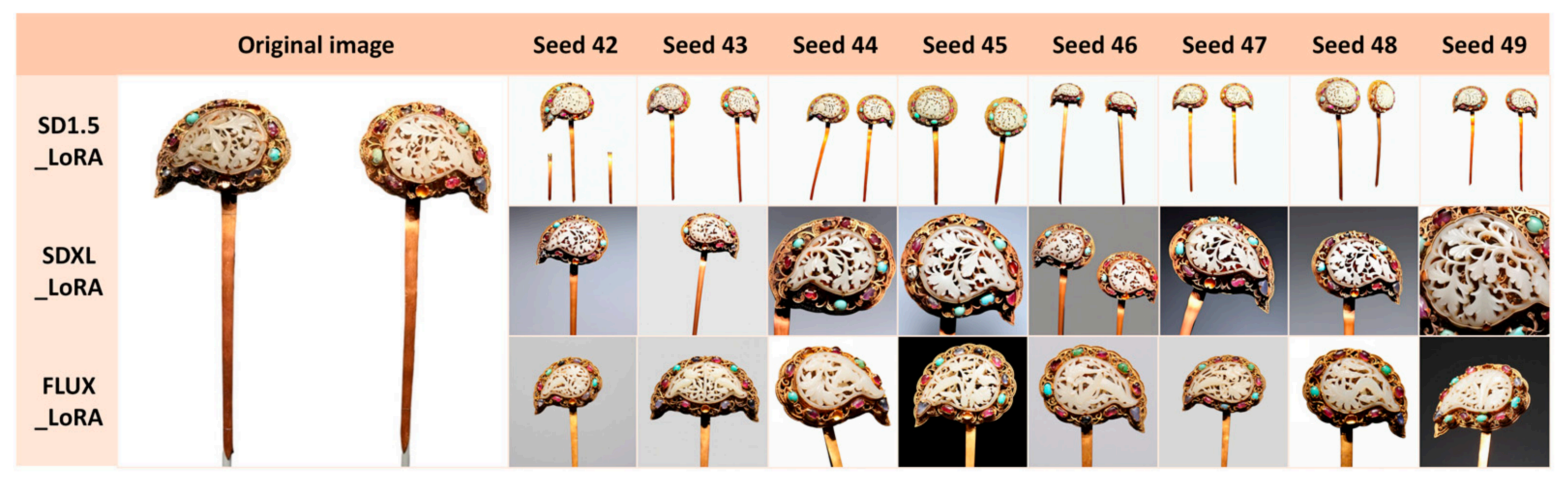
Generation results for the same artifact under different random seeds.

The overall comparison procedure is illustrated in Figure 9, and representative generation results are presented in Figure 10, Figure 11 and Figure 12. Taking the headwear category as an example, Figure 9 illustrates the generation and comparison procedure. The six model configurations, namely SD1.5_base, SD1.5_LoRA, SDXL_base, SDXL_LoRA, FLUX_base, and FLUX_LoRA, are denoted as Images 1–6, respectively. Image 0 represents the corresponding reference images of the target artifacts. Images 1–6 contain the generated images produced by the six model configurations using the same artifact prompts. The generated images are compared to the corresponding reference images to examine differences in form and structure, decorative patterns, material appearance, and color composition. Representative generation results are presented in Figure 10, Figure 11 and Figure 12.

#### 5.2.2. Longitudinal Comparison: Observation of Generation Results Before and After Fine-Tuning

The three base models demonstrated general image generation capabilities before fine-tuning. However, their generated images showed no clear correspondence with the four visual feature dimensions of the gold and silver artifacts from the Tomb of Prince Liangzhuang. After LoRA fine-tuning, all three models generated images that exhibited visual characteristics corresponding to the four dimensions across all five artifact categories. The differences between the base models and their corresponding LoRA-fine-tuned models were readily observable.

LoRA fine-tuning enabled the pre-trained models to successfully learn and generate the four-dimensional visual features of the gold and silver artifacts from the Tomb of the Prince of Liangzhuang. The four-dimensional annotated dataset and LoRA training effectively transferred the visual features of these artifacts to the trained models. As the differences before and after fine-tuning were clearly visible, subsequent quantitative experiments did not repeat the validation of this conclusion but instead focused on a detailed comparison between the three fine-tuned models.

#### 5.2.3. Side-by-Side Comparison: Observations on Performance Differences Among the Three Fine-Tuned Models

After the vertical comparison, the three LoRA-fine-tuned models were further compared using the same artifacts and prompts. The purpose of this comparison was to examine their differences in representing the four visual feature dimensions. The following observations are based on a comprehensive visual comparison across all five artifact categories.

- SD1.5_LoRA

SD1.5_LoRA generally captured the overall form and structure of the artifacts and produced relatively stable results across different cases. However, some generated images exhibited structural distortion or the unintended duplication and accumulation of components. Compared to the other two models, its representation of decorative patterns, material appearance, and color composition was relatively limited. The lines of decorative patterns were generally coarse, and the visual representation of metallic appearance was less pronounced. Fine craftsmanship features, such as filigree structures, were also less clearly represented.

- SDXL_LoRA

SDXL_LoRA showed relatively strong performance in both form and structure and decorative patterns. Its generated images generally maintained clear overall contours and relatively complete decorative structures, resulting in a comparatively balanced performance across the four visual feature dimensions. However, its representation of material appearance was less pronounced than that observed in FLUX_LoRA. Minor deviations in artifact contours were also observed in some cases. Overall, SDXL_LoRA showed relatively strong correspondence with the forms and decorative structures of the reference artifacts.

- FLUX_LoRA

FLUX_LoRA showed relatively strong performance in material appearance and color composition. Across the observed cases, it generally produced more detailed metallic surface characteristics and more pronounced material luster. However, greater variation was observed in the representation of form and structure and decorative patterns. For some artifacts, the generated forms and decorative patterns deviated substantially from those of the corresponding reference artifacts, despite the relatively strong representation of material appearance.

The observed difference may be related to the characteristics of the FLUX architecture and the limited size of the training data. Under the current experimental conditions, the model may have learned some frequently co-occurring visual characteristics in the training images more strongly than the specific structural characteristics of individual artifacts. However, this interpretation is preliminary and cannot be established from the present visual comparison alone. Further experiments controlling for training set size, model capacity, and annotation composition would be required to examine the underlying mechanism.

### 5.3. Quantitative Experiments

The visual observations in Section 5.2 provided a preliminary assessment through visual comparison; however, the observational method is subjective and cannot quantify the degree of difference between models. In this section, we conduct a quantitative comparison of the generation quality of the three fine-tuned models using objective numerical metrics. Images 2, 4, and 6, generated by the three LoRA fine-tuned models (SD1.5_LoRA, SDXL_LoRA, and FLUX_LoRA), are compared quantitatively with images from the reference set.

#### 5.3.1. Experimental Design

- Construction of the Reference Set

Separate reference sets were constructed for the five artifact categories. The reference sets consist of panoramic images of the gold and silver artifacts used as visual references for the subsequent evaluation of generated images. Multiple images were selected for each artifact to provide visual references for the corresponding artifact instances.

The reference images were selected independently of the training images. A category-by-category comparison was conducted to verify that no reference image was included in the corresponding training set. Although the reference and training images were derived from the same collection of artifacts, the image files used for evaluation were distinct from those used for training. Therefore, the quantitative evaluation was not affected by direct image overlap between the training and reference sets.

The reference set covered all five artifact categories and all 82 artifacts. The numbers of reference images included in each category are presented in Table 13.

- Models and Generation Parameters

The models used in the experiment were SD1.5_LoRA, SDXL_LoRA, and FLUX_LoRA. The same generation parameters were used for all three models. The sampler was DPM++ 2M, the number of sampling steps was 30, the classifier-free guidance (CFG) scale was 7.0, the output resolution was 768 × 768 pixels, and the LoRA weight was 0.7.

To evaluate the stability and variation in the generated results across random seeds, each test prompt was used to generate eight images with fixed random seeds from 42 to 49. The same sequence of random seeds was used for all three models to ensure a consistent basis for comparison and reproducibility of the generation process.

#### 5.3.2. Evaluation Metrics

This study employed two types of evaluation metrics: image-wise pairwise metrics and distribution-level metrics. The image-wise pairwise metrics included the Structural Similarity Index Measure (SSIM), Learned Perceptual Image Patch Similarity (LPIPS), and CLIP Score. The distribution-level metrics included Fréchet Inception Distance (FID) and Kernel Inception Distance (KID), which were used primarily as exploratory measures.

- Image-Wise Pairwise Metrics

Structural Similarity Index Measure (SSIM). SSIM measures the structural similarity between a generated image and a corresponding reference image at the image level. Its value ranges from zero to one, with higher values indicating greater structural similarity. Before calculation, the images were uniformly resized to 512 × 512 pixels. SSIM was calculated using the scikit-image library (version 0.25.2).

Learned Perceptual Image Patch Similarity (LPIPS). LPIPS measures perceptual similarity between images based on deep neural network features. Lower values indicate greater perceptual similarity. In this study, AlexNet was used as the feature extraction network, implemented through the lpips library (version 0.1.4) with net = ‘alex’.

CLIP Score measures the semantic alignment between a generated image and its corresponding text prompt. In the implementation used in this study, the score ranges from zero to 100, with higher values indicating stronger alignment between the generated image and the text description. The CLIP Score was calculated using the OpenAI CLIP ViT-B/32 model, loaded via the Hugging Face transformers library (version 4.37.2).

- Distribution-Level Metrics

Fréchet Inception Distance (FID). FID measures the distributional distance between generated images and reference images in the feature space of an Inception network. Lower FID values indicate greater similarity between the two image distributions. In this study, features were extracted using the Inception-v3 model pre-trained on ImageNet, producing a 2048-dimensional feature representation.

Kernel Inception Distance (KID). KID is a distribution-level metric based on a polynomial kernel and is often considered more suitable than FID for relatively small sample sizes. Lower KID values indicate greater similarity between the generated and reference image distributions. The same Inception-v3 feature representations were used for KID as for FID.

For the image-wise pairwise metrics, the calculation was performed hierarchically using the artifact as the independent statistical unit. For each artifact, the metric value of each generated image was first calculated against all reference images belonging to the same artifact, and the mean was taken across the corresponding reference images. The eight generated images produced using random seeds 42–49 were then averaged to obtain one representative metric value for each artifact. Thus, each artifact contributed one representative value for each model and each image-wise pairwise metric.

The resulting artifact-level values were used for pairwise statistical comparisons among the three LoRA-fine-tuned models. This procedure avoids treating the eight generated images or multiple reference image comparisons as independent observations and therefore reduces the risk of pseudo-replication.

The artifact-level results are reported as mean ± standard deviation (SD). The 95% confidence intervals (CIs) were calculated using bootstrap resampling with 1000 repetitions. Pairwise comparisons among the three models were performed using the Wilcoxon signed-rank test, with a nominal significance level of α = 0.05. Because three pairwise comparisons were conducted among the three models, the Bonferroni correction was applied, resulting in an adjusted significance threshold of α′ = 0.0167.

For FID and KID, which are distribution-level metrics rather than image-wise paired metrics, the calculations were performed separately at the distribution level. These two metrics were used primarily for exploratory comparison and were not subjected to the artifact-level paired statistical testing described above.

#### 5.3.3. Statistical Analysis

All image-wise pairwise metrics are reported as mean ± standard deviation (mean ± SD), and 95% confidence intervals (CIs) are calculated using bootstrap resampling with 1000 repetitions. The seed-to-seed standard deviation (seed-to-seed SD) is used to characterize the stability of the generation results across different random seeds. Specifically, for each prompt, the standard deviation of the metric values obtained from the eight generated images using seeds 42–49 is calculated.

Pairwise comparisons among the three models were performed using the Wilcoxon signed-rank test, a nonparametric paired test. The artifact was used as the independent statistical unit. For each artifact, the mean metric value across the eight seed-generated images was calculated to obtain one representative value for that artifact. Therefore, the paired sample size, *n*, corresponds to the number of artifacts in each category (*n* = 11–28), rather than the total number of generated images.

Because three pairwise comparisons were conducted among the three models, the significance level was adjusted using the Bonferroni correction: α = 0.05/3 ≈ 0.0167

For the distribution-level metrics FID and KID, only point estimates and standard errors (SEs) are reported because of the limited size of the reference set. No statistical inference was performed for these two metrics.

#### 5.3.4. Experimental Results

1. Results by Category

The results of the image-wise pairwise metrics for the five artifact categories are presented in Table 14, Table 15, Table 16, Table 17 and Table 18.

2. Results of Statistical Tests

The results of the Wilcoxon signed-rank tests and effect sizes are summarized in Table 19. For SSIM, all 10 comparisons involving SD1.5_LoRA against FLUX_LoRA and SDXL_LoRA across the five artifact categories were statistically significant (*p* < 0.0167). For LPIPS, all five comparisons between SD1.5_LoRA and SDXL_LoRA were statistically significant, while four of the five comparisons between SD1.5_LoRA and FLUX_LoRA were statistically significant. The comparison for the ritual objects category did not remain statistically significant after Bonferroni correction (*p* = 0.054).

3. Equally Weighted Average across Categories

To provide an overall summary while avoiding the results being dominated by categories with larger sample sizes, an equally weighted averaging procedure was applied. First, the mean metric value for each model was calculated within each artifact category. The five category-level means were then averaged with equal weights. The results are presented in Table 20.

The equally weighted results show that SD1.5_LoRA achieved the highest mean SSIM (0.627) and the lowest mean LPIPS (0.506) across the five categories. It also showed the lowest mean seed-to-seed standard deviation (0.050), indicating relatively low variation across random seeds under the evaluated conditions. FLUX_LoRA and SDXL_LoRA obtained slightly higher mean CLIP Scores, although the differences between the models were relatively small.

4. Distribution-Level Distance Metrics

FID and KID were used as distribution-level distance metrics. Their estimates can be sensitive to the number and composition of samples used to characterize the underlying image distributions. In this study, the reference set comprised 1004 images representing 82 artifacts, while 1968 generated images were produced across the three models and eight random seeds. Across the five artifact categories, the FID values ranged from 124.63 to 186.48. No consistent ordering of the models was observed across categories. Given the limited number of reference images available for each category and the sensitivity of distribution-level metrics to sample composition, the FID and KID results are treated as exploratory evidence rather than as the primary basis for comparing model performance. The FID and KID results for the five artifact categories are presented in Table 21.

#### 5.3.5. Analysis of Results

Based on the quantitative results across the five artifact categories, the three models exhibited differentiated performance characteristics.

First, SD1.5_LoRA showed consistently higher structural similarity and lower seed-to-seed variation across the five categories. SD1.5_LoRA showed higher SSIM values, lower LPIPS values, and lower seed-to-seed standard deviations than the other two models across all five categories, with SSIM values ranging from 0.558 to 0.679, LPIPS values ranging from 0.447 to 0.552, and seed-to-seed SD values ranging from 0.040 to 0.063. All 10 pairwise comparisons involving SD1.5_LoRA and the other two models for SSIM were statistically significant at the Bonferroni-adjusted threshold (*p* < 0.0167), and most of the corresponding effect sizes were large (|r| > 0.5). In the equally weighted averages across categories, SD1.5_LoRA achieved an SSIM of 0.627, which was approximately 25% higher than that of FLUX_LoRA (0.503) and 33% higher than that of SDXL_LoRA (0.470). Its LPIPS value of 0.506 was approximately 14% lower than that of FLUX_LoRA and 23% lower than that of SDXL_LoRA. These results indicate that, under the present LoRA fine-tuning conditions and with a relatively small number of artifacts per category (11–28), SD1.5_LoRA showed higher structural similarity and lower variation across random seeds. The mechanisms underlying these differences, including possible effects of model architecture, text encoders, and training configurations, require further investigation through controlled ablation studies.

Second, CLIP Score showed relatively limited discrimination among the three models at the cross-category level, while category-dependent differences were observed. The CLIP Score did not yield consistent model rankings across all five categories: in the “Accessories” category, there were no significant differences among the three models; in the “Daily Necessities” category, FLUX and SDXL scored significantly higher than SD1.5 (*p* = 0.003, 0.003); in the “Headgear and Sashes” category, both FLUX and SD1.5 scored significantly higher than SDXL (*p* = 0.003, 0.003), while no significant difference was found between FLUX and SD1.5 (*p* = 0.623); in the “Headwear” category, FLUX scored significantly higher than the other two (*p* = 0.014, 0.007); and in the “Ritual Objects” category, FLUX scored significantly higher than the other two (*p* = 0.014, 0.014). In the equally weighted averages, the difference between the highest and lowest mean CLIP Scores was only 1.24 points (31.21, 29.97, and 30.24), and the corresponding confidence intervals showed substantial overlap. These results suggest that the three models had broadly comparable semantic alignment with the text prompts derived from the four-dimensional annotation framework at the cross-category level, although category-specific differences were evident. Therefore, CLIP Score should be interpreted together with category-specific results rather than used as a single basis for cross-category model comparison.

Third, FID showed limited discriminative value under the present evaluation conditions, while KID was treated as an exploratory metric. The FID values across the five categories ranged from 124.63 to 186.48. For a given model, the variation in FID across categories, such as the range from 124.63 to 157.85 for SD1.5_LoRA, was in some cases larger than the differences among models within the same category, and no consistent ordering of the models was observed across categories. This pattern highlights the sensitivity of FID to the composition and size of the reference sample under the present evaluation conditions. Because each category contained approximately 200 reference images and the reference images represented a relatively limited number of artifact instances, the FID results were considered insufficiently robust for use as the primary basis for model comparison. The standard errors of KID were also relatively large, with the SE for some model–category combinations corresponding to approximately 10–18% of the point estimate. SD1.5_LoRA showed the lowest KID in four of the five categories, with the exception of the Headgear and Sashes category. This direction was broadly consistent with the SSIM and LPIPS results; however, given the limited reference sample size and the exploratory role of these distribution-level metrics, the KID results were not used as a primary criterion for model comparison.

Fourth, the quantitative results indicate that the three models exhibited different performance characteristics across evaluation dimensions. SD1.5_LoRA showed higher structural similarity and lower seed-to-seed variation under the present experimental conditions, whereas FLUX_LoRA and SDXL_LoRA showed relatively higher CLIP Scores in several category-specific comparisons. Differences in material appearance observed in the visual analysis were also not fully captured by the quantitative metrics. These results indicate that the choice of model may depend on the specific evaluation dimension and application requirement rather than on a single overall metric. In addition, the Headwear category showed a partial divergence between structural similarity and semantic alignment: SD1.5_LoRA achieved relatively high structural metric values in this category but did not show a correspondingly high CLIP Score. One possible explanation is that the Headwear category contains relatively complex structural relationships and densely described visual attributes. However, this interpretation remains tentative and requires further investigation using controlled experiments.

The quantitative results provide an objective reference for the subsequent subjective evaluation. SSIM and LPIPS primarily measure image-level structural and perceptual similarity and may favor generated outputs that retain relatively conservative visual structures. Human evaluation, in contrast, can capture perceptual aspects that are not fully represented by these metrics, including the legibility of decorative patterns, material realism, and overall visual coherence. Therefore, Section 5.4 further evaluates the three models from multiple perceptual dimensions through subjective evaluation.

### 5.4. Subjective Evaluation Experiment

The quantitative experiments compared the generation results across different artifact categories and models using objective image-based metrics. However, objective metrics cannot fully capture users’ perceptual preferences for the generated images, particularly in terms of esthetic and visual perception. Therefore, this section evaluates the performance of the three models across the four visual feature dimensions from the user’s perspective through a questionnaire-based subjective evaluation.

#### 5.4.1. Experimental Setup

One questionnaire was designed for each artifact category. Approximately 20 participants were recruited for each questionnaire. Across the five questionnaires, the total number of questionnaire participation instances was 101, with one category receiving one additional participation instance. The participants included individuals with design backgrounds and members of the general public, providing diversity in evaluation perspectives.

To avoid excessive questionnaire length and its potential effect on response quality, approximately 50% of the artifacts in each category were randomly selected for evaluation. The selected artifacts comprised six Headwear artifacts, 11 Headgear and Sashes artifacts, six Accessories artifacts, 13 Daily Necessities artifacts, and five Ritual Objects artifacts, resulting in 41 evaluated artifacts in total. Each evaluated artifact was assessed by approximately 20 participants within its corresponding category, resulting in approximately 20 artifact-level evaluations per artifact.

The questionnaire used a matrix-style single-choice format. For each test artifact, four image groups were presented: a photograph of the actual artifact, a collage of eight generated images from Model A, a collage of eight generated images from Model B, and a collage of eight generated images from Model C. The generated images from the three models were randomly assigned to labels A, B, and C within each questionnaire, and participants were not informed of the correspondence between the labels and the underlying models. This design was intended to reduce potential bias arising from prior familiarity with or expectations about a particular model.

Participants were asked to select the model that performed best in each of five evaluation dimensions: (1) Form and Structure, referring to the degree to which the overall outline and structural proportions of the artifact were reproduced; (2) Decorative Patterns, referring to the clarity and accuracy of surface decorations; (3) Material Appearance, referring to the realism of gold and silver appearance and gemstone luster; (4) Color Composition, referring to the overall harmony of the color scheme; and (5) Overall Effect, referring to the overall perceived esthetic quality. For each question, the response options were the three generated images labeled A, B, and C, as well as “All Are Good” and “None Are Good”.

The scoring rules were defined as follows. When Image A, B, or C was selected, the corresponding model received one vote. When “All Are Good” was selected, each of the three models received one vote. When “None Are Good” was selected, none of the three models received a vote.

For each artifact category, the vote percentage of a model was calculated using the total number of artifact-level responses within that category as the denominator:

Vote percentage = Number of votes received by the model/(Number of participants × Number of evaluated artifacts in the category) × 100%.

Thus, the denominator was calculated separately for each category according to the actual number of participants and evaluated artifacts in that category. “All Are Good” and “None Are Good” were retained as distinct response options in the descriptive vote analysis.

For the Friedman test and Kendall’s W analysis, the responses were aggregated at the participant level rather than treating individual artifact-level responses as independent observations. Each participant’s evaluations across the artifacts assigned to that participant were aggregated to obtain model-level scores, which were then converted into ranks for the subsequent analyses.

Two statistical methods were employed.

- Friedman test. The Friedman test was used to examine whether statistically significant differences existed among the three models in the participant-level subjective evaluation scores. As a nonparametric test for related samples, the Friedman test does not require the data to follow a normal distribution.
- Kendall’s coefficient of concordance (Kendall’s W). Kendall’s W was used to assess inter-rater agreement among participants. Its value ranges from zero to one, with larger values indicating greater agreement among raters. For the calculation, each participant’s model-level scores were converted into a ranking matrix. The model receiving the highest score was assigned the highest rank, while models receiving equal scores were assigned tied ranks. Responses in which all three models received the same score, such as “All Are Good” or “None Are Good,” were treated as non-discriminative responses. Kendall’s W with tie correction was then calculated. Both the W value based on the full sample and the W value calculated after excluding non-discriminative responses were reported to provide a more complete assessment of inter-rater agreement.

#### 5.4.2. Experimental Results by Category

The subjective evaluation results for the five artifact categories are presented in Table 22. The Friedman test was statistically significant for all five categories (*p* < 0.001), indicating that the subjective evaluation rankings of the three models differed significantly within each category.

The vote percentages for the three models across the five evaluation dimensions and the five artifact categories are presented in Table 23.

The dimension-specific results showed differentiated patterns across the five artifact categories. SDXL_LoRA received the highest vote percentage in Form and Structure, Decorative Patterns, and Overall Effect across all five categories, except for Overall Effect in the Accessories category and Sashes categories. Its advantage was particularly pronounced for Decorative Patterns, with an equally weighted average vote percentage of 60.8% and a value of 80.0% in the Headwear category. This indicates that SDXL_LoRA received relatively high recognition from participants for the clarity and accuracy of the generated decorative patterns.

FLUX_LoRA received the highest vote percentage in Material Appearance across all five categories and in Color Composition overall (equally weighted average 41.9% Its equally weighted average vote percentage for Material Appearance was 60.0%, reaching 75.9% in the Headgear and Sashes category. This result indicates that participants more frequently selected FLUX_LoRA when evaluating the appearance of gold and silver materials and gemstone luster.

SD1.5_LoRA showed relatively low vote percentages across most evaluation dimensions, with equally weighted average values ranging from 2.9% to 21.4%. Relatively higher vote percentages were observed for Form and Structure in the Headwear and Ritual Objects categories, where SD1.5_LoRA received 33.3% and 29.5% of the votes, respectively.

Notably, FLUX_LoRA received the highest vote percentages for Overall Effect in the Accessories and Headgear and Sashes categories, at 32.5% and 51.8%, respectively. This differed from the pattern observed in the other three categories, where SDXL_LoRA received the highest vote percentage for Overall Effect. One possible explanation is that the Accessories and Headgear and Sashes categories contain relatively complex decorative features, such that the stronger performance of FLUX_LoRA in Material Appearance and Color Composition may have contributed more strongly to participants’ overall visual evaluations. This interpretation remains tentative and requires further investigation.

Regarding inter-rater agreement, the Kendall’s W values for each dimension and category are presented in Table 24 and Table 25. For the full sample, including the “All Are Good” and “None Are Good” responses, the equally weighted mean W values across the five categories ranged from 0.155 to 0.336. After excluding non-discriminative responses, the corresponding W values increased to 0.209–0.396. This increase indicates that responses in which participants did not distinguish among the three models contributed to lower observed inter-rater agreement. The relatively low-to-moderate W values also indicate that participants’ preferences were not completely consistent across all evaluation dimensions and artifact categories.

Overall inter-rater agreement was relatively low. Three factors may have contributed to this result. First, esthetic evaluation is inherently subjective, and participants from different backgrounds may apply different criteria when judging dimensions such as Material Appearance and Color Composition. Second, the questionnaire adopted a single-choice “select the best” format rather than a complete ranking of the three models (first, second, and third). The two unselected models were therefore treated as tied, which limited the degree of rank differentiation available for calculating Kendall’s W. Third, the “All Are Good” and “None Are Good” options occurred relatively frequently in some dimensions, further reducing the discriminative information in the full-sample responses and consequently lowering the observed W values.

It is noteworthy that relatively high W values were observed in some dimensions where relatively large differences in vote percentages were found among the three models, whereas lower W values tended to occur in dimensions with smaller differences or greater dispersion in participants’ preferences. This distribution provides useful information for interpreting the subjective evaluation results. Specifically, the W values for Decorative Patterns and Overall Effect in the Headwear category were relatively high (W = 0.652 and 0.599, respectively), as was the W value for Material Appearance in the Headgear and Sashes category (W = 0.534). These dimensions also showed relatively clear differences in vote percentages among the three models. For example, SDXL_LoRA received 78.3% of the votes for Decorative Patterns in the Headwear category (Table 23).

In contrast, Form and Structure had the lowest equally weighted mean W value across the five categories (W = 0.155), indicating greater dispersion in participants’ preferences for this dimension. However, this result should not be interpreted simply as evidence that the three models had similar generation quality in Form and Structure. Low inter-rater agreement may also result from dispersed individual preferences, the single-choice response format, and the presence of non-discriminative responses.

The Accessories category had the highest exclusion rate, ranging from 38.3% to 47.5% across the evaluation dimensions. This indicates that a relatively large proportion of responses in this category selected either “All Are Good” or “None Are Good,” thereby providing limited discrimination among the three models. The relatively high frequency of non-discriminative responses may partly explain the lower W values observed in the full-sample analysis for this category. After excluding these responses, the W values increased across all evaluation dimensions, indicating greater inter-rater agreement among participants who expressed a clear preference for one of the three models.

Therefore, Kendall’s W should be interpreted as a reference measure of inter-rater agreement rather than as an absolute indicator of model performance in this study. Its magnitude reflects the degree to which participants’ preferences were consistent and distinguishable among the three models. It should not be interpreted as a direct indicator of data quality.

For the Headwear category (20 participants and six artifacts), SDXL_LoRA received the highest vote percentages for Decorative Patterns (80.0%) and Overall Effect (78.3%), while FLUX_LoRA received the highest vote percentage for Material Appearance (55.8%). SD1.5_LoRA received 33.3% of the votes for Form and Structure. In the Color Composition dimension, the “All Are Good” option was selected 24 times, indicating that a proportion of participants considered the color composition of the generated images from all three models acceptable for the evaluated artifacts.

For Headgear and Sashes (20 participants and 11 artifacts), FLUX_LoRA received the highest vote percentages for Material Appearance (75.9%), Color Composition (68.2%), and Overall Effect (51.8%). The “None Are Good” option occurred relatively frequently in this category, with 24 selections for Overall Effect and 26 for Decorative Patterns. These responses were mainly associated with artifacts featuring complex crown-top structures. This result suggests that the three models encountered difficulties in reproducing certain multi-layered three-dimensional structures. The difficulty may be related to the structural complexity of these artifacts, which involve multiple craft features, including openwork and inlay.

For Accessories (20 participants and six artifacts), FLUX_LoRA received slightly higher vote percentages than SDXL_LoRA for Material Appearance (51.7%) and Overall Effect (32.5%), although the difference between the two models was relatively small for Overall Effect. The “All Are Good” option occurred most frequently in this category, with 57 selections for Color Composition and 48 for Form and Structure. This indicates that a relatively large proportion of participants considered the outputs of all three models acceptable in these dimensions, resulting in less differentiation among the models in some aspects of the subjective evaluation.

For Daily Necessities (20 participants and 13 artifacts), SDXL_LoRA received the highest vote percentages for Decorative Patterns (69.6%) and Overall Effect (72.3%), while FLUX_LoRA received the highest vote percentage for Material Appearance (51.9%). This category included the largest number of evaluated artifacts (13), providing a relatively larger artifact sample than the other categories. Nevertheless, the category-level results should still be interpreted in the context of the limited number of evaluated artifacts.

For Ritual Objects (21 participants and five artifacts), SDXL_LoRA received the highest vote percentage for Overall Effect (66.7%), while FLUX_LoRA received the highest vote percentage for Material Appearance (64.8%). This category included the smallest number of evaluated artifacts (five), and therefore the corresponding category-level results should be interpreted with greater caution.

#### 5.4.3. Summary Analysis of the Five Categories

To reduce the influence of differences in sample size across artifact categories on the overall summary, an equal-weight averaging method was employed. First, the vote percentage for each model was calculated separately within each category. The five category-level percentages were then averaged with equal weights, so that categories with a larger number of evaluated artifacts, such as Daily Necessities with 13 artifacts, would not disproportionately influence the overall results. The equally weighted results across the five artifact categories are presented in Table 26.

The summary results showed that SDXL_LoRA received the highest equally weighted vote percentage for Overall Effect (55.4%). FLUX_LoRA received the highest vote percentages for Material Appearance (60.0%) and Color Composition (41.9%), whereas SD1.5_LoRA received relatively low vote percentages across the evaluation dimensions. The three models therefore showed differentiated patterns across the evaluated dimensions. SDXL_LoRA received relatively high recognition for Decorative Patterns and Overall Effect, FLUX_LoRA was more frequently selected for Material Appearance and Color Composition, and SD1.5_LoRA received relatively higher recognition for Form and Structure in several category-specific comparisons.

Differences were also observed across artifact categories. SDXL_LoRA received the highest vote percentage for Overall Effect in Headwear, Daily Necessities, and Ritual Objects, whereas FLUX_LoRA received the highest vote percentage in Headgear and Sashes and Accessories. This category-dependent pattern indicates that participants’ subjective preferences varied across artifact categories. In particular, the higher vote percentages received by FLUX_LoRA for Material Appearance and Color Composition may have contributed to its relatively higher Overall Effect preference in Headgear and Sashes and Accessories, whereas the higher vote percentages received by SDXL_LoRA for Decorative Patterns may have contributed to its relatively higher Overall Effect preference in Headwear, Daily Necessities, and Ritual Objects. These interpretations are descriptive and should not be regarded as evidence of a causal relationship between artifact category characteristics and model preference.

#### 5.4.4. Analysis of Consistency Between Objective and Subjective Evaluations

This study further examined the relationship between objective quantitative metrics and subjective evaluation results. The quantitative and subjective evaluations showed different patterns in model rankings. In the quantitative evaluation, SD1.5_LoRA showed relatively high structural similarity according to SSIM and LPIPS, whereas SDXL_LoRA received the highest equally weighted vote percentage for Overall Effect in the subjective evaluation. To examine the relationship between these two types of evaluation, Spearman’s rank correlation coefficients were calculated between the objective metric values and the Overall Effect vote percentages using 15 observations, corresponding to five artifact categories and three models.

The results are presented in Table 27. The correlations showed different directions across the objective metrics. SSIM exhibited a negative descriptive association with the Overall Effect vote percentage (ρ = −0.466, *p* = 0.080), whereas LPIPS showed a positive association (ρ = 0.596, *p* = 0.019). The objective composite score also showed a negative association with the Overall Effect vote percentage (ρ = −0.564, *p* = 0.028). These results indicate that higher values of the objective structural metrics did not necessarily correspond to higher subjective preference in the present evaluation. In particular, SD1.5_LoRA, which showed relatively high structural similarity in the quantitative evaluation, did not receive the highest Overall Effect vote percentage, whereas SDXL_LoRA and FLUX_LoRA received higher subjective preference in the evaluated categories.

However, these correlation coefficients should be interpreted cautiously. The analysis was based on only 15 observations, and the three model observations within the same artifact category are related and may not be statistically independent. In addition, the objective metrics and subjective vote percentages capture different aspects of generation quality. SSIM and LPIPS primarily characterize image-level structural and perceptual similarity to the reference images, whereas the subjective evaluation incorporates participants’ judgments of Form and Structure, Decorative Patterns, Material Appearance, Color Composition, and Overall Effect. Therefore, the observed correlations should be regarded as descriptive evidence of differences in ranking patterns within the present dataset rather than as evidence of a general relationship between structural similarity and subjective preference. In particular, the results do not support the interpretation that higher structural similarity generally leads to lower subjective evaluation.

To further examine the influence of the relative weights assigned to subjective and objective evaluations on the comprehensive evaluation results, a weighted composite score was constructed as follows:

Comprehensive Score = w × Normalized Subjective Score + (1 − w) × Objective Composite Score,

where w represents the weight assigned to the subjective evaluation. The sensitivity analysis results are presented in Table 28.

When w = 0.3, corresponding to a greater weight assigned to the objective evaluation, SD1.5_LoRA obtained the highest composite score in all five artifact categories. When w = 0.5, corresponding to equal weighting of the subjective and objective evaluations, SDXL_LoRA obtained the highest composite score in Headwear, Daily Necessities, and Ritual Objects, whereas FLUX_LoRA obtained the highest composite score in Headgear and Sashes and Accessories. When w = 0.7, corresponding to a greater weight assigned to the subjective evaluation, the distribution of the highest-scoring models was the same as that observed at w = 0.5.

Within the three tested weighting schemes, the category-level distribution of the highest-scoring models remained unchanged between w = 0.5 and w = 0.7. No single model obtained the highest composite score across all five artifact categories and all three tested weighting schemes. These results indicate that the model selection outcome depended on the relative emphasis placed on objective and subjective evaluation. Therefore, rather than asserting the absolute superiority or inferiority of a single model, this study reports the evaluation results by visual dimension and artifact category.

The directional differences between the objective and subjective evaluations may be related to differences in the aspects captured by the two evaluation approaches. SSIM and LPIPS quantify image similarity at the pixel or deep-feature level, respectively, and therefore primarily characterize the degree of similarity between generated images and reference images. In contrast, human esthetic evaluation does not depend solely on similarity to a reference image, but also considers factors such as the clarity of decorative patterns, the appearance of materials, color composition, and overall visual effect. In the subjective evaluation, FLUX_LoRA received the highest equally weighted vote percentages for Material Appearance (60.0%) and Color Composition (41.9%), whereas SDXL_LoRA received the highest vote percentages for Form and Structure (47.1%), Decorative Patterns (60.8%), and Overall Effect (55.4%). SD1.5_LoRA received a relatively low equally weighted vote percentage for Form and Structure (21.4%) and lower vote percentages in the other dimensions. These results indicate that the model with relatively high image-level structural similarity does not necessarily receive the highest subjective preference. In the present evaluation, SD1.5_LoRA showed relatively high structural similarity in the objective metrics, whereas SDXL_LoRA and FLUX_LoRA received higher subjective preference in several perceptual dimensions. This difference suggests that image similarity metrics and human evaluation capture partially different aspects of generation quality.

It is also noteworthy that the CLIP Score showed category-dependent differences in model rankings. Statistically significant within-category pairwise differences were observed in Headwear, Daily Necessities, Headgear and Sashes, and Ritual Objects, whereas no statistically significant pairwise differences were observed among the three models in Accessories (see Table 19). However, no single model showed a consistent advantage across all five categories. These results suggest that the three models showed broadly comparable text–image semantic alignment under the evaluated conditions, while category-dependent differences were still present. Therefore, the differences between the objective and subjective evaluation results cannot be attributed simply to large differences in semantic alignment. Instead, they may partly reflect the different aspects emphasized by image-based metrics and human perceptual evaluation.

Kendall’s W results provide additional information for interpreting the subjective evaluation. Relatively high inter-rater agreement was observed for dimensions in which the differences in vote percentages among the models were more pronounced, such as Decorative Patterns in Headwear (W = 0.652) and Material Appearance in Headgear and Sashes (W = 0.534). In contrast, lower W values were observed in dimensions with greater dispersion in participants’ preferences, such as Form and Structure (equally weighted mean W = 0.155). These results indicate that the degree of agreement among participants varied across evaluation dimensions and artifact categories. Kendall’s W therefore provides complementary information about the consistency of participants’ judgments, rather than serving as a direct measure of the validity or reliability of the subjective evaluation itself.

Overall, the objective metrics and subjective evaluation should not be regarded as competing evaluation approaches. Rather, they capture different aspects of model performance. The quantitative metrics primarily characterize image-level structural and perceptual similarity and, together with seed-to-seed variation, provide an objective basis for assessing structural fidelity and generation stability. The subjective evaluation incorporates human judgments of visual features such as decorative patterns, material appearance, color composition, and overall visual effect. Therefore, model evaluation should consider the specific requirements of the intended application. For applications emphasizing the preservation or reconstruction of artifact structure, greater weight may be assigned to structural similarity and related objective metrics. For applications emphasizing creative design and visual presentation, subjective evaluation of perceptual quality and esthetic coherence may be given greater consideration.

### 5.5. Resolution Validation Experiments

**Figure 13:**
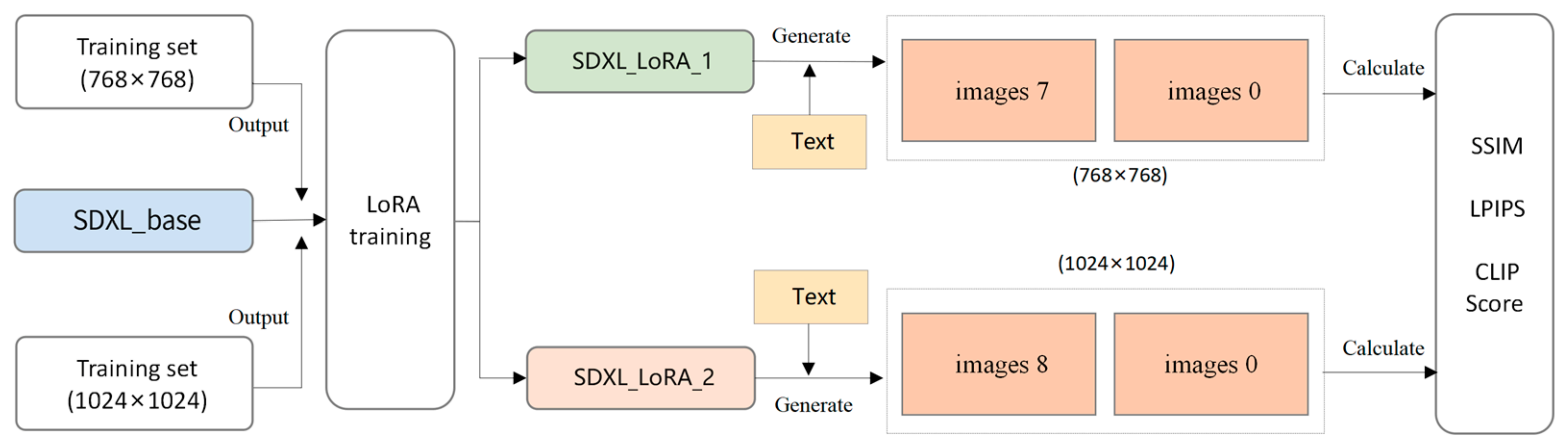
Workflow of the resolution validation experiments.

Generation resolution is an important parameter that may affect the quality and characteristics of diffusion model outputs. To examine the effect of output resolution on the generation of gold and silver artifacts, a resolution comparison experiment was conducted using SDXL_LoRA. The experimental procedure is illustrated in Figure 13.

#### 5.5.1. Experimental Design of the Resolution Validation Experiments

The experiment was conducted using the Headwear reference set, which comprised 11 distinct artifacts. For each artifact, images were generated at two output resolutions, 768 × 768 and 1024 × 1024 pixels, while all other generation parameters were kept identical. These parameters included an LoRA weight of 0.7, eight fixed random seeds (42–49), the DPM++ 2M sampler, 30 sampling steps, and a classifier-free guidance (CFG) scale of 7.0.

For each resolution, 88 images were generated (11 artifacts × eight random seeds), resulting in a total of 176 generated images. The same prompts and random seeds were used at both resolutions to provide a consistent basis for comparison.

The evaluation metrics were consistent with those described in Section 5.3 and included Structural Similarity Index Measure (SSIM), Learned Perceptual Image Patch Similarity (LPIPS) using the AlexNet feature network, CLIP Score based on OpenAI CLIP ViT-B/32, and seed-to-seed standard deviation. SSIM and LPIPS were used to assess image-level structural and perceptual similarity, respectively, while CLIP Score was used to assess image–text semantic alignment. Seed-to-seed standard deviation was calculated from the metric values obtained from the eight fixed random seeds for each artifact and was used to characterize the variation in the generated results across random seeds.

The Wilcoxon signed-rank test was used to compare the metric values between the two resolutions, with each artifact treated as one statistical unit (*n* = 11). For each artifact, the metric values obtained from the eight random seeds were first aggregated to obtain an artifact-level representative value, which was then used for the paired comparison between the two resolutions.

#### 5.5.2. Experimental Results of the Resolution Validation Experiments

**Figure 14:**
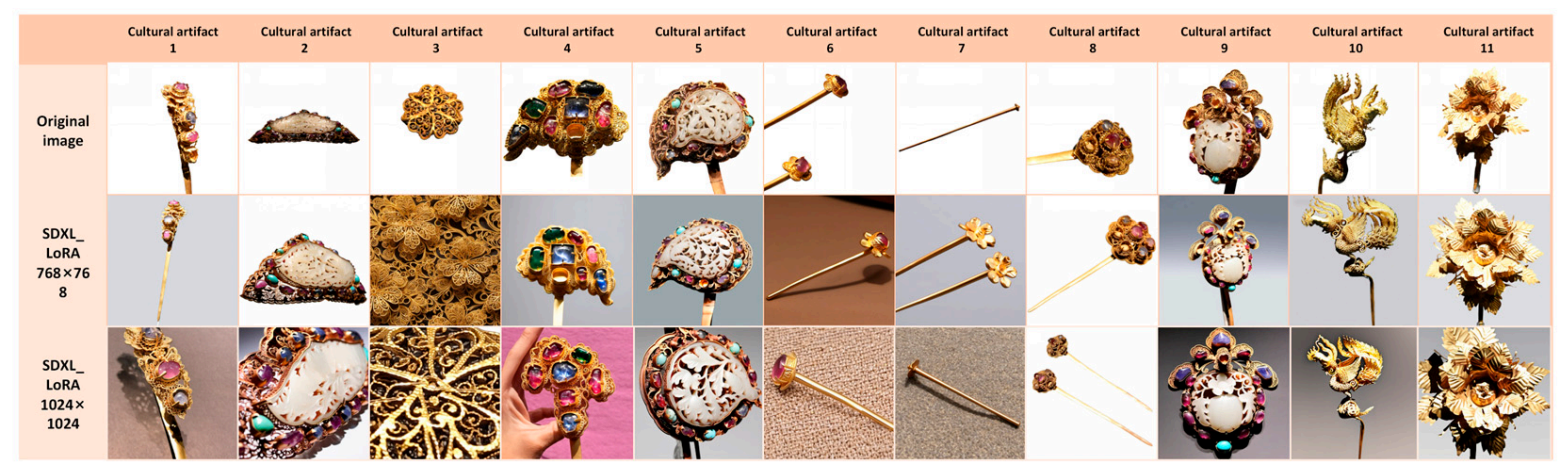
Comparison of generation results at different resolutions.

The objective evaluation results for the two resolutions are presented in Table 29. Representative generated images at the two resolutions are shown in Figure 14.

The 768 × 768 resolution showed higher SSIM (0.523 vs. 0.488) and lower LPIPS (0.614 vs. 0.664) than 1024 × 1024, while 1024 × 1024 showed a slightly higher CLIP Score (30.63 vs. 30.11). However, the differences in all three metrics were not statistically significant (*p* > 0.05). The seed-to-seed standard deviation was also slightly lower at 768 × 768 than at 1024 × 1024 (0.152 vs. 0.162), indicating slightly lower variation across random seeds. Overall, no statistically significant differences were observed between the two resolutions under the evaluated conditions.

#### 5.5.3. Discussion

The results showed that increasing the resolution from 768 × 768 to 1024 × 1024 did not produce a statistically significant improvement in generation quality for the Headwear category. This may be related to the resolution of the training data and the characteristics of the model. Since the training images were preprocessed at 768 × 768, increasing the output resolution may provide limited additional benefit when the available training data contain limited high-frequency detail.

It should be noted that this experiment was conducted using SDXL_LoRA and the Headwear category only, and the generalizability of the results requires further validation with other models and artifact categories. Considering the comparable generation results and computational cost, 768 × 768 was adopted as the unified generation resolution in this study.

### 5.6. Statement on Ethics and Risks of AI-Generated Images

All images generated in this study using diffusion models are AI-generated content and should not be treated as archeological evidence or as evidence of historical facts. Details such as artifact form, ornamentation, and craftsmanship are learned from the training data and synthesized by the models, and may contain structural deviations, ornamentation errors, or inaccurate gemstone settings. Any academic citation or dissemination of the generated images should clearly identify them as AI-generated content and indicate that they are intended for creative and methodological exploration only. They should not be used directly as evidence for archeological conclusions or as a basis for cultural heritage restoration.

AI-generated cultural heritage content requires appropriate review by experts in relevant fields, including archeology, cultural heritage conservation, and the history of arts and crafts, before being used for purposes that require historical or cultural authenticity. In particular, AI-generated images should not be presented as authentic archeological documentation without expert verification. The conclusions of this study are limited to the evaluation of the generative capabilities of the models and do not constitute historical verification of the artifacts themselves.

To ensure reproducibility and traceability, this study records the base model versions, LoRA training parameters, image generation parameters (sampler, sampling steps, CFG scale, resolution, and random seeds), and the complete prompts used for artifact generation. The relevant parameters are provided in Section 5.3.1 and Appendix A. These records allow other researchers to reproduce the generation procedure under the same experimental conditions.

All images generated in this study should be clearly labeled as “AI-generated” when used or disseminated. They should also be visually distinguished from photographs of actual cultural artifacts to reduce the risk of misidentification as authentic archeological records.

The use of diffusion models for cultural heritage image generation also involves potential risks, including the generation of misleading artifact representations, cultural appropriation, and public misunderstanding of historical or cultural information. In this study, the generated images are used solely for evaluating model capabilities in an academic research context. Future applications in areas such as digital restoration or cultural and creative design should involve appropriate review by cultural heritage authorities and relevant domain experts, particularly when historical authenticity or public dissemination is involved.

## 6. Exploration of AIGC Animation Applications

AIGC (AI-generated content) refers to the technology of using generative AI models to create digital content such as text, images, audio, and video [47]. AIGC animation (AI-generated animation) refers to the specific application of AIGC technology in the field of animation production, involving the use of generative AI tools to complete some or all stages of the animation production process, including AI-assisted screenwriting, text-to-image generation, image-to-video generation, AI voice-over, and intelligent editing, etc. [48]. Compared to traditional animation production, AIGC animation enables the rapid generation of character designs, scene concept art, and dynamic shots through prompt control and reference image constraints, significantly reducing art production costs and shortening production cycles. In recent years, it has become an emerging technological approach for the digital dissemination and creative development of cultural heritage.

This chapter explores AIGC animation production based on the gold and silver artifacts dataset from the Tomb of the Prince of Liangzhuang, with the primary aim of validating the dataset’s application value beyond LoRA model fine-tuning: cultural relic images, collected according to standardized procedures and annotated using a four-dimensional system, can serve as visual reference material for AIGC creation, while the four-dimensional annotation system can also be directly converted into AI-generated prompts to support the digital dissemination of cultural heritage. It should be noted that the animation production in this case study primarily utilized general-purpose AI image and video generation tools; the LoRA model trained in Section 4 has not yet been directly involved in animation generation, and the relevant integration work is left for future research.

### 6.1. AIGC Animation Design Workflow

**Figure 15:**
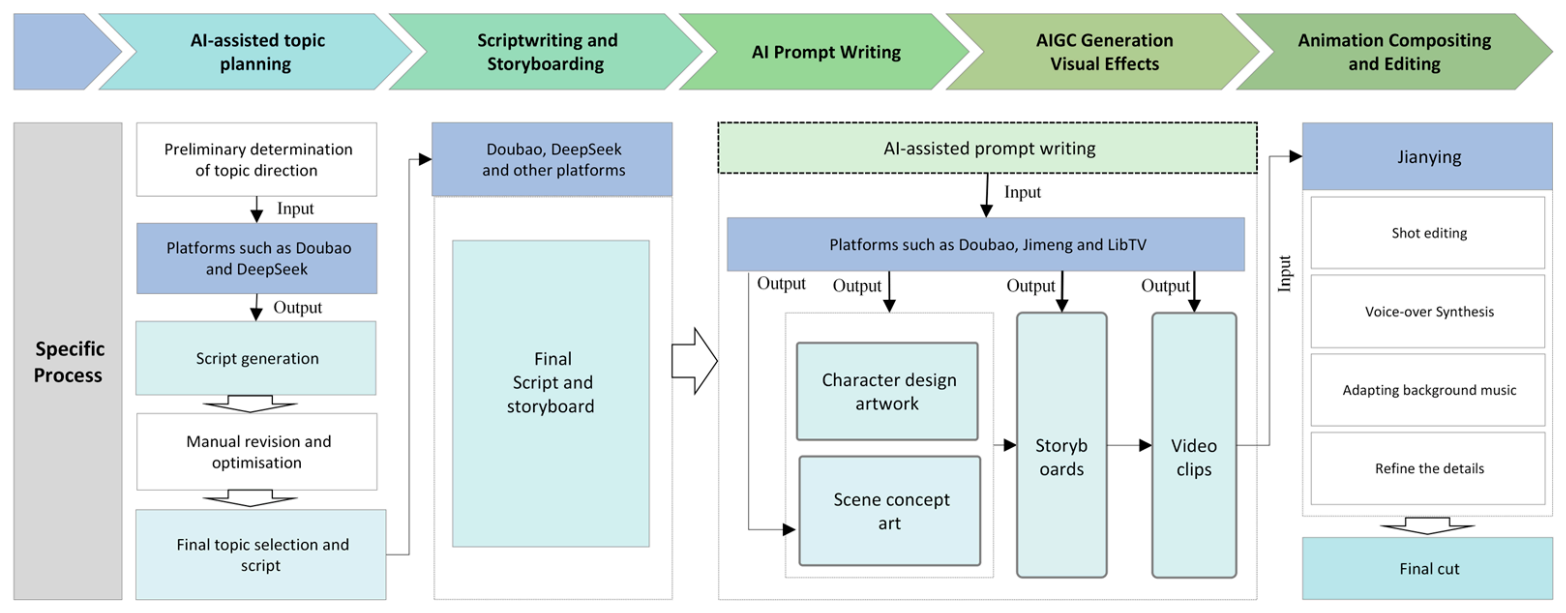
AIGC animation design process.

The AIGC animation design workflow established in this study comprises five stages, namely topic selection and planning, script and storyboard creation, AI prompt writing, visual concept art generation, and animation compositing and editing, as shown in Figure 15.

#### 6.1.1. Stage One: AI-Assisted Topic Planning

Preliminary topics were identified through brainstorming, and AI tools such as Doubao and DeepSeek were utilized to expand ideas. Drawing inspiration from the gold and silver artifacts found in the Tomb of Prince Liangzhuang, multiple creative proposals for animation scripts were rapidly generated, centering on the historical stories and cultural significance behind these cultural artifacts.

For this case study, the main storyline selected was ‘Prince Liangzhuang personally crafting a hairpin as a token of love for his consort’. A restrained classical Chinese three-dimensional animation style was adopted as the visual basis of the case study. The central prop in the story is a gold filigree hairpin adorned with jade and precious stones, featuring twin phoenixes and peony motifs. It should be noted that this plot is a work of artistic fiction and is not based on historical fact; the end credits of the finished film state: ‘The plot of this film is a work of artistic creation’.

#### 6.1.2. Phase Two: Script and Storyboard Creation

Based on the selected proposal, the plot structure was refined; AI tools such as Doubao and DeepSeek were used to write a complete written script and convert this into a professional storyboard containing shot types, duration, visual content, dialog, and sound effects.

#### 6.1.3. Phase Three: Drafting AI Prompts

Structured prompts are central to AI-generated content, serving to constrain the visual style and feature accuracy of the generated content’s features. This study has developed a prompt framework for five categories of generation tasks: prop design, character design, scene design, storyboard keyframes, and dynamic video generation. Notably, the descriptions of cultural artifact props strictly adhere to the four-dimensional annotation standards proposed in Section 3, and image generation is based on reference images of cultural artifacts from the dataset, thereby ensuring the accuracy and consistency of the generated results across shots. The specific design schemes for prompts at various levels, along with typical shot application examples, will be detailed in Section 6.2.

#### 6.1.4. Stage Four: AIGC Generation of Visual Concept Art

The optimized prompts are input into AI painting tools such as Jimeng to batch-generate character design art, scene concept art, and keyframe storyboard visuals.

#### 6.1.5. Stage Five: Animation Compositing and Editing

AI-generated materials are manually refined; AI video tools such as LibTV are used to convert keyframes into animated clips; professional editing software such as CapCut is then utilized to complete shot splicing, voice-over synthesis, and background music adaptation, ultimately outputting the complete animated film.

#### 6.1.6. Experimental Platform and Tool Description

Model training, image generation, video production, and text-assisted tasks in this study were all carried out using existing commercial platforms and professional software. The functional roles of each tool and their application scenarios in this study are as follows:

Doubao Large Language Model (Doubao): Doubao is a general-purpose large language model developed by ByteDance, capable of Chinese text generation, creative ideation, and information integration. In this study, the model was utilized during the topic planning and scriptwriting stages to assist in generating creative concepts for the script, written scripts, and storyboard copy.

LibTV: LibTV is an AI video creation platform launched by LiblibAI. It utilizes an infinite canvas and a node-based workflow, supporting the generation of dynamic video clips using reference images as input. In this study, the platform was used to convert storyboard keyframes into animated shots, while controlling camera movements and motion intensity via parameters.

Jimeng AI: Jimeng is an AI creative platform under ByteDance, supporting functions such as text-to-image, image-to-image, and image editing. In this study, the platform was used to batch-generate static visual assets such as character designs, scene concept art, and storyboard keyframes.

CapCut Pro: CapCut is a professional-grade non-linear video editing software developed by ByteDance, offering post-production functions such as clip splicing, transition design, audio mixing, subtitle creation, and color grading. In this study, the software was used to complete shot sequencing, voice-over and soundtrack editing, and final video output.

### 6.2. Examples of Prompts and Generation Workflows

**Figure 16:**
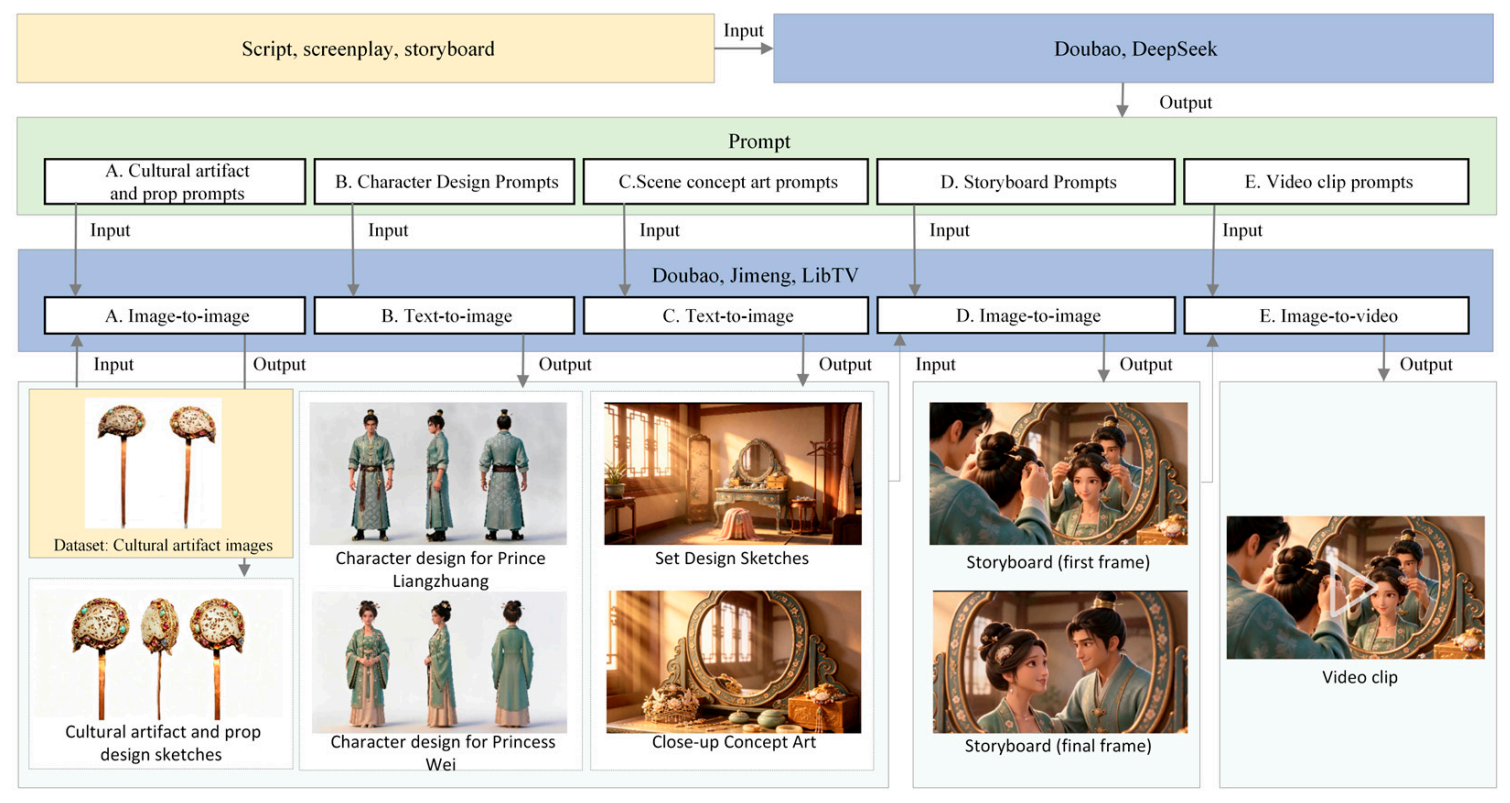
Schematic diagram of prompt and generation workflow.

The following example illustrates the prompt writing and generation process using the scene description of ‘Before Princess Wei’s dressing table, Prince Liangzhuang adorns her with a golden filigree hairpin inlaid with jade and precious stones, featuring twin phoenixes and peony motifs to frame her temples’, as shown in Figure 16.

#### 6.2.1. Generation of Cultural Artifact and Prop Setting Diagrams

Generate standardized multiview reference images of cultural artifacts to serve as visual reference diagrams for props throughout the film, thereby eliminating prop inconsistencies between different shots.

Input reference image: Photographs of the corresponding cultural artifact from the dataset.

Prompt template: Name of cultural artifact + View specifications + Form and structure + Ornamental patterns + Material representation + Color scheme + Output specifications.

Prompt example: Gold filigree hairpin with jade and gemstone inlays featuring twin phoenixes and peony motifs, arranged side-by-side in three isometric views (front, side, back); The overall shape follows a cloud-like outline, featuring a central jade element surrounded by gemstones; the surface of the jade element is carved with symmetrical twin phoenix motifs and intertwined peony patterns, while the gold border features a filigree woven mesh texture. The metal base is made of gold; the gemstones set include rubies, sapphires, turquoise, and pink gemstones, while the white jade has a warm, lustrous matte finish; the overall color scheme is dominated by warm gold tones, complemented by the white of the jade and the colorful accents of the gemstones, with a pure white, seamless background with uniform, shadow-free lighting, as well as museum-quality texture rendering; the details are clear and sharp; the three views are highly consistent in both proportion and style.

#### 6.2.2. Generation of Character Concept Art

Generate standardized multiview character design assets, fixing the character’s facial features, attire, and physical characteristics to provide character specifications for subsequent storyboard generation.

Prompt template: Identity and age + facial features and demeanor + view specifications + hairstyle and headwear + clothing style + posture and stance + output specifications.

Prompt example:

Character Design Prompt for Prince Liangzhuang: Zhu Zhanji, a feudal prince of the Ming Dynasty; a 29-year-old male; with a gentle and dignified countenance, refined features, and a calm, reserved demeanor; three orthographic views arranged side by side (front, side, back), standing naturally with an upright posture; hair tied back and adorned with a golden headpiece; wearing a bean-green cross-collared robe with a subtle intertwined floral pattern, the lapels and front placket trimmed with gold edging, featuring three circular gold buttons arranged vertically; a dark brown leather belt is fastened at the waist, featuring a circular golden pendant and brown tassels; the cuffs are rolled up to the forearms, with plain gold bracelets adorning the wrists; he wears trousers in a matching color scheme and black cloth shoes with cloud-shaped toes, the toes adorned with golden patterns; a pure white seamless background, soft overhead lighting without shadows, rendered in a classical three-dimensional style; the fabric texture is delicate, the character’s proportions are well-balanced, and the appearance is highly consistent across the three-view diagrams.

Character Profile for Princess Wei: Princess Wei, consort to a feudal prince of the Ming Dynasty, a 21-year-old woman; she possesses a gentle and delicate countenance, with soft eyes and brows, and a dignified, serene demeanor; three-view diagrams (front, side, and back) arranged side by side to scale; she stands naturally with her hands clasped in front of her abdomen, her posture serene; her hair is styled into twin spiral buns, adorned with a golden plum-blossom hairpin and a pearl buyao hair ornament; she wears teardrop-shaped pearl earrings and a delicate gold chain with a pearl choker around her neck; she is clad in a bean-green wide-sleeved jacket, the body of which is embroidered with pale beige scrollwork floral patterns, while the collar and cuffs are trimmed with gold meander-patterned edging; underneath, she wears an apricot-colored waist-length tunic and skirt, with a court sash of the same color tied at the waist, from which hangs a white pearl pendant; on her feet are off-white plain cloth shoes; against a pure white seamless background, with soft, shadowless top lighting, rendered in a classical three-dimensional style, the silk fabric has a realistic texture, the figure’s proportions are harmonious, and the appearance is highly consistent across the three-view diagrams.

#### 6.2.3. Generation of Scene Environment Reference Images

Generate a clean, character-free reference image of the scene, with a fixed spatial layout, furnishings, and lighting atmosphere, to provide a visual reference for subsequent storyboard integration and ensure consistency in the scene’s style across shots.

Prompt Template: Space Type + Key Furnishings + Decorative Details + Lighting Atmosphere + Output Specifications.

Prompt example: A dressing room in the inner courtyard of a Ming Dynasty princely mansion, a private interior space; at the center of the frame stands a blue-lacquered dressing table with gold inlay, upon which rests a copper mirror in the shape of a begonia, a multi-tiered gold-lacquered cosmetic chest, porcelain tea sets, and scattered jewelry; to the left are four lattice-paneled windows, beside which stands a wooden-framed screen painted with detailed depictions of orchids and other flowers; on the right is a wooden clothes rack draped with a pale pink embroidered silk scarf, while the floor is covered with a carpet featuring a subtle pattern; warm golden morning light streams diagonally through the lattice windows, creating a distinct Tyndall effect; the overall color scheme is warm, soft, and elegant, with fine wood grain and a clearly defined spatial hierarchy; the rendering adopts a classical three-dimensional style with cinematic, soft lighting and shadows, creating a tranquil and cozy atmosphere; the image contains no figures, subtitles, or watermarks.

#### 6.2.4. Generation of Keyframes for Narrative Storyboards

Integrate three categories of reference material—characters, scenes, and props—to generate narrative-driven single frames, which serve as direct input for dynamic video generation. Multiple reference constraints ensure that all elements remain consistent with the pre-production specifications.

Input reference images: Character design art, scene design art, cultural artifact and prop design artPrompt template: shot specifications + character actions + prop presentation + scene matching + lighting and mood + stylistic requirements

Prompt example: Medium-close-up shot, mirror-symmetrical composition; Prince Liangzhuang of the Ming Dynasty stands behind Princess Wei, gently fastening a gold filigree hairpin inlaid with jade and precious stones, featuring twin phoenixes and peony motifs, to her hair; Princess Wei sits upright before her dressing table, facing a bronze mirror, which reflects the tender expressions and smiles of both figures; the design, ornamentation, and materials of the hairpin are entirely consistent with cultural artifact specifications, and it is positioned accurately; The characters’ appearances and attire are entirely consistent with the character designs, while the scene layout and lighting perfectly match the concept art; warm, golden morning light streams in through the floral window on the left, casting soft highlights on the characters’ hair and the surfaces of the objects; the overall atmosphere is gentle and serene, with the emotional expression being subtle and restrained; the rendering style is classical 3D, with the characters and scene blending naturally and the lighting and shadows harmonized.

#### 6.2.5. Generation of Dynamic Video Clips

Using storyboard keyframes as a foundation, generate continuous dynamic video clips to transform a single shot from a static image into moving footage.

Input reference image: Keyframe images corresponding to the storyboard.

Prompt template: Camera movement + moving objects + stability constraints + consistency requirements.

Prompt example: The camera moves slowly forward in small increments, with the focus remaining on the character and the hair ornament; Prince Liangzhuang’s hand movements are slow and gentle as he performs the subtle action of fastening the hair ornament; Princess Wei’s head remains still, a smile gradually appearing on her face; the curtains and strands of hair flutter gently in the breeze, while dust particles in the beam of light streaming through the window slowly settle; throughout the sequence, the forms of the characters’ faces, the hair ornament, and the set décor must remain stable, with no distortion or drift in distinctive features; the cloud-shaped outline of the hair ornament and the structure of the twin phoenix motifs must remain clear and intact throughout; transitions between frames must be smooth and natural, with the overall color tone, lighting, shadows, and style closely matching the reference image, and the footage must be fluid with no stuttering.

### 6.3. Presentation of Results

This chapter utilizes the image dataset of gold and silver artifacts from the Tomb of the Prince of Liangzhuang, constructed as part of this research, as source material to explore applications in the field of AIGC animation. The study outlined the animation production workflow comprising five stages: topic planning, script and storyboarding, prompt design, visual generation, and post-production compositing. It summarized standardized prompt templates covering five task categories—prop design, character design, scene design, storyboard keyframes, scene design, and dynamic video—and used these as the basis for completing the animation production. This animation case study will be uploaded to a public platform alongside the dataset, serving as an accompanying application example to provide a reference for subsequent similar research and creative work.

This project has preliminarily validated the practical value of specialized cultural heritage image datasets in digital creative contexts: datasets that have undergone standardized processing and structured annotation can serve not only as a data foundation for model training but also as a reference repository for AIGC creation. Furthermore, this initiative has explored a pathway for animation creation within the field of digital heritage conservation, enabling the dissemination of cultural heritage craftsmanship and cultural significance to the general public through audiovisual means.

It should be noted, however, that the animation generation in this study still relies on the capabilities of the base models provided by commercial AIGC platforms; the dataset serves only as a constraint at the single-frame reference level, and the LoRA fine-tuned model trained in this study has not yet been integrated into the generation process. Future work will seek to further integrate the SDXL_LoRA fine-tuned model into animation production, thereby forming a more comprehensive application solution for the digital conservation of cultural artifacts.The animated short film produced in this chapter has been uploaded to IEEE DataPort alongside the image dataset, serving as a supplementary demonstration of the dataset’s application potential in AIGC content creation.

## 7. Conclusions and Outlook

### 7.1. Research Conclusions

This study focused on the digital preservation and intelligent generation of gold and silver artifacts from the Tomb of Prince Liangzhuang. A specialized fine-grained image dataset was constructed, a four-dimensional visual feature annotation system was developed, and LoRA fine-tuning was conducted on three diffusion models, followed by preliminary generation experiments. The main contributions and findings of this study can be summarized in four aspects.

First, a fine-grained image dataset tailored to the gold and silver artifacts from the Tomb of Prince Liangzhuang was constructed. The data were sourced from publicly available resources on the official website of the Hubei Provincial Museum and photographs acquired on-site in the exhibition halls. The dataset covers 82 gold and silver artifacts and, after systematic data cleaning, image correction, and standardization, contains 4127 panoramic images and 322 detail images. The artifacts are organized into five major categories: Headwear, Headgear and Sashes, Accessories, Daily Necessities, and Ritual Objects. A four-dimensional visual feature annotation system was applied to provide structured annotations for form and structure, decorative patterns, material appearance, and color composition. The resulting dataset provides a standardized data resource for the digital preservation and intelligent generation of gold and silver artifacts.

Secondly, a four-dimensional annotation system for the visual features of gold and silver artifacts was established. This system was developed based on a systematic review of archeology, design studies, and computer vision, synthesizing the disparate descriptions of gold and silver artifacts from various disciplines into a unified annotation framework suitable for computer vision processing. Through comparative experiments involving general annotations (Group A), detailed annotations (Group B), and the core four-dimensional annotations (Group C), the four-dimensional annotation system was validated as achieving the optimal balance between feature comprehensiveness and model generalization: general annotations lack sufficient information, while detailed annotations introduce variables from the shooting environment, leading to overfitting; the four-dimensional annotations, however, precisely cover the primary visual features of gold and silver artifacts. This annotation framework is applicable to different types of cultural artifacts and can serve as a methodological reference for the digital annotation of other three-dimensional crafts.

Third, the suitability of three diffusion models for gold and silver artifact generation was systematically compared. LoRA fine-tuning was conducted on SD1.5, SDXL, and FLUX, and 1968 images were generated across the five artifact categories using three fine-tuned models and eight fixed random seeds. The generation results were evaluated through visual comparison, quantitative metrics, and subjective evaluation. The results showed that SD1.5_LoRA achieved the highest mean SSIM (0.627) and the lowest mean seed-to-seed standard deviation (0.050), indicating relatively high structural similarity and generation stability under the evaluated conditions. SDXL_LoRA received the highest equally weighted vote percentage for Overall Effect in the subjective evaluation, while FLUX_LoRA received higher vote percentages for Material Appearance and Color Composition. The CLIP Score showed category-dependent differences rather than a consistent overall ordering of the three models. Significant within-category pairwise differences were observed in Headwear, Daily Necessities, Headgear and Sashes, and Ritual Objects, whereas no significant pairwise differences were observed among the three models in Accessories. The equally weighted mean CLIP Scores of the three models were relatively close (31.21, 29.97, and 30.24), suggesting broadly comparable text–image semantic alignment under the evaluated conditions, with some category-dependent variation. The differences between the objective and subjective evaluations indicate that image-based similarity metrics and human evaluation capture partially different aspects of generation quality. The resolution comparison experiment further showed no statistically significant differences between 768 × 768 and 1024 × 1024 under the evaluated conditions.

Fourthly, we have preliminarily explored feasible pathways for the application of the dataset in AIGC animation. The study established an animation design workflow and produced a short animated film, providing a reference case for the creative transformation of cultural heritage datasets.

### 7.2. Limitations of the Study

This study has the following limitations.

First, limitations related to the size and composition of the reference set and the use of distribution-level metrics. The reference set contains 1004 reference images representing 82 cultural artifacts, while 1968 images were generated across the three fine-tuned models and eight fixed random seeds. Although the reference set covers all five artifact categories, the number and composition of reference images within individual categories remain limited for reliable estimation of distribution-level distances such as FID and KID. Therefore, this study primarily relies on image-level paired metrics (SSIM and LPIPS) and subjective evaluation, while FID and KID are treated as exploratory evidence rather than as the primary basis for model comparison or statistical inference.

Second, imbalance in sample size across artifact categories. The numbers of artifacts vary considerably among the five categories, ranging from 28 Daily Necessities artifacts to 11 Ritual Objects artifacts. This imbalance may affect the representativeness of category-level results and the interpretation of equally weighted aggregate results. A larger and more balanced dataset would provide a stronger basis for evaluating model performance and generalization across different artifact categories.

Thirdly, the potential contribution of detailed images has not yet been fully explored. Preliminary experiments indicated that including detailed images in the training set led to misalignments between patterns and artifact forms; consequently, this study excluded them from training. However, as detailed images have been proven effective as visual reference inputs during the design phase, their potential value in model training warrants further investigation.

Fourthly, image sources are constrained by exhibition conditions. The image quality of some cultural artifacts varies due to factors such as reflections from museum display case glass, uneven lighting, and restricted shooting angles, which may affect the model’s ability to learn the features of certain objects.

### 7.3. Future Work

Regarding dataset expansion, given the distinct characteristics of the three models, future work will explore image generation methods based on model fusion and extend the four-dimensional annotation system to gold and silver artifacts from other historical periods and regions, thereby establishing a more universal digital annotation framework for gold and silver artifacts.

In terms of model optimization, given FLUX_LoRA’s shortcomings in structural stability and its strengths in material representation, future work could explore image generation via model fusion. This could involve using FLUX to generate material details and then fusing these with the structural outputs of SDXL, or adopting multi-model ensemble methods to enhance overall generation quality.

With regard to the application of detail images, to address the issue of unstable training results when mixing detail images, future work could involve attempting a phased training strategy—first using panoramic images followed by detail images—on platforms or in local environments that support two-stage fine-tuning. The first stage would use panoramic images to establish an overall structural understanding, while the second stage would utilize detail images to fine-tune the precision of decorative patterns and textures, thereby verifying the potential of detail images in refining decorative features.

In terms of technological expansion, future work could explore extending this method from 2D image generation to 3D model generation. By combining this with 3D scanning technology, a complete digital reproduction of gold and silver artifacts could be achieved, providing more comprehensive data support for virtual exhibitions and digital archeology.

In terms of design applications, future work could involve integrating dataset creation with AIGC animation, while refining annotation and prompt standards to further lower the barrier to entry for AI-assisted design.

The complete dataset, LoRA weights, image generation prompts, and AIGC animations from this study have been published at https://ieee-dataport.org/documents/dataset-gold-and-silver-artifacts-tomb-prince-liangzhuang (accessed on 3 October 2026).

## Figures and Tables

**Table 1:** Development of gold and silver artifacts worldwide.

Civilisation /Region	Period	Key Characteristics	Representative Artifacts
Mesopotamia	c. 4000 BC–539 BC	Origins of the gold granulation and filigree techniques	Golden bull-headed lyre from the Royal Tombs of Ur
Ancient Egypt	3100 BC–30 BC	Funeral gold artifact, hammered and inlaid	The golden mask of Tutankhamun
Ancient Greece	1600 BC–146 BC	Gold mask and crown, realistic style	Mask of Agamemnon
Etruria	700 BC–100 BC	The pinnacle of granulation technique	Gold brooch from the Regolini–Galassi tomb
Ancient Rome	753 BC–476 AD	Silverware became part of everyday life, evolving into tableware sets	Boscoreale silverware
Sassanid Persia	224–651	Interlaced bead and winged beast motifs, influencing East Asia	Gilded silver jug from the tomb of Li Xian at Guyuan
Byzantine	330–1453	Cloisonné enamel, religious goldware	Double-sided pendant icon
Islamic World	7th–17th centuries	Geometric patterns with inscriptions, inlaid with gold and silver	Fatima gold earrings
Renaissance Europe	14th–17th centuries	A fusion of goldsmithing and sculpture	Cellini gold salt cellar

**Table 2:** The development of Chinese gold and silverware.

Dynasty	Period	Key Characteristics	Representative Artifacts
Shang and Zhou Dynasties	c. 1600 BC–256 BC	Gold foil hammered ornaments; the ancient Shu civilization was renowned for its goldwork	Sanxingdui gold staff
Spring and Autumn and Warring States Periods	770 BC–221 BC	The flourishing of gold and silver inlay, with exquisite craftsmanship	Gold cup from the tomb of Zeng Hou Yi
Qin and Han Dynasties	221–220 BC	Fine craftsmanship reached maturity, establishing itself as an independent art form	Golden seal of the King of Dian
Wei, Jin, and the Northern and Southern Dynasties	220–589	Eastern spread of foreign influences; fusion of exotic styles	Gold hair ornament in the shape of a flowering tree
Tang Dynasty	618–907	The heyday, marked by a deep fusion of Chinese and Western cultures	Dancing horse holding a cup vase
Song Dynasty	960–1279	Secular and minimalist, simple yet elegant	Southern Song Dynasty gilded silver box with double-phoenix motif in sunflower-petal shape
Yuan Dynasty	1271–1368	Scholarly refinement, a succession of master craftsmen	Zhu Bishan’s silver ‘Driftwood’ cup
Ming Dynasty	1368–1644	The pinnacle of craftsmanship, used by the imperial court	The golden-threaded Yishan crown from Dingling
Qing Dynasty	1636–1912	The pinnacle of filigree and enamel inlay, exquisitely ornate and magnificent	‘Eternal Stability’ gold cup

**Table 4:** Research on the application of generative AI in cultural heritage design.

Research Subject	Research Team	Core Method	Sample Size	Generation Objectives
Miao batik	Hu, Q. et al., 2025 [6]	Diffusion Model + LoRA + LCM + SCLF	649 images	Style transfer of batik patterns
Bamboo weaving patterns	Kang, X. et al., 2026 [16]	StyleGAN + Grasshopper	2472 images	Generation of bamboo weaving patterns and product design
Lantern Art	Zhou, C.et al., 2025 [18]	Diffusion Model + AIlantern Tool	18 patterns	Rendering and development diagram generation for lanterns
Chinese esthetic jewelry	Cheng, Z. et al., 2025 [20]	LoRA + ComfyUI	423 images	Jewelry design style generation
Buddha statues	Zhang, S. et al., 2025 [21]	Domain-Adaptive Diffusion Model + Multiview Generation + ControlNet Refinement + ISOMER Grid Reconstruction	672 statues	High-fidelity 3D reconstruction from a single image

**Table 5:** Global research on gold and silver artifacts.

Research Areas	Representative Scholars/Teams	Core Methods	Research Content	Data Basis
Production techniques	Natuniewicz–Sekula et al. [22]	Experimental archeology	Reconstruction of ancient forging techniques	Physical Observation
	Scrivano et al. [27]	SEM-EDS, micro-XRF	Ancient Welding and Brazing Techniques	Physical Examination
	Darque–Ceretti et al. [28]	Comparative study	Evolution of gilding techniques	Literature + Physical Specimens
Source of materials	Guerra [23]	Chemical analysis	Recovery and reuse of gold	Physical Examination
	Eshel et al. [29]	Lead isotope analysis	Phoenician metal trade network	Physical Examination
	Numrich et al. [30]	Portable laser ablation	The origin of bronze age gold artifacts	Physical Examination
Case studies	Buccolieri et al. [24]	EDXRF	Gold jewelry from the ancient Greek colony of Taranto	Physical Examination
	Guerra et al. [25]	Compositional analysis	Gold jewelry from Akhotep, Egypt	Physical Examination
	Papadimitriou et al. [26]	Multidisciplinary approach	Mycenaean ‘gold embroidery’ technique	Physical Examination + Experimentation

**Table 6:** Research on Chinese gold and silverware.

Research Areas	Leading Scholars/Research Teams	Core Methods	Research Content	Data Basis
Archeological Typology	Zhang, J., Qi, D. [33]	Typological analysis	A General History of Gold and Silver Artifacts and the Evolution of Form	Archeological Reports
History of Arts and Crafts	Yang, Z. [31]	Cross-verification of texts, images and artifacts	Gold and Silver Jewelry of the Song, Yuan and Ming Dynasties	Literary Sources + Artifacts
Material Analysis	Yang, M. [32]	XRF, SEM-EDS	Analysis of Jewelry and Jade Artifacts from the Tomb of the Prince of Liangzhuang	Physical Examination
Innovative Pattern Design	Zhang, H. et al. [34]	Semantics and morphological grammar of Miao fish-motif rubbing	Design Methodology	Miao Patterns

**Table 7:** Current state of research on gold and silver artifacts from the Tomb of the Prince of Liangzhuang.

Research Type	Representative Achievements	Core Content	Methods and Approaches	Data Basis
Archeological Excavations	Hubei Provincial Institute of Cultural Relics and Archeology, The Tomb of the Prince of Liangzhuang [35]	Tomb Layout, Funerary Goods Assortment and Excavation Findings	Field Archeology	Physical artifacts and written records
History of Arts and Crafts	Yang Zhao, ‘A Study of the Gold and Silver Artifacts Unearthed from the Tomb of the Prince of Liangzhuang of the Ming Dynasty’ [36]	Functional classification, Inscriptions, Materials, Forms, and craftsmanship	Analysis from the Perspective of the History of Decorative Arts	Observation of physical artifacts + secondary sources
Materials Science	Yang Mingxing’s team [32]	Scientific Testing and Spectroscopic Analysis of Jewelry and Jade Artifacts	XRF, SEM-EDS	Physical testing data
Material Cultural Exchange	Hubei Provincial Museum: ‘The Tomb of the Prince of Liangzhuang: Treasures from the Age of Zheng He’ [37]	A Study of Gold Ingots Associated with Zheng He’s Voyages to the Western Seas	Archeology + History	Inscriptions + historical texts
Digital Preservation	Hubei Provincial Museum Digital Collection, Online Thematic Exhibitions	Cultural Heritage Documentation, Digital Exhibition Design	Digital Archiving	Collection images

**Table 8:** Four-dimensional annotation system for gold and silver artifacts from the Tomb of the Prince of Liangzhuang.

Feature Annotation Dimension	First-Level Categories	Second-Level Categories	Tertiary Categories	Examples of Representative Artifacts
Form and Structure	Object Type	Headwear	Hairpins, hairpins with clasps, hairpins for covering the temples, temple hairpins, floral hairpins, phoenix hairpins, gold-inlaid floral ornaments, hairpins with a central divider, gold phoenix hairpins	Gold phoenix hairpins, peony flower hairpins
		Headwear and Sashes	Cap finials, sash rings, gold sashes, gold buckles, gold hooks, leather ceremonial caps, ceremonial headwear	Gold-inlaid blue-and-white jade ribbon rings with dragon motifs
		Accessories	Earrings, rings, bracelets, armbands, pendant beads, gold pendants, gold ritual rings, jade pendants, gold ear spoons, gold tweezers, gold toothpicks, gold spoons	Strung Jewelry Gold earrings, gold rings set with rubies
		Daily necessities	Jugs, basins, bowls, ritual vessels, ladles, chopsticks, spoons, boxes, lids	Gold teapots, gold basins
		Ritual objects	Buddha statues, vajra, mantra plaques, prayer beads, deities’ statues	Gold Garuda statue, gold dancing Shakra statue
	Morphological characteristics	Overall Silhouette	Phoenix-shaped, melon-shaped, cloud-head-shaped, peach-shaped, plum-blossom-shaped, triangular, gourd-shaped	Golden phoenix hairpin (phoenix-shaped), muskmelon hairpin (melon-shaped)
	Structural Characteristics	Component Composition	Segmented (hairpin head + shaft), encircling (central body + surrounding elements)	Golden Phoenix Hairpin (three sections: head, body, and tail), Inlay (central jade piece + surrounding gemstones)
		Hierarchical Relationships	Superimposition, nesting, openwork	Gold filigree with jade and gemstone inlays, depicting twin phoenixes and peonies adorning the temples
	Artistic Expression	Openwork Effect	Openwork carving, openwork	Gold Openwork Phoenix-Patterned Pendant
		Weaving Effect	Gold-thread weaving, silver-thread weaving	Gold-Inlaid Floral Motifs
		Mesh effect	Wire-woven mesh	Gold filigree gem-set crown
		Relief effect	Embossed floral patterns, high-relief floral patterns	Gold Bell Lid
		Hammered effect	Hammered and stretched into sheet form, then shaped into three-dimensional forms	Gold teapots, gold basins
Decorative patterns	Traditional motifs	Natural motifs	Animal motifs (dragon and phoenix motifs, phoenix motifs, twin phoenix motifs, fish motifs, butterfly motifs); plant motifs (floral motifs, fruit motifs, interlaced floral motifs, scrollwork); geometric motifs (cloud motifs)	White jade cloud-and-dragon cap finial set with gold and gemstones; gold filigree centerpiece with jade-inlaid twin phoenixes and peony motif; base of a gold phoenix hairpin
		Religious Motifs	Buddhism (Sanskrit seed syllables, vajra motifs, Garuda, golden mandalas); Tibetan Esoteric Buddhism (Mahakala)	Gold Sanskrit seed syllables, gold Garuda figure, gold Kalachakra Mandala mantra plaque
decorative patterns	Metal	Precious metals	Gold, silver	Gold Dharma Vows Ring
	Gemstone Materials	Colored gemstones	Rubies, sapphires, emeralds, crystal, turquoise	Gold-set sapphire cap
		Organic gemstones	Pearls	Strung jewelry Gold earrings
	Other materials	Jade varieties	Jade, white jade, blue-white jade, green jade	Gold-set glass relief earring featuring two lions playing with a ball
Color schemes	Warm color scheme	Warm colors predominate, with cool colors as accents	Predominantly golden yellow, accented with red, pink, and purple gemstones	Gold filigree gemstone-set band
		Warm colors predominate, with combinations within the same color family	Golden yellow as the main color, with warm gold as an accent	Gold jug
	Cool color scheme	Predominantly cool colors, with warm colors as an accent	Predominantly silvery-white, accented with blue and green gemstones	No such gold and silver objects have been found among the gold and silver artifacts from the Tomb of Prince Liangzhuang
		Predominantly cool colors, with combinations within the same color family	Silver-white as the main color, with a touch of bluish-white	Silver-plated apricot-leaf-shaped vessel
	Medium tones	Neutral colors as the main theme, with warm colors as accents	White (white jade) as the main color, with gold as an accent	Gilded White Jade Gourd Ornament
		Neutral colors predominate, accented with cool tones	White (white jade) as the main color, with blue–green (turquoise) as an accent	Gold Filigree Hairpin with Jade and Precious Stones, Featuring Twin Phoenixes and Peonies Adorning the Temples

**Table 9:** Example of image annotation.

Feature Dimension	Primary Category	Second-Level Classification	Annotation Term
Form and Structure	Object type	Hairpin	Gold temple hairpin
	Morphological characteristics	Overall contour	Cloud-shaped overall form
	Structural characteristics	Component composition	Hollow, carved white jade center surrounded by gemstones
	Stylistic expression	Openwork effect	Filigree wire mesh texture, hollow openwork texture, gem and jade bezel inlay texture
Decorative patterns	Animal motifs	Pair of phoenixes	Paired phoenix pattern
	Floral pattern	Peony pattern	Peony pattern
Material representation	Metal texture	Gold	Gold
	Gemstone materials	Turquoise, pink gemstone, lavender-blue gemstone	Turquoise, pink gemstone, lavender-blue gemstone
	Other materials	White jade	White jade
Color schemes	Warm tones	Warm colors as the main theme, with cool colors as an accent	Warm golden tones with accents of white, turquoise, blue, green, pink, and lavender blue

**Table 10:** Experimental setup for the comparison of annotation and training strategies across the three groups.

	Group A (Generalized Annotation)	Group B (Detailed Annotation)	Group C (Core Four-Dimensional Annotation)
Annotation Dimensions	Brief four-dimensional annotation	Four-dimensional annotation + all describable information such as symmetry, light direction, shooting angle, and color temperature	Annotation of only the most prominent features of each of the four dimensions
A1/B1/C	Panoramic images only (162 images)	Panoramic images only (162 images)	Panoramic images only (162 images)
A2/B2	Panoramic images (162) + detailed images (22)	Panoramic images (162) + detailed images (22)	—
Purpose	To test whether generalized annotations are sufficient for the model to learn the characteristics of gold and silver artifacts	To investigate whether annotating in as much detail as possible improves generation quality	To validate the effectiveness of a moderately focused four-dimensional annotation strategy

**Table 11:** Dataset size by artifact category.

Artifact Category	Number of Cultural Artifacts	Dataset Size	Panoramic Images	Detail Images
Headwear	11	822	800	22
Headgear and Sashes	19	1070	927	143
Accessories	13	845	800	45
Daily Necessities	28	864	800	64
Ritual Objects	11	848	800	48
Total	82	4449	4127	322

**Table 12:** LoRA fine-tuning parameter configuration.

Parameter Category	Parameter Value
UNet backbone learning rate *	1 × 10^−4^
Text encoder learning rate *	5 × 10^−5^
LoRA rank	64
LoRA Alpha	32
Scaling factor (Alpha/Rank)	0.5
Optimizer	AdamW8bit
Learning Rate Scheduling	Cosine
Batch size	2
Number of training epochs	8
Image resolution	768 × 768

Note: * The U-Net backbone and text encoder learning rates apply to SD1.5 and SDXL. FLUX uses a global learning rate of 5 × 10^−5^ because its architecture does not employ separate U-Net and text encoder branches with independently assigned learning rates.

**Table 13:** Experimental dataset and generation allocation.

Artifact Category	Number of Artifacts	Training Images	Reference Images	Generated Images per Artifact	Generated Images per Model	Total Generated Images
Headwear	11	600	200	8	88	264
Headgear and Sashes	19	600	200	8	152	456
Accessories	13	589	200	8	104	312
Daily Necessities	28	600	204	8	224	672
Ritual Objects	11	600	200	8	88	264
Total	82	2989	1004	8	656	1968

Note: Of the 4127 panoramic images, 2989 were used for LoRA fine-tuning, 1004 were used as the reference set for generation evaluation, and the remaining 134 were not used in either training or evaluation. The five artifact categories were trained independently, with a maximum of 600 images uploaded for each category under the platform constraints. The Accessories category contained 589 training images because of the platform upload limit. The reference images did not overlap with the training images. The 134 unused images were excluded because the number of images exceeded the combined requirements of the category-specific training limit and the approximately 200 reference images selected for each category.

**Table 14:** Core metrics for the daily necessities category.

Model	SSIM ↑	LPIPS ↓	CLIP Score ↑	Seed-to-Seed SD ↓
FLUX	0.522 ± 0.200 [0.450, 0.593]	0.623 ± 0.167 [0.565, 0.685]	**31.338 ± 2.610** [30.405, 32.219]	0.114
SD1.5	**0.656 ± 0.161** [0.598, 0.719]	**0.516 ± 0.099** [0.476, 0.551]	30.191 ± 3.178 [28.997, 31.279]	**0.053**
SDXL	0.515 ± 0.194 [0.446, 0.588]	0.655 ± 0.144 [0.600, 0.706]	31.140 ± 2.537 [30.195, 32.024]	0.123

Note: ↑ indicates that higher values indicate better performance; ↓ indicates that lower values indicate better performance. Bold values indicate the best value among the three models.

**Table 15:** Key metrics for Accessories.

Model	SSIM ↑	LPIPS ↓	CLIP Score ↑	Seed-to-Seed SD ↓
FLUX	0.574 ± 0.176 [0.469, 0.663]	0.519 ± 0.111 [0.460, 0.584]	**32.427 ± 2.087** [31.320, 33.554]	0.119
SD1.5	**0.677 ± 0.165** [0.576, 0.754]	**0.447 ± 0.125** [0.379, 0.517]	31.307 ± 2.671 [29.875, 32.745]	**0.040**
SDXL	0.481 ± 0.129 [0.413, 0.544]	0.680 ± 0.086 [0.636, 0.725]	31.881 ± 2.567 [30.518, 33.281]	0.157

Note: ↑ indicates that higher values indicate better performance; ↓ indicates that lower values indicate better performance. Bold values indicate the best value among the three models.

**Table 16:** Key indicators for Headgear and Sashes.

Model	SSIM ↑	LPIPS ↓	CLIP Score ↑	Seed-to-Seed SD ↓
FLUX	0.470 ± 0.159 [0.402, 0.539]	0.606 ± 0.092 [0.564, 0.644]	**31.55 ± 2.22** [30.68, 32.48]	0.115
SD1.5	**0.566 ± 0.177** [0.477, 0.640]	**0.537 ± 0.106** [0.495, 0.588]	31.30 ± 2.13 [30.38, 32.27]	**0.043**
SDXL	0.430 ± 0.154 [0.362, 0.496]	0.668 ± 0.093 [0.629, 0.707]	30.19 ± 2.04 [29.32, 31.04]	0.133

Note: ↑ indicates that higher values indicate better performance; ↓ indicates that lower values indicate better performance. Bold values indicate the best value among the three models.

**Table 17:** Key metrics for Headwear.

Model	SSIM ↑	LPIPS ↓	CLIP Score ↑	Seed-to-Seed SD ↓
FLUX	0.473 ± 0.157 [0.383, 0.567]	0.601 ± 0.082 [0.557, 0.646]	**31.579 ± 2.195** [30.096, 32.536]	0.183
SD1.5	**0.679 ± 0.119** [0.609, 0.741]	**0.477 ± 0.055** [0.446, 0.509]	28.955 ± 3.117 [27.074, 30.613]	**0.050**
SDXL	0.522 ± 0.176 [0.427, 0.623]	0.614 ± 0.106 [0.552, 0.667]	30.111 ± 2.359 [28.559, 31.270]	0.152

Note: ↑ indicates that higher values indicate better performance; ↓ indicates that lower values indicate better performance. Bold values indicate the best value among the three models.

**Table 18:** Core metrics for Ritual Objects.

Model	SSIM ↑	LPIPS ↓	CLIP Score ↑	Seed-to-Seed SD ↓
FLUX	0.477 ± 0.174 [0.381, 0.570]	**0.586 ± 0.052** [0.559, 0.616]	**29.183 ± 2.770** [27.481, 30.636]	0.112
SD1.5	**0.558 ± 0.182** [0.463, 0.663]	0.552 ± 0.074 [0.507, 0.589]	28.082 ± 2.593 [26.539, 29.417]	**0.063**
SDXL	0.403 ± 0.219 [0.292, 0.522]	0.679 ± 0.109 [0.620, 0.739]	27.883 ± 2.228 [26.576, 29.136]	0.152

Note: ↑ indicates that higher values indicate better performance; ↓ indicates that lower values indicate better performance. Bold values indicate the best value among the three models.

**Table 19:** Summary of Wilcoxon signed-rank tests and effect sizes across five artifact categories (artifacts as the statistical units; Bonferroni correction, α = 0.05/3 ≈ 0.0167).

Category	Indicator	FLUX vs. SD1.5			FLUX vs. SDXL			SD1.5 vs. SDXL		
		W	*p*	r	W	*p*	r	W	*p*	r
Daily Necessities	SSIM	25	<0.001 *	−0.88	155	0.284	+0.24	0	<0.001 *	+1.00
	LPIPS	45	<0.001 *	+0.78	115	0.045	−0.43	1	<0.001 *	−1.00
	CLIP	77	0.003 *	+0.62	155	0.284	+0.24	78	0.003 *	−0.62
Accessories	SSIM	1	0.001 *	−0.98	14	0.027	+0.69	0	<0.001 *	+1.00
	LPIPS	9	0.008 *	+0.80	1	<0.001 *	−0.98	0	<0.001 *	−1.00
	CLIP	14	0.027	+0.69	21	0.094	+0.54	35	0.497	−0.23
Headgear and Sashes	SSIM	25	0.003 *	−0.74	67	0.275	+0.29	10	<0.001 *	+0.89
	LPIPS	30	0.007 *	+0.68	43	0.036	−0.55	7	<0.001 *	−0.93
	CLIP	82	0.623	+0.14	25	0.003 *	+0.74	25	0.003 *	+0.74
Headwear	SSIM	3	0.005 *	−0.91	25	0.520	−0.24	4	0.007 *	+0.88
	LPIPS	3	0.005 *	+0.91	28	0.700	−0.15	6	0.014 *	−0.82
	CLIP	6	0.014 *	+0.82	4	0.007 *	+0.88	14	0.102	−0.58
Ritual Objects	SSIM	4	0.007 *	−0.88	14	0.102	+0.58	2	0.003 *	+0.94
	LPIPS	11	0.054	+0.67	7	0.019	−0.79	2	0.003 *	−0.94
	CLIP	6	0.014 *	+0.82	6	0.014 *	+0.82	25	0.520	+0.24

Note: W denotes the Wilcoxon signed-rank test statistic; *p* denotes the unadjusted *p*-value; * indicates *p* < 0.0167 after Bonferroni correction (α = 0.05/3); r denotes the rank-biserial effect size. The sign of r indicates the direction of the comparison, whereas its absolute value indicates the magnitude of the difference.

**Table 20:** Equally weighted average results across the five artifact categories.

Model	SSIM ↑	LPIPS ↓	CLIP Score ↑	Seed-to-Seed SD ↓
FLUX	0.503	0.587	**31.21**	0.129
SD1.5	**0.627**	**0.506**	29.97	**0.050**
SDXL	0.470	0.659	30.24	0.143

Note: ↑ indicates that higher values indicate better performance; ↓ indicates that lower values indicate better performance. Bold values indicate the best value among the three models.

**Table 21:** Summary of FID and KID across the five artifact categories (exploratory reference only).

Category	Model	FID ↓	KID ± SE ↓
Daily Necessities	FLUX	139.59	0.0224 ± 0.0025
	SD1.5	**124.63**	**0.0176 ± 0.0023**
	SDXL	132.14	0.0202 ± 0.0025
Accessories	FLUX	167.60	0.0259 ± 0.0032
	SD1.5	**153.18**	**0.0234 ± 0.0031**
	SDXL	167.12	0.0276 ± 0.0030
Headgear and Sashes	FLUX	158.00	0.0320 ± 0.0032
	SD1.5	**157.85**	0.0298 ± 0.0034
	SDXL	163.08	**0.0295 ± 0.0029**
Headwear	FLUX	186.48	0.0590 ± 0.0062
	SD1.5	**141.97**	**0.0193 ± 0.0034**
	SDXL	153.58	0.0278 ± 0.0048
Ritual Objects	FLUX	179.39	0.0394 ± 0.0047
	SD1.5	**153.90**	0.0231 ± 0.0034
	SDXL	173.86	0.0265 ± 0.0037

Note: ↓ indicates that lower values indicate better performance. Bold values indicate the lowest FID and KID values within each category.

**Table 22:** Overall effect vote percentages and Friedman test results by artifact category.

Category	Subjects	Number of Artifacts	FLUX	SDXL	SD1.5	Friedman χ^2^	*p*-Value
Headwear	20	6	8.3%	**78.3%**	2.5%	28.758	<0.0001
Headgear and Sashes	20	11	**51.8%**	32.3%	2.7%	22.000	<0.0001
Accessories	20	6	**32.5%**	27.5%	1.7%	17.848	0.0001
Daily Necessities	20	13	7.3%	**72.3%**	9.2%	27.886	<0.0001
Ritual Objects	21	5	16.2%	**66.7%**	14.3%	16.137	0.0003

Note: Vote percentages represent the overall effect dimension. Bold values indicate the model with the highest vote percentage within each category.

**Table 23:** Vote percentages (%) for subjective evaluation across five dimensions and five artifact categories.

Dimension	Model	Headwear	Daily Necessities	Accessories	Headgear and Sashes	Ritual Objects	Average
Form and structure	FLUX	5.8	6.5	6.7	28.2	15.2	12.5
	SDXL	**41.7**	**55.8**	**45.8**	**40.0**	**52.4**	**47.1**
	SD1.5	33.3	18.1	7.5	18.6	29.5	21.4
Decorative Patterns	FLUX	5.8	6.9	7.5	27.7	10.5	11.7
	SDXL	**80.0**	**69.6**	**46.7**	**43.2**	**64.8**	**60.8**
	SD1.5	2.5	10.4	7.5	15.9	21.0	11.4
Material Rendering	FLUX	**55.8**	**51.9**	**51.7**	**75.9**	**64.8**	**60.0**
	SDXL	30.8	29.6	8.3	13.6	25.7	21.6
	SD1.5	1.7	4.6	1.7	0.9	5.7	2.9
Color compositions	FLUX	32.5	29.6	**39.2**	**68.2**	**40.0**	**41.9**
	SDXL	**45.0**	**44.2**	11.7	18.2	**40.0**	31.8
	SD1.5	2.5	6.2	1.7	1.4	11.4	4.6
Overall Effect	FLUX	8.3	7.3	**32.5**	**51.8**	16.2	23.2
	SDXL	**78.3**	**72.3**	27.5	32.3	**66.7**	55.4
	SD1.5	2.5	9.2	1.7	2.7	14.3	6.1

Note: Values represent vote percentages (%). Bold values indicate the model with the highest vote percentage for the corresponding dimension within each category. The numbers of participants were 20 for Headwear, 20 for Daily Necessities, 20 for Accessories, 20 for Headgear and Sashes, and 21 for Ritual Objects.

**Table 24:** Kendall’s W for inter-rater agreement by category: full sample.

Category	Form and Structure	Decorative Patterns	Material Appearance	Color Composition	Overall Effect
Headwear	0.131	**0.652**	0.250	0.179	**0.599**
Headgear and sashes	0.040	0.065	**0.534**	0.413	0.211
Accessories	0.250	0.249	**0.359**	0.216	0.133
Daily necessities	0.247	0.429	0.195	0.138	**0.462**
Ritual Objects	0.108	0.259	**0.281**	0.089	0.273
Equal-weighted average	0.155	0.331	0.324	0.207	**0.336**

Note: Bold values indicate the dimension with the highest W value within each category.

**Table 25:** Kendall’s W for inter-rater agreement by category after excluding “All Are Good” and “None Are Good” responses.

Category	Form and Structure	Decorative Patterns	Material Appearance	Color Coordination	Overall Effect	Removal Rate
Headwear	0.161	**0.738**	0.283	0.224	0.672	10.8–20.0%
Headgear and Sashes	0.046	0.074	**0.591**	0.471	0.243	9.5–13.2%
Accessories	0.417	0.403	**0.581**	0.410	0.216	38.3–47.5%
Daily necessities	0.308	0.493	0.226	0.173	**0.520**	11.2–20.0%
Ritual Objects	0.112	0.269	**0.292**	0.098	0.281	2.9–8.6%
Equal-weighted average	0.209	**0.396**	0.395	0.275	0.386	—

Note: This table includes only responses in which participants explicitly selected one of the three generated images (labeled A, B, or C). The exclusion rate represents the range of the combined percentages of “All Are Good” and “None Are Good” responses across the evaluation dimensions within each category. Bold values indicate the dimension with the highest W value within each category.

**Table 26:** Equally weighted vote percentages by dimension across the five artifact categories.

Dimension	FLUX	SDXL	SD1.5
Form and structure	12.5%	**47.1%**	21.4%
Decorative Patterns	11.7%	**60.8%**	11.4%
Material appearance	**60.0%**	21.6%	2.9%
Color Coordination	**41.9%**	31.8%	4.6%
Overall Effect	23.2%	**55.4%**	6.1%

Note: Equally weighted averaging was performed by first calculating the vote percentage for each model within each artifact category and then averaging the percentages across the five categories with equal weights. Bold values indicate the model with the highest vote percentage for the corresponding dimension. The total number of questionnaire participation instances was 101, covering 41 evaluated artifacts.

**Table 27:** Spearman’s rank correlation analysis between objective metrics and subjective evaluations (*n* = 15).

Objective Metrics	Spearman’s ρ	*p*-Value	Direction of Correlation
SSIM	−0.466	0.080	Marginally significant negative correlation
LPIPS	+0.596	0.019	Significant positive correlation
Objective Composite Score	−0.564	0.028	Significant negative correlation

Note: *n* = 15 (5 artifact categories × 3 models). LPIPS is a negative metric, for which lower values indicate higher perceptual similarity. Its positive correlation with the subjective vote percentage indicates that, within the three models evaluated in this study, models with lower LPIPS values tended to receive lower subjective vote percentages. This direction was opposite to the negative correlation observed for SSIM. Because the three model observations within the same artifact category may not be statistically independent, the *p*-values are provided for descriptive reference only. The objective composite score was calculated as the equally weighted average of normalized SSIM and normalized (1 − LPIPS).

**Table 28:** Highest-scoring model for each artifact category under different subjective weights.

Subjective Weight w	Headwear	Headgear and Sashes	Accessories	Daily Necessities	Ritual Objects
0.3	SD1.5	SD1.5	SD1.5	SD1.5	SD1.5
0.5	SDXL	FLUX	FLUX	SDXL	SDXL
0.7	SDXL	FLUX	FLUX	SDXL	SDXL

**Table 29:** Comparison of SDXL_LoRA at 768 × 768 and 1024 × 1024 resolutions for the Headwear category (*n* = 11).

Metric	768 × 768	1024 × 1024	Wilcoxon *p*
SSIM	**0.523 ± 0.176 [0.427, 0.623]**	0.488 ± 0.124 [0.417, 0.554]	0.320
LPIPS	**0.614 ± 0.106 [0.556, 0.668]**	0.664 ± 0.056 [0.632, 0.691]	0.067
CLIP	30.11 ± 2.36 [28.6, 31.4]	**30.63 ± 2.23 [29.2, 31.8]**	0.206
Seed-to-seed SD (SSIM)	**0.152**	0.162	—

Note: *p*-values are the unadjusted *p*-values from the Wilcoxon signed-rank test; *n* = 11 artifacts. All comparisons yielded *p* > 0.05, indicating no statistically significant differences. Bold values indicate the better-performing resolution for the corresponding metric.

## Data Availability

The dataset presented in this study is available on IEEE DataPort at https://ieee-dataport.org/documents/dataset-gold-and-silver-artifacts-tomb-prince-liangzhuang (accessed on 3 October 2026). The dataset is provided under IEEE DataPort’s standard access terms and is available to authorized subscribers; researchers without a subscription may request access via the IEEE DataPort platform.

## Abbreviations

The following abbreviations are used in this manuscript:

LoRA	Low-Rank Adaptation
AIGC	AI-Generated Content
SD1.5	Stable Diffusion v1.5
SDXL	Stable Diffusion XL
FID	Fréchet Inception Distance
SSIM	Structural Similarity Index Measure
LPIPS	Learned Perceptual Image Patch Similarity
CLIP	Contrastive Language–Image Pretraining
GAN	Generative Adversarial Network
ICH	Intangible Cultural Heritage
LCM	Latent Consistency Model
CMBP-9	Chinese Miao Batik Patterns-9 Dataset
CFG	Classifier-Free Guidance
KID	Kernel Inception Distance
SEM-EDS	Scanning Electron Microscopy–Energy-Dispersive X-ray Spectroscopy
XRF	X-ray Fluorescence
EDXRF	Energy-Dispersive X-ray Fluorescence
CI	Confidence Interval
SD	Standard Deviation
SE	Standard Error

## Appendix A

**Table A1:** Prompt comparison table (using the headwear dataset as an example).

Cultural Relic ID	Prompt
1	binchai, gold side hairpin, long bar-shaped finial with pin shaft, filigree wire mesh texture, hollow openwork texture, gem bezel inlay texture, floral pattern, gold, pink gemstone, grayish-blue gemstone, warm golden with pink and grayish-blue gemstone accents
2	Fenxin, gold fan-shaped headpiece, with an obtuse triangular overall shape; Features a hollow, carved white jade center with a filigree frame; Incorporates filigree wire mesh, hollow openwork, and gemstone and jade bezel inlay textures; Adorned with a paired phoenix motif and floral patterns; Crafted from gold, white jade, pink gemstones, blue gemstones, green gemstones, and purple gemstones; Warm golden in color with multicolored gemstone accents
3	Jindianhua, gold floral hair ornament, overall shape of an eight-petal flower, flat sheet structure, filigree wire mesh texture, hollow openwork texture, floral pattern, gold, warm golden tone
4	yunyanbin, gold temple hairpin, cloud-shaped overall form, filigree base with evenly distributed claw-set gemstones, filigree wire mesh texture, hollow openwork texture, gem bezel inlay texture, cloud pattern, gold, pink gemstone, green gemstone, lavender-blue gemstone, warm golden with pink, green, and lavender-blue gemstone accents
5	Luanyanbin, gold temple hairpin, overall cloud shape, hollow-carved white jade center surrounded by gemstones, filigree wire mesh texture, hollow openwork texture, gem and jade bezel inlay texture, paired phoenix pattern, peony pattern, gold, white jade, turquoise, pink gemstone, lavender-blue gemstone, warm golden with white, turquoise, blue, green, pink, and lavender-blue accents
6	meihuazan, gold hairpin with gemstones, plum-blossom-shaped finial with pin shaft, filigree wire mesh texture, gem bezel inlay texture, plum-blossom pattern, gold, pink gemstone, warm golden with pink gemstone accents
7	Meijinzan, plain gold hairpin, small plum-blossom-shaped finial with a long pin shaft, smooth polished texture, gold, warm golden tone
8	taozan, gold hairpin with gemstones, peach-shaped finial with pin shaft, filigree wire mesh texture, hollow openwork texture, gem bezel inlay texture, peach and floral pattern, gold, pink gemstone, grayish-blue gemstone, warm golden with pink and grayish-blue gemstone accents
9	Xiangguazan, gold hairpin with gemstones, melon-shaped finial with pin shaft, ruyi-cloud-shaped decorations, filigree wire mesh texture, hollow openwork texture, gem and jade bezel inlay texture, hollow-carved white jade center, melon and floral pattern, gold, white jade, pink gemstone, purple gemstone, blue gemstone, turquoise, warm golden with white, pink, purple, blue, and turquoise gemstone accents
10	Jinfengzan, gold hairpin, three-dimensional phoenix finial with outstretched wings and pin shaft, filigree wire mesh texture, hollow openwork texture, phoenix pattern, gold, warm golden tone
11	Mudan Zan, gold hairpin, three-dimensional peony flower finial with layered petals and leaves and a short pin shaft, hammered embossed texture, peony pattern, gold, warm golden tone
